# Supplementary material for: Design, Synthesis, and In Vitro and In Vivo Antifungal Activity of Novel Triazoles Containing Phenylethynyl Pyrazole Side Chains
Source: Molecules. 2022 May 24;27(11):3370. doi: 10.3390/molecules27113370 (PMC9182106; doi:10.3390/molecules27113370)
Supplement: Supplementary file 1 [file molecules-27-03370-s001.zip › molecules-1717803-supplementary.pdf]

Design, synthesis, and *in vitro* and *in vivo* antifungal activity of novel triazoles containing phenylethynyl pyrazole side chains

Tingjunhong Ni [1,†], **Zichao Ding** [2,†], **Fei Xie** [2,†], **Yumeng Hao** [2], **Junhe Bao** [2], **Jingxiang Zhang** [3], **Shichong Yu** [2,*], **Yuanying Jiang** [1,*] and **Dazhi Zhang** [1, 2,*]

1 *Shanghai Tenth People's Hospital, Tongji University School of Medicine, Shanghai, 200072(China);*
2 *Department of Organic Chemistry, School of Pharmacy, Navy Medical University, PLA, 325 Guohe Road, Shanghai, 200433(China)*
3 *Center for New Drug Research, School of Pharmacy, Navy Medical University, PLA, 325 Guohe Road, Shanghai, 200433(China)*

Experimental section

Table S1. *In silico* ADME/T prediction of target compounds with fluconazole

ADME/T parameters	**5a**	**5b**	**5j**	**5k**	**6a**	**6c**	FCZ [g]
Aqueous solubility [a]	3	2	2	3	2	2	4
BBB penetration [b]	3	3	4	4	4	4	3
A log P98 [c]	2.751	3.477	4.54	3.516	5.315	4.986	0.75
PSA [d]	95.406	95.406	95.406	107.96	74.225	97.16	75.556
PPB [e]	13.3519	13.6593	14.1615	14.1868	12.9317	12.9428	10.2619
Ames mutagenicity	Non-mutangen	Non-mutangen	Non-mutangen	Non-mutangen	Non-mutangen	Non-mutangen	Non-mutangen
FDA rodent carcinogenicity	Non-carcinogen	Non-carcinogen	Non-carcinogen	Non-carcinogen	Non-carcinogen	Non-carcinogen	Non-carcinogen
Skin_Irritancy	Non-Irritant	Non-Irritant	Non-Irritant	Irritant	Non-Irritant	Non-Irritant	Non-Irritant
Skin_sensitization	Irritant	Irritant	Irritant	Irritant	Irritant	Irritant	Irritant
Mouse_Female_FDA [f]	Non-carcinogen	Non-carcinogen	Non-carcinogen	Non-carcinogen	Non-carcinogen	Non-carcinogen	Non-carcinogen

a. Level of aqueous solubility predicted: 0 (extremely low), 1 (very low, but possible), 2 (low), 3 (good), 4 (optimal), 5 (too soluble), 6 (warning: molecules with one or more unknown A log P calculations). **b**. BBB (blood brain barrier), level blood brain barrier penetration prediction: 0 (very high penetrate), 1 (high), 2 (medium), 3 (low), 4 (undefined). **c**. A log P98 (atom-based log P) (≦2.0 or ≧7.0: very low absorption). **d**. PSA (polar surface area) (>150: very low absorption). **e**. PPB, plasma

protein binding. **f**. FDA, food and drug administration. **g**. FCZ, fluconazole.

Table S2. Molecular docking results

Name	Total_Score	Crash	Polar	D_SCORE	PMF_SCORE	G_SCORE	CHEMSCORE	CSCORE	GLOBAL_CSCORE
5n_000	11.745	-4.2452	3.464	-231.471	-183.978	-407.2343	-55.8521	5	5
5r_000	11.0843	-4.2219	4.7783	-232.8047	-127.7105	-319.8405	-51.832	3	3
6e_000	10.8757	-3.8221	3.3801	-240.0142	-127.279	-382.0045	-54.1113	4	4
5l_000	10.8155	-2.6829	3.0209	-204.1809	-192.6758	-356.8454	-48.7757	5	4
5j_000	10.8064	-1.1889	2.8234	-196.2006	-163.392	-314.9442	-46.2631	3	2
6d_000	10.5224	-1.9085	2.7575	-191.5306	-158.0707	-320.2353	-46.2357	3	2
5m_000	10.4647	-2.0344	3.5237	-192.8891	-156.7563	-324.0264	-50.1495	2	3
5t_000	10.4124	-4.0664	2.9946	-234.6778	-185.0634	-380.6436	-50.6056	5	5
5v_000	10.4108	-2.1503	2.7814	-206.7711	-140.3433	-303.1159	-44.5942	3	1
6b_000	10.3222	-0.9324	3.5378	-183.6694	-166.0302	-301.1239	-47.3378	1	3
5g_000	10.3045	-3.0193	2.0801	-213.2841	-188.553	-359.2463	-47.9082	5	5
5k_000	10.2999	-3.5763	2.3933	-206.4885	-183.8428	-370.6327	-50.3029	5	4
5q_000	10.2567	-3.8899	3.5492	-213.9455	-156.7905	-349.9968	-51.1825	5	5
5s_000	10.2406	-1.9229	3.0851	-190.1714	-184.082	-317.4736	-48.1461	4	3
5e_000	10.1708	-3.0126	2.4779	-206.357	-186.4358	-332.5316	-44.3827	5	2
6c_000	10.1248	-1.813	2.6672	-194.2356	-160.455	-332.7596	-46.9072	3	3
5d_000	10.1161	-1.3494	2.8123	-184.229	-159.5544	-287.9425	-43.2091	1	2
5f_000	10.0983	-2.2073	2.1752	-207.0006	-195.4618	-344.8771	-46.8	5	4
5i_000	9.803	-1.6787	3.0832	-178.496	-193.8251	-300.9256	-46.0247	3	2
5o_000	9.7532	-2.98	4.0878	-206.7038	-181.7028	-338.3246	-50.0609	5	4
6a_000	9.7102	-4.6858	1.9642	-221.2759	-144.558	-375.3317	-50.9772	4	4
5p_000	9.656	-2.0368	3.5572	-188.223	-175.9137	-307.4477	-45.8275	2	2
5b_000	9.5251	-2.3038	2.6358	-180.8083	-160.2807	-309.3794	-42.6301	3	2
5c_000	9.4852	-2.8978	2.0824	-191.0534	-173.5106	-311.618	-43.6842	5	2
5h_000	9.4297	-0.916	2.8071	-171.1173	-166.9422	-275.6497	-40.1495	2	2
5u_000	9.2634	-2.0024	2.7255	-200.0667	-166.9224	-304.1454	-43.6003	2	2
5a_000	9.0062	-1.63	3.0165	-170.6214	-168.5682	-285.7326	-41.709	3	2

Chemistry

^{1}H and ^{13}C Nuclear magnetic resonance (NMR) spectra were reported in DMSO-d_6 unless otherwise indicated with a Bruker AC-300P spectrometer. Tetramethylsilane (TMS) was considered as the internal standard. Chemical shifts (δ values) and coupling constants (J values)

are given in ppm and Hz respectively. HPLC purity was determined by Agilent Technologies 6120 Quadrupole LC-MS. HRMS analyses were produced on an Aglient Technologies 6538 UHD Accurate-Mass Q-TOF LC/MS. Silica gel plates GF254 (Yantai Huanghai Chemical, China) were applied to thin-layer chromatography (TLC) analysis. All the solvents and reagents were purchased from commercial vendors and were used as received or dried prior to use as needed.

Procedure for the synthesis of 2-(2,4-difluorophenyl)-1-(4-iodo-1H-pyrazol-1-yl)-3-(1H-1,2,4-triazol-1-yl)propan-2-ol ***（2）***

To a solution of compound **1** (50 mmol) and 4-Iodo-1H-pyrazole (50 mmol) in DMF (150 mL) were added K_2CO_3 (100 mmol). The mixture was stirred continuously for 4 h and heated at 80 ℃. The reaction was monitored by TLC. After the reaction was finished, the mixture was cooled to room temperature, poured into ice water, then stirred for 1 hour. The product solid was filtered, and then dried at 50 ℃ to get compound **2** (15.5 g, yellow solid, yield 72 %).

^{1}H NMR (300 MHz, DMSO-d_6) δ 8.31 (s, 1H), 7.76 (d, J = 4.3 Hz, 2H), 7.41 (s, 1H), 7.19 (dd, J = 14.2, 7.8 Hz, 2H), 6.89 (dd, J = 11.7, 5.1 Hz, 1H), 6.29 (s, 1H), 4.74 - 4.44 (m, 4H).

^{13}C NMR (75 MHz, DMSO-d_6) δ 162.79, 159.56, 151.20, 145.53, 143.95, 136.19, 130.21, 124.01, 111.43, 104.34, 74.63, 58.23, 57.33, 55.35. HPLC purity: 97.0%.

Procedure for the synthesis of methyl 4-((1-(2-(2,4-difluorophenyl)-2-hydroxy-3-(1H-1,2,4-triazol-1-yl)propyl)-1H-pyrazol-4-yl)ethynyl)benzoate (3)

Under nitrogen, compound **2** (40 mmol) and Methyl 4-ethynylbenzoate (40 mmol) were dissolved in NMP (140 mL). To this solution was added CuI (20 mmol%), $Pd(PPh_3)_2Cl_2$ (5 mmol%) and DIEA (200 mmol). The mixture was degassed under nitrogen prior to heating at 60 ℃ for 6 h. The reaction was monitored by TLC. After the reaction was finished, the mixture was poured into ice water, and then extracted with ethyl acetate (3 × 250 mL). The organic phases were combined, washed with saturated aqueous sodium chloride solution (2 × 300 mL), dried over anhydrous Na_2SO_4 and evaporated under reduced pressure. The crude products were purified by chromatography on silica gel (PE: EA=20:1~5:1) get the compound **4** (14.57 g, yellow solid, yield 78 %).

^{1}H NMR (300 MHz, DMSO-d_6) δ 8.33 (s, 1H), 8.00 - 7.93 (m, 3H), 7.79 (s, 1H), 7.60 (d, J = 8.7 Hz, 4H), 7.20 (dd, J = 17.1, 8.1 Hz, 2H), 6.89 (t, J = 7.5 Hz, 1H), 6.35 (s, 1H), 4.76 - 4.49 (t, J = 14.8 Hz, 2H), 4.51 (d, J = 14.3 Hz, 2H), 3.85 (s, 3H). HPLC purity: 97.2%; HRMS (ESI) m/z calcd for $C_{24}H_{19}F_2N_5O_3$ $[M+H]^+$: 464.1534, found: 464.1536.

Procedure for the synthesis of 4-((1-(2-(2,4-difluorophenyl)-2-hydroxy-3-(1H-1,2,4-triazol-1-yl)propyl)-1H-pyrazol-4-yl)ethynyl)benzoic acid ***（4）***

To a solution of compound **3** (31 mmol) in a mixed solution of THF (100 mL) and H_2O (100 mL), were added LiOH (200 mmol). The mixture was stirred continuously for 6 h and heated at 50 ℃. The reaction was monitored by TLC. After the reaction was finished, THF was evaporated under reduced pressure. Aqueous hydrochloric acid (5 mol/L) was dropped into the remaining liquid to adjusted pH to 3-4. After stirring for 1 h, the precipitated solid was filtered and dried to get compound **5** (12.1 g, yellow solid, yield 67 %).

^{1}H NMR (300 MHz, DMSO-d_6) δ 13.06 (s, 1H), 8.0 - 7.94 (m, 4H), 7.59 (d, J = 11.2 Hz, 4H), 7.21 (t, J = 9.5 Hz, 2H), 6.90 (d, J = 7.6 Hz, 1H), 6.36 (s, 1H), 4.76 - 4.49 (m, 4H). HPLC purity: 98.2%; HRMS

(ESI) m/z calcd for $C_{23}H_{17}F_2N_5O_3$ $[M+H]^+$: 450.1377, found: 450.1384.

General procedure for the synthesis of target compounds (5a-5v)

To a solution of compound **4** (1.0 mmol) and amines (1.0 mmol) in DMF (5 mL), were added DIEA (2.0 mmol) and PyBOP (1.1 mmol). The mixture was stirred continuously for 4-8 h and heated at 50 ℃. The reaction was monitored by TLC. After the reaction was finished, the mixture was poured into ice water, and then extracted with ethyl acetate (3 × 20 mL). The organic phases were combined, washed with saturated aqueous sodium chloride solution (2 × 30 mL), dried over anhydrous Na_2SO_4 and evaporated under reduced pressure. The crude products were purified by reverse phase and then lyophilized to get the target compounds.

4-((1-(2-(2,4-difluorophenyl)-2-hydroxy-3-(1H-1,2,4-triazol-1-yl)propyl)-1H-pyrazol-4-yl)ethynyl)-N-methylbenzamide (5a)

(309 mg, yellow solid, yield 67 %), ^{1}H NMR (300 MHz, DMSO-*d6*) δ 8.50 (d, *J* = 4.7 Hz, 1H), 8.32 (s, 1H), 7.97 (s, 1H), 7.84 - 7.79 (m, 3H), 7.59 - 7.52 (m, 3H), 7.25 - 7.17 (m, 2H), 6.89 (t, *J* = 7.4 Hz, 1H), 6.33 (s, 1H), 4.71 (t, *J* = 15.2 Hz, 2H), 4.51 (d, *J* = 14.4 Hz, 2H), 2.78 (d, *J* = 4.4 Hz, 3H).HPLC purity: 93.3%; HRMS (ESI) m/z calcd for $C_{24}H_{20}F_2N_6O_2$ $[M+H]^+$: 463.1694, found: 463.1708.

4-((1-(2-(2,4-difluorophenyl)-2-hydroxy-3-(1H-1,2,4-triazol-1-yl)propyl)-1H-pyrazol-4-yl)ethynyl)-N-isopropylbenzamide (5b)

(343 mg, yellow solid, yield 70 %), ^{1}H NMR (300 MHz, DMSO-*d6*) δ 8.33 - 8.28 (m, 2H), 7.98 (s, 1H), 7.87 - 7.79 (m, 3H), 7.59 - 7.52 (m, 3H), 7.25 - 7.17 (m, 2H), 6.89 (t, *J* = 7.6 Hz, 1H), 6.35 (s, 1H), 4.71 (t, *J* = 14.6 Hz, 2H), 4.51 (d, *J* = 14.3 Hz, 2H), 4.12 - 4.06 (m, 1H), 1.16 (d, *J* = 6.5 Hz, 6H).
^{13}C NMR (75 MHz, DMSO-*d6*) δ 165.01, 162.39, 157.93, 151.27, 145.59, 141.65, 135.06, 134.58, 131.13, 130.25, 128.03, 125.69, 124.09, 123.91, 111.45, 111.21, 104.74, 104.38, 104.03, 101.82, 99.99, 89.78, 83.93, 74.60, 74.53, 58.22, 55.40, 41.52, 22.76. HPLC purity: 96.8%; HRMS (ESI) m/z calcd for $C_{26}H_{24}F_2N_6O_2$ $[M+H]^+$: 491.2007, found: 491.2008.

4-((1-(2-(2,4-difluorophenyl)-2-hydroxy-3-(1H-1,2,4-triazol-1-yl)propyl)-1H-pyrazol-4-yl)ethynyl)-N,N-dimethylbenzamide (5c)

(271 mg, yellow solid, yield 57 %), ^{1}H NMR (300 MHz, DMSO-*d6*) δ 8.32 (d, *J* = 3.7 Hz, 1H), 7.97 (s, 1H), 7.79 (s, 1H), 7.63 - 7.39 (m, 5H), 7.23 - 7.17 (m, 2H), 6.89 (t, *J* = 7.5 Hz, 1H), 6.34 (s, 1H), 4.76 – 4.66 (m, 2H), 4.51 (d, *J* = 14.7 Hz, 2H), 2.94 (d, *J* = 21.2 Hz, 6H).
^{13}C NMR (75 MHz, DMSO-*d6*) δ 169.86, 162.55, 159.39, 151.28, 145.58, 141.65, 136.47, 135.09, 132.52, 131.26, 130.74, 130.25, 127.83, 124.16, 123.88, 111.49, 111.22, 104.74, 104.40, 104.03, 101.84, 58.21, 55.39, 35.23. HPLC purity: 95.3%; HRMS (ESI) m/z calcd for $C_{25}H_{22}F_2N_6O_2$ $[M+H]^+$: 477.1850, found: 477.1855.

4-((1-(2-(2,4-difluorophenyl)-2-hydroxy-3-(1H-1,2,4-triazol-1-yl)propyl)-1H-pyrazol-4-yl)ethynyl)-N-(pentan-3-yl)benzamide (5d)

(326 mg, yellow solid, yield 63 %), ^{1}H NMR (300 MHz, DMSO-*d6*) δ 8.33 (s, 1H), 8.12 (d, *J* = 8.7 Hz, 1H), 7.98 (s, 1H), 7.86 (d, *J* = 8.3 Hz, 2H), 7.79 (s, 1H), 7.59 (s, 1H), 7.54 (d, *J* = 8.3 Hz, 2H), 7.24 - 7.16 (m, 2H), 6.89 (t, *J* = 7.4 Hz, 1H), 6.35 (s, 1H), 4.71 (t, *J* = 14.6 Hz, 2H), 4.51 (d, *J* = 14.4 Hz, 2H), 3.83 - 3.71 (m, 1H), 1.58 - 1.40 (m, 4H), 0.84 (t, *J* = 7.3 Hz, 6H).

^{13}C NMR (75 MHz, DMSO-d_6) δ 165.87, 161.35, 159.18, 151.27, 145.59, 141.65, 135.06, 134.74, 131.15, 130.20, 128.02, 125.64, 124.08, 123.86, 111.46, 104.39, 101.81, 99.99, 89.81, 83.87, 74.59, 74.52, 58.16, 55.37, 52.62, 27.35, 11.09. HPLC purity: 95.9%; HRMS (ESI) m/z calcd for $C_{28}H_{28}F_2N_6O_2$ $[M+H]^+$: 519.2320, found: 519.2330.

4-((1-(2-(2,4-difluorophenyl)-2-hydroxy-3-(1H-1,2,4-triazol-1-yl)propyl)-1H-pyrazol-4-yl)ethynyl)-N-((R)-1-hydroxybutan-2-yl)benzamide (5e)

(275 mg, yellow solid, yield 53 %), ^1H NMR (300 MHz, DMSO-d_6) δ 8.33 (s, 1H), 8.09 (d, J = 8.5 Hz, 1H), 7.98 (s, 1H), 7.87 (d, J = 8.3 Hz, 2H), 7.79 (s, 1H), 7.59 (s, 1H), 7.54 (d, J = 8.3 Hz, 2H), 7.25 - 7.17 (m, 2H), 6.92 - 6.87 (m, 1H), 6.35 (s, 1H), 4.76 - 4.67 (m, 3H), 4.51 (d, J = 14.3 Hz, 2H), 3.86 (brs, 1H), 3.47 - 3.38 (m, 4H), 1.73 - 1.59 (m, 1H), 1.49 - 1.39 (m, 1H), 0.86 (t, J = 7.4 Hz, 3H). HPLC purity: 96.3%; HRMS (ESI) m/z calcd for $C_{27}H_{26}F_2N_6O_3$ $[M+H]^+$: 521.2112, found: 521.2110.

(4-((1-(2-(2,4-difluorophenyl)-2-hydroxy-3-(1H-1,2,4-triazol-1-yl)propyl)-1H-pyrazol-4-yl)ethynyl)phenyl)(4-hydroxypiperidin-1-yl)methanone (5f)

(383 mg, yellow solid, yield 72 %), ^1H NMR (300 MHz, DMSO-d_6) δ 8.33 (s, 1H), 7.98 (s, 1H), 7.79 (s, 1H), 7.59 (s, 1H), 7.52 (d, J = 7.8 Hz, 2H), 7.38 (d, J = 7.8 Hz, 2H), 7.24 - 7.16 (m, 2H), 6.89 (t, J = 8.1 Hz, 1H), 6.36 (s, 1H), 4.83 - 4.65 (m, 3H), 4.51 (d, J = 14.5 Hz, 2H), 3.99 (brs, 1H), 3.74 (brs, 1H), 3.44 (brs, 1H), 3.15 (brs, 2H), 1.74 (brs, 2H), 1.36 (brs, 2H).

^{13}C NMR (75 MHz, DMSO-d_6) δ 168.68, 164.02, 160.75, 157.85, 151.26, 145.59, 141.65, 136.35, 135.10, 131.39, 130.24, 127.56, 124.15, 123.92, 111.45, 111.22, 104.75, 104.40, 104.03, 101.83, 99.99, 89.66, 83.16, 74.58, 74.51, 65.87, 58.20, 55.38, 45.04, 34.85. HPLC purity: 95.7%; HRMS (ESI) m/z calcd for $C_{28}H_{26}F_2N_6O_3$ $[M+H]^+$: 533.2112, found: 533.2120.

(4-((1-(2-(2,4-difluorophenyl)-2-hydroxy-3-(1H-1,2,4-triazol-1-yl)propyl)-1H-pyrazol-4-yl)ethynyl)phenyl)((R)-3-hydroxypiperidin-1-yl)methanone (5g)

(313 mg, yellow solid, yield 59 %), ^1H NMR (300 MHz, DMSO-d_6) δ 8.33 (s, 1H), 7.98 (s, 1H), 7.79 (s, 1H), 7.62 - 7.40 (m, 5H), 7.25 - 7.17 (m, 2H), 6.89 (t, J = 8.4 Hz, 1H), 6.36 (s, 1H), 4.94 (d, J = 43.1 Hz, 1H), 4.87 - 4.66 (m, 2H), 4.52 (d, J = 14.2 Hz, 2H), 3.96 (d, J = 115.4 Hz, 1H), 3.96 (d, J = 115.4 Hz, 1H), 3.51 (s, 1H), 3.25 - 2.85 (m, 2H), 1.85 - 1.41 (m, 4H). HPLC purity: 95.3%; HRMS (ESI) m/z calcd for $C_{28}H_{26}F_2N_6O_3$ $[M+H]^+$: 533.2112, found: 533.2117.

(4-((1-(2-(2,4-difluorophenyl)-2-hydroxy-3-(1H-1,2,4-triazol-1-yl)propyl)-1H-pyrazol-4 yl)ethynyl)phenyl)((R)-3-hydroxypyrrolidin-1-yl)methanone (5h)

(316 mg, yellow solid, yield 61 %), ^1H NMR (300 MHz, DMSO-d_6) δ 8.32 (d, J = 6.4 Hz, 1H), 7.98 (s, 1H), 7.80 (s, 1H), 7.59 (s, 1H), 7.51 (s, 4H), 7.25 - 7.17 (m, 2H), 6.92 - 6.87 (m, 1H), 6.36 (s, 1H), 5.00 (dd, J = 21.6, 3.1 Hz, 1H), 4.77 - 4.66 (m, 2H), 4.51 (d, J = 14.6 Hz, 2H), 4.28 (d, J = 27.1 Hz, 1H), 3.55 (d, J = 10.5 Hz, 3H), 3.18 (d, J = 10.9 Hz, 1H), 1.96 - 1.81 (m, 2H).

^{13}C NMR (75 MHz, DMSO-d_6) δ 168.19, 162.66, 159.90, 151.29, 145.63, 141.66, 136.85, 135.11, 131.20, 130.20, 128.03, 127.96, 124.59, 124.05, 123.92, 123.87, 111.32, 104.38, 101.83, 89.71, 83.39, 83.35, 79.63, 74.59, 74.52, 69.84, 68.45, 58.21, 58.16, 57.44, 55.33, 54.85, 47.27, 44.62, 34.82, 32.61. HPLC purity: 94.9%; HRMS (ESI) m/z calcd for $C_{27}H_{24}F_2N_6O_3$ $[M+H]^+$: 519.1956, found: 519.1960.

(4-((1-(2-(2,4-difluorophenyl)-2-hydroxy-3-(1H-1,2,4-triazol-1-yl)propyl)-1H-pyrazol-4-yl)ethyn

yl)phenyl)(morpholino)methanone (5i)

(404 mg, yellow solid, yield 78 %), ^{1}H NMR (300 MHz, DMSO-d_6) δ 8.32 (s, 1H), 7.98 (s, 1H), 7.79 (s, 1H), 7.59 - 7.51 (m, 3H), 7.42 (d, J = 7.9 Hz, 2H), 7.25 - 7.16 (m, 2H), 6.89 (t, J = 8.1 Hz, 1H), 6.34 (s, 1H), 4.76 - 4.55 (m, 2H), 4.51 (d, J = 14.4 Hz, 2H), 3.59 (s, 8H). HPLC purity: 93.9%; HRMS (ESI) m/z calcd for $C_{27}H_{24}F_2N_6O_3$ $[M+H]^+$: 519.1956, found: 519.1961.

4-((1-(2-(2,4-difluorophenyl)-2-hydroxy-3-(1H-1,2,4-triazol-1-yl)propyl)-1H-pyrazol-4-yl)ethynyl)-N-(4-fluorobenzyl)benzamide (5j)

(439 mg, yellow solid, yield 79 %), ^{1}H NMR (300 MHz, DMSO-d_6) δ 9.13 (t, J = 5.9 Hz, 1H), 8.34 (s, 1H), 7.98 - 7.80 (m, 4H), 7.59 - 7.55 (m, 3H), 7.38 - 7.33 (m, 2H), 7.24 - 7.12 (m, 4H), 6.89 (t, J = 7.5 Hz, 1H), 6.34 (s, 1H), 4.71 (t, J = 15.2 Hz, 2H), 4.54 - 4.44 (m, 4H). HPLC purity: 94.8%; HRMS (ESI) m/z calcd for $C_{30}H_{23}F_3N_6O_2$ $[M+H]^+$: 557.1913, found: 557.1920.

4-((1-(2-(2,4-difluorophenyl)-2-hydroxy-3-(1H-1,2,4-triazol-1-yl)propyl)-1H-pyrazol-4-yl)ethynyl)-N-(furan-2-ylmethyl)benzamide (5k)

(264 mg, yellow solid, yield 50 %), ^{1}H NMR (300 MHz, DMSO-d_6) δ 9.05 (t, J = 5.4 Hz, 1H), 8.33 (s, 1H), 7.98 (s, 1H), 7.88 (d, J = 8.3 Hz, 2H), 7.79 (s, 1H), 7.59 - 7.53 (m, 4H), 7.24 - 7.15 (m, 2H), 6.89 (t, J = 8.5 Hz, 1H), 6.34 (t, J = 16.6 Hz, 3H), 4.71 (t, J = 14.9 Hz, 2H), 4.53 - 4.45 (m, 4H).
^{13}C NMR (75 MHz, DMSO-d_6) δ 165.78, 163.51, 159.86, 152.74, 151.27, 145.59, 142.51, 141.67, 135.12, 133.83, 131.27, 130.19, 128.10, 126.06, 124.08, 111.35, 110.94, 107.38, 104.39, 101.76, 89.72, 84.16, 74.58, 74.51, 58.21, 55.38, 36.54. HPLC purity: 95.8%; HRMS (ESI) m/z calcd for $C_{28}H_{22}F_2N_6O_3$ $[M+H]^+$: 529.1799, found: 529.1811.

4-((1-(2-(2,4-difluorophenyl)-2-hydroxy-3-(1H-1,2,4-triazol-1-yl)propyl)-1H-pyrazol-4-yl)ethynyl)-N-(pyridin-2-ylmethyl)benzamide (5l)

(361 mg, yellow solid, yield 67 %), ^{1}H NMR (300 MHz, DMSO-d_6) δ 9.19 (s, 1H), 8.50 (s, 1H), 8.32 (s, 1H), 7.98 - 7.56 (m, 8H), 7.31 - 7.20 (m, 4H), 6.89 (s, 1H), 6.34 (s, 1H), 4.75 - 3.48 (m, 6H). HPLC purity: 96.8%; HRMS (ESI) m/z calcd for $C_{29}H_{23}F_2N_7O_2$ $[M+H]^+$: 540.1959, found: 540.1963.

4-((1-(2-(2,4-difluorophenyl)-2-hydroxy-3-(1H-1,2,4-triazol-1-yl)propyl)-1H-pyrazol-4-yl)ethynyl)-N-(4-fluorophenethyl)benzamide (5m)

(370 mg, yellow solid, yield 65 %), ^{1}H NMR (300 MHz, DMSO-d_6) δ 8.63 (t, J = 5.2 Hz, 1H), 8.35 (s, 1H), 7.98 (s, 1H), 7.81 (d, J = 8.2 Hz, 3H), 7.59 - 7.53 (m, 3H), 7.29 - 7.08 (m, 6H), 6.89 (t, J = 7.5 Hz, 1H), 6.35 (s, 1H), 4.77 - 4.66 (m, 2H), 4.51 (d, J = 14.4 Hz, 2H), 3.56-3.44 (m, 2H), 2.83 (t, J = 7.1 Hz, 2H). HPLC purity: 95.4%; HRMS (ESI) m/z calcd for $C_{31}H_{25}F_3N_6O_2$ $[M+H]^+$: 571.2069, found: 571.2070.

4-((1-(2-(2,4-difluorophenyl)-2-hydroxy-3-(1H-1,2,4-triazol-1-yl)propyl)-1H-pyrazol-4-yl)ethynyl)-N-(3-phenylpropyl)benzamide (5n)

(401 mg, yellow solid, yield 71 %), ^{1}H NMR (300 MHz, DMSO-d_6) δ 8.55 (t, J = 5.1 Hz, 1H), 8.33 (s, 1H), 7.98 (s, 1H), 7.87 - 7.80 (m, 3H), 7.59 - 7.53 (m, 3H), 7.31 - 7.17 (m, 7H), 6.89 (t, J = 7.5 Hz, 1H), 6.35 (s, 1H), 4.71 (t, J = 14.8 Hz, 2H), 4.51 (d, J = 14.4 Hz, 2H), 3.35 - 3.27 (m, 2H), 2.63 (t, J = 7.5 Hz, 2H), 1.87- 1.66 (m, 2H).
^{13}C NMR (75 MHz, DMSO-d_6) δ 165.91, 162.55, 159.39, 151.27, 145.59, 142.21, 141.66, 135.09,

134.43, 131.22, 130.24, 128.78, 128.74, 127.96, 126.19, 125.79, 124.09, 123.87, 111.45, 111.18, 104.74, 104.39, 104.03, 101.81, 89.77, 83.98, 74.60, 74.53, 58.23, 55.39, 33.11, 31.26. HPLC purity: 96.8%; HRMS (ESI) m/z calcd for $C_{32}H_{28}F_2N_6O_2$ $[M+H]^+$: 567.2320, found: 567.2331.

4-((1-(2-(2,4-difluorophenyl)-2-hydroxy-3-(1H-1,2,4-triazol-1-yl)propyl)-1H-pyrazol-4-yl)ethynyl)-N-(4-fluorophenyl)benzamide (5o)

(373 mg, yellow solid, yield 69 %), ^1H NMR (300 MHz, DMSO-d_6) δ 10.36 (s, 1H), 8.38 (s, 1H), 8.00 - 7.96 (m, 3H), 7.81 - 7.80 (m, 3H), 7.62 (d, J = 6.4 Hz, 3H), 7.20 (m, 4H), 6.90 (t, J = 7.8 Hz, 1H), 6.35 (s, 1H), 4.72 (t, J = 15.2 Hz, 2H), 4.52 (d, J = 14.4 Hz, 2H).

^{13}C NMR (75 MHz, DMSO-d_6) δ 165.09, 160.67, 157.63, 141.70, 135.85, 135.16, 134.43, 131.32, 130.21, 128.45, 126.38, 124.09, 122.74, 122.63, 115.82, 115.52, 111.46, 111.18, 105.11, 104.39, 104.04, 101.75, 89.73, 84.42, 74.59, 58.18, 55.37. HPLC purity: 96.2%; HRMS (ESI) m/z calcd for $C_{29}H_{21}F_3N_6O_2$ $[M+H]^+$: 543.1756, found: 543.1768.

N-(4-chlorophenyl)-4-((1-(2-(2,4-difluorophenyl)-2-hydroxy-3-(1H-1,2,4-triazol-1-yl)propyl)-1H-pyrazol-4-yl)ethynyl)benzamide (5p)

(463 mg, yellow solid, yield 83 %), ^1H NMR (300 MHz, DMSO-d_6) δ 10.43 (s, 1H), 8.33 (s, 1H), 8.00 - 7.95 (m, 3H), 7.82 (d, J = 9.0 Hz, 3H), 7.62 (d, J = 7.4 Hz, 3H), 7.42 (d, J = 8.8 Hz, 2H), 7.25 - 7.17 (m, 2H), 6.90 (t, J = 8.3 Hz, 1H), 6.34 (s, 1H), 4.77 - 4.67 (m, 2H), 4.52 (d, J = 14.3 Hz, 2H). HPLC purity: 95.4%; HRMS (ESI) m/z calcd for $C_{29}H_{21}ClF_2N_6O_2$ $[M+H]^+$: 559.1461, found: 559.1489.

4-((1-(2-(2,4-difluorophenyl)-2-hydroxy-3-(1H-1,2,4-triazol-1-yl)propyl)-1H-pyrazol-4-yl)ethynyl)-N-(4-(trifluoromethyl)phenyl)benzamide (5q)

(319 mg, yellow solid, yield 54 %), ^1H NMR (300 MHz, DMSO-d_6) δ 10.65 (s, 1H), 8.34 (s, 1H), 8.03 - 7.98 (m, 5H), 7.81 - 7.62 (m, 6H), 7.25 - 7.19 (m, 2H), 6.90 (t, J = 7.6 Hz, 1H), 6.36 (s, 1H), 4.77 - 4.67 (m, 2H), 4.52 (d, J = 14.4 Hz, 2H).

^{13}C NMR (75 MHz, DMSO-d_6) δ 165.69, 162.65, 159.39, 151.34, 143.18, 141.71, 135.19, 134.08, 131.36, 130.24, 128.64, 126.71, 126.64, 124.38, 124.03, 123.53, 120.60, 111.49, 104.40, 101.71, 89.70, 84.65, 74.59, 74.52, 58.17, 55.33. HPLC purity: 95.1%; HRMS (ESI) m/z calcd for $C_{30}H_{21}F_5N_6O_2$ $[M+H]^+$: 593.1724, found: 593.1741.

4-((1-(2-(2,4-difluorophenyl)-2-hydroxy-3-(1H-1,2,4-triazol-1-yl)propyl)-1H-pyrazol-4-yl)ethynyl)-N-(4-(trifluoromethoxy)phenyl)benzamide (5r)

(291 mg, yellow solid, yield 48 %), ^1H NMR (300 MHz, DMSO-d_6) δ 10.50 (s, 1H), 8.34 (s, 1H), 8.00 - 7.80 (m, 6H), 7.63 (d, J = 8.7 Hz, 3H), 7.37 (d, J = 8.5 Hz, 2H), 7.25 - 7.20 (m, 2H), 6.90 (td, J = 8.5, 2.0 Hz, 1H), 6.35 (s, 1H), 4.77 - 4.67 (m, 2H), 4.52 (d, J = 14.4 Hz, 2H).

^{13}C NMR (75 MHz, DMSO-d_6) δ 165.34, 162.18, 159.48, 151.33, 144.39, 141.70, 138.74, 135.17, 134.25, 131.34, 130.20, 128.53, 126.54, 124.03, 122.17, 121.95, 118.93, 111.45, 104.40, 101.73, 89.71, 84.52, 74.60, 58.18, 55.41, 26.80. HPLC purity: 93.2%; HRMS (ESI) m/z calcd for $C_{30}H_{21}F_5N_6O_3$ $[M+H]^+$: 609.1673, found: 609.1682.

N-(2,4-difluorophenyl)-4-((1-(2-(2,4-difluorophenyl)-2-hydroxy-3-(1H-1,2,4-triazol-1-yl)propyl)-1H-pyrazol-4-yl)ethynyl)benzamide (5s)

(341 mg, yellow solid, yield 61 %), ^1H NMR (300 MHz, DMSO-d_6) δ 10.21 (s, 1H), 8.33 (s, 1H), 8.00

- 7.97 (m, 3H), 7.80 (s, 1H), 7.63 - 7.55 (m, 4H), 7.40 - 7.33 (m, 1H), 7.25 - 7.10 (m, 3H), 6.90 (t, *J* = 7.4 Hz, 1H), 6.35 (s, 1H), 4.77 - 4.68 (m, 2H), 4.52 (d, *J* = 14.4 Hz, 2H). HPLC purity: 94.1%; HRMS (ESI) m/z calcd for $C_{29}H_{20}F_4N_6O_2$ $[M+H]^+$: 561.1662, found: 561.1677.

4-((1-(2-(2,4-difluorophenyl)-2-hydroxy-3-(1H-1,2,4-triazol-1-yl)propyl)-1H-pyrazol-4-yl)ethynyl)-N-(4-(2,2,2-trifluoroethoxy)phenyl)benzamide (5t)

(447 mg, yellow solid, yield 72 %), ^{1}H NMR (300 MHz, DMSO-d_6) δ 10.28 (s, 1H), 8.34 (s, 1H), 8.00 - 7.95 (m, 3H), 7.80 - 7.60 (m, 6H), 7.25 - 7.19 (m, 2H), 7.06 (d, *J* = 8.9 Hz, 2H), 6.90 (t, *J* = 8.3 Hz, 1H), 6.36 (s, 1H), 4.78 - 4.67 (m, 4H), 4.52 (d, *J* = 14.4 Hz, 2H).

^{13}C NMR (75 MHz, DMSO-d_6) δ 164.88, 162.65, 159.40, 153.65, 151.31, 145.59, 141.69, 135.15, 134.57, 133.99, 131.31, 130.33, 128.40, 126.25, 124.08, 123.90, 122.68, 122.33, 119.00, 115.41, 111.49, 104.39, 101.76, 89.75, 84.35, 74.59, 74.52,65.31, 58.23, 55.33. HPLC purity: 94.8%; HRMS (ESI) m/z calcd for $C_{31}H_{23}F_5N_6O_3$ $[M+H]^+$: 623.1830, found: 623.1845.

4-((1-(2-(2,4-difluorophenyl)-2-hydroxy-3-(1H-1,2,4-triazol-1-yl)propyl)-1H-pyrazol-4-yl)ethynyl)-N-(4-(2,2,3,3-tetrafluoropropoxy)phenyl)benzamide (5u)

(490 mg, yellow solid, yield 75 %), ^{1}H NMR (300 MHz, DMSO-d_6) δ 10.27 (s, 1H), 8.34 (s, 1H), 8.00 - 7.95 (m, 3H), 7.80 - 7.60 (m, 6H), 7.25 - 7.19 (m, 2H), 7.06 (d, *J* = 8.8 Hz, 2H), 6.92 - 6.47 (m, 2H), 6.36 (s, 1H), 4.77 - 4.49 (m, 6H). HPLC purity: 95.0%; HRMS (ESI) m/z calcd for $C_{32}H_{24}F_6N_6O_3$ $[M+H]^+$: 655.1892, found: 655.1902.

4-((1-(2-(2,4-difluorophenyl)-2-hydroxy-3-(1H-1,2,4-triazol-1-yl)propyl)-1H-pyrazol-4-yl)ethynyl)-N-(4-(2,2,3,3,3-pentafluoropropoxy)phenyl)benzamide (5v)

(342 mg, yellow solid, yield 51 %), ^{1}H NMR (300 MHz, DMSO-d_6) δ 10.27 (s, 1H), 8.33 (s, 1H), 8.00 - 7.75 (m, 3H), 7.80 - 7.61 (m, 6H), 7.25 - 7.20 (m, 2H), 7.07 (d, *J* = 8.9 Hz, 2H), 6.90 (t, *J* = 7.7 Hz, 1H), 6.35 (s, 1H), 4.86 - 4.67 (m, 4H), 4.52 (d, *J* = 14.3 Hz, 2H). HPLC purity: 95.1%; HRMS (ESI) m/z calcd for $C_{32}H_{23}F_7N_6O_3$ $[M+H]^+$: 673.1798, found: 673.1817.

General procedure for the synthesis of target compounds (6a-6e)

Under nitrogen, compound **2** (1.0 mmol) and alkynes (1.0 mmol) were dissolved in NMP (10 ml). To this solution was added CuI (20 mmol%), $Pd(PPh_3)_2Cl_2$ (5 mmol%) and DIEA (5 mmol). The mixture was degassed under nitrogen prior to heating at 60 ℃ for 6 h. The reaction was monitored by TLC. After the reaction was finished, the mixture was poured into ice water, and then extracted with ethyl acetate (3 × 20 mL). The organic phases were combined, washed with saturated aqueous sodium chloride solution (2 × 30 mL), dried over anhydrous Na_2SO_4 and evaporated under reduced pressure. The crude products were purified by chromatography on silica gel (PE: EA=10:1~5:1).

2-(2,4-difluorophenyl)-1-(4-((4-((4-fluorobenzyl)oxy)phenyl)ethynyl)-1H-pyrazol-1-yl)-3-(1H-1,2,4-triazol-1-yl)propan-2-ol (6a)

(210 mg, white solid, yield 32 %), ^{1}H NMR (300 MHz, DMSO-d_6) δ 8.32 (s, 1H), 7.90 (s, 1H), 7.78 (s, 1H), 7.52 - 7.38 (m, 5H), 7.24 - 7.15 (m, 4H), 7.01 (d, *J* = 8.8 Hz, 2H), 6.88 (td, *J* = 8.5, 2.3 Hz, 1H), 6.32 (s, 1H), 5.09 (s, 2H), 4.75 - 4.63 (m, 2H), 4.49 (d, *J* = 14.4 Hz, 2H). HPLC purity: 94.0%; HRMS (ESI) m/z calcd for $C_{29}H_{22}F_3N_5O_2$ $[M+H]^+$: 530.1804, found: 530.1804.

1-(4-((4-((4-chlorobenzyl)oxy)phenyl)ethynyl)-1H-pyrazol-1-yl)-2-(2,4-difluorophenyl)-3-(1H-1,2,4-triazol-1-yl)propan-2-ol (6b)

(315 mg, white solid, yield 47 %), ^{1}H NMR (300 MHz, DMSO-*d*$_6$) δ 8.31 (s, 1H), 7.89 (s, 1H), 7.77 (s, 1H), 7.52 - 7.38 (m, 7H), 7.23 - 7.15 (m, 2H), 7.00 (d, *J* = 8.8 Hz, 2H), 6.88 (td, *J* = 8.5, 2.4 Hz, 1H), 6.31 (s, 1H), 5.12 (s, 2H), 4.74 - 4.62 (m, 2H), 4.48 (d, *J* = 15.6 Hz, 2H).

^{13}C NMR (75 MHz, DMSO-*d*$_6$) δ 162.63,159.38, 158.57, 151.25, 145.59, 141.44, 136.74, 136.27, 134.62, 133.02, 132.98, 130.21, 130.03, 128.94, 123.95, 121.55, 115.65, 115.53, 111.44, 104.37, 102.39, 80.41, 74.61, 68.93, 58.11, 55.39. HPLC purity: 93.7%; HRMS (ESI) m/z calcd for $C_{29}H_{22}ClF_2N_5O_2$ $[M+H]^+$: 546.1508, found: 546.1516.

4-((4-((1-(2-(2,4-difluorophenyl)-2-hydroxy-3-(1H-1,2,4-triazol-1-yl)propyl)-1H-pyrazol-4-yl)ethynyl)phenoxy)methyl)benzonitrile (6c)

(257 mg, white solid, yield 48 %), ^{1}H NMR (300 MHz, DMSO-*d*$_6$) δ 8.33 (s, 1H), 7.91 - 7.79 (m, 4H), 7.64 (d, *J* = 8.0 Hz, 2H), 7.53 (s, 1H), 7.42 (d, *J* = 8.5 Hz, 2H), 7.24 - 7.16 (m, 2H), 7.03 (d, *J* = 8.6 Hz, 2H), 6.89 (t, *J* = 7.7 Hz, 1H), 6.34 (s, 1H), 5.25 (s, 2H), 4.76 - 4.64 (m, 2H), 4.50 (d, *J* = 14.6 Hz, 2H).

^{13}C NMR (75 MHz, DMSO-*d*$_6$) δ 162.65, 159.39, 158.37, 151.25, 145.58, 143.04, 141.45, 134.63, 133.06, 132.91, 130.26, 128.59, 124.12, 123.95, 119.20, 115.73, 115.66, 111.48, 111.18, 111.03, 104.74, 104.39, 104.02, 102.36, 89.93, 80.49, 74.60, 74.54, 68.81, 58.17, 55.32. HPLC purity: 98.5%; HRMS (ESI) m/z calcd for $C_{30}H_{22}F_2N_6O_2$ $[M+H]^+$: 537.1850, found: 537.1859.

2-(2,4-difluorophenyl)-1-(1H-1,2,4-triazol-1-yl)-3-(4-((4-((4-(trifluoromethyl)benzyl)oxy)phenyl)ethynyl)-1H-pyrazol-1-yl)propan-2-ol (6d)

(302 mg, white solid, yield 41 %), ^{1}H NMR (300 MHz, DMSO-*d*$_6$) δ 8.31 (s, 1H), 7.89 (s, 1H), 7.76 (d, *J* = 8.8 Hz, 3H), 7.66 (d, *J* = 8.1 Hz, 2H), 7.52 (s, 1H), 7.41 (d, *J* = 8.7 Hz, 2H), 7.24 - 7.16 (m, 2H), 7.02 (d, *J* = 8.7 Hz, 2H), 6.91 - 6.84 (m, 1H), 6.30 (s, 1H), 5.25 (s, 2H), 4.75 - 4.63 (m, 2H), 4.49 (d, *J* = 14.4 Hz, 2H).

^{13}C NMR (75 MHz, DMSO-*d*$_6$) δ 162.81, 159.21, 158.46, 151.25, 145.59, 142.15, 141.45, 134.63, 133.06, 130.26, 129.07, 128.65, 128.52, 126.49, 125.80, 124.13, 123.92, 122.89, 115.68, 111.44, 104.37, 102.38, 89.94, 80.45, 74.62, 74.55, 68.88, 58.12, 55.33. HPLC purity: 97.3%; HRMS (ESI) m/z calcd for $C_{30}H_{22}F_5N_5O_2$ $[M+H]^+$: 580.1772, found: 580.1751.

2-(2,4-difluorophenyl)-1-(1H-1,2,4-triazol-1-yl)-3-(4-((4-((4-(trifluoromethoxy)benzyl)oxy)phenyl)ethynyl)-1H-pyrazol-1-yl)propan-2-ol (6e)

(244 mg, white solid, yield 39 %), ^{1}H NMR (300 MHz, DMSO-*d*$_6$) δ 8.31 (s, 1H), 7.89 (s, 1H), 7.77 (s, 1H), 7.59 - 7.52 (m, 3H), 7.46 - 7.37 (m, 4H), 7.23 - 7.15 (m, 2H), 7.02 (d, *J* = 8.8 Hz, 2H), 6.88 (td, *J* = 8.5, 2.4 Hz, 1H), 6.31 (s, 1H), 5.16 (s, 2H), 4.74 - 4.62 (m, 2H), 4.48 (d, *J* = 15.3 Hz, 2H). HPLC purity: 96.8%; HRMS (ESI) m/z calcd for $C_{30}H_{22}F_5N_5O_3$ $[M+H]^+$: 596.1721, found: 596.1702.

^{1}H NMR, ^{13}C NMR and HRMS Spectrum of Some Compounds

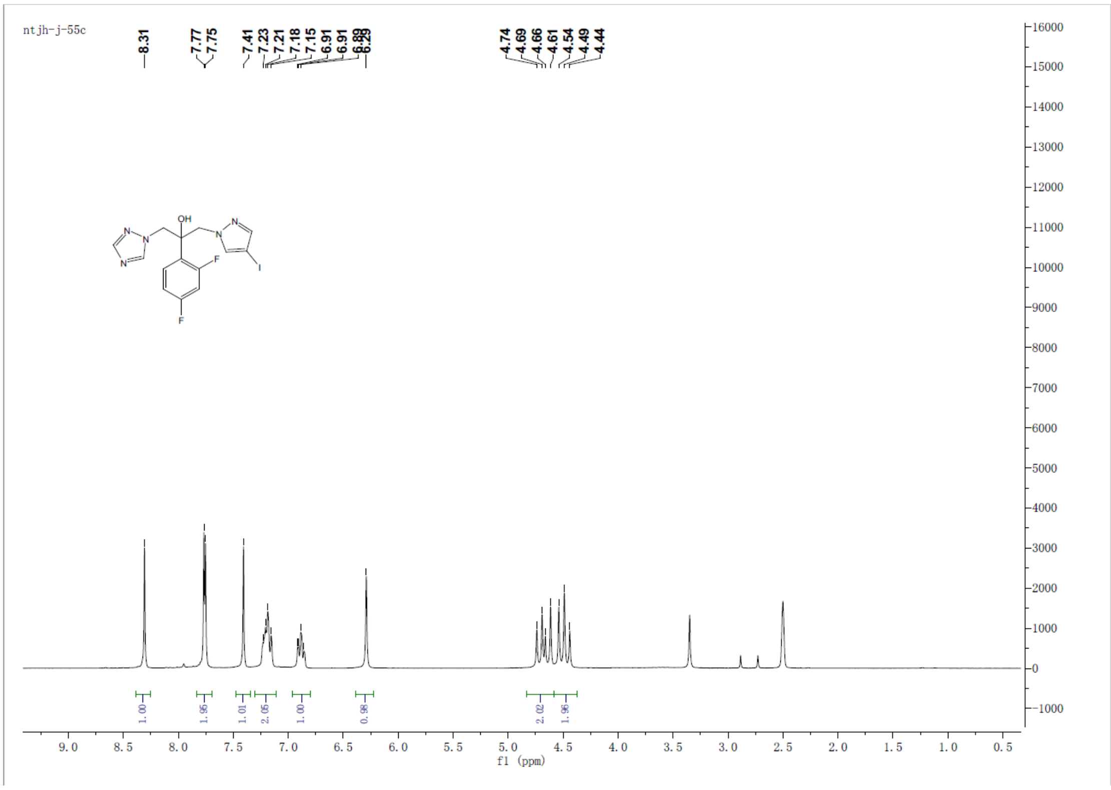

^{1}H NMR Spectrum of Compound **2** (300 MHz, DMSO-d_6)

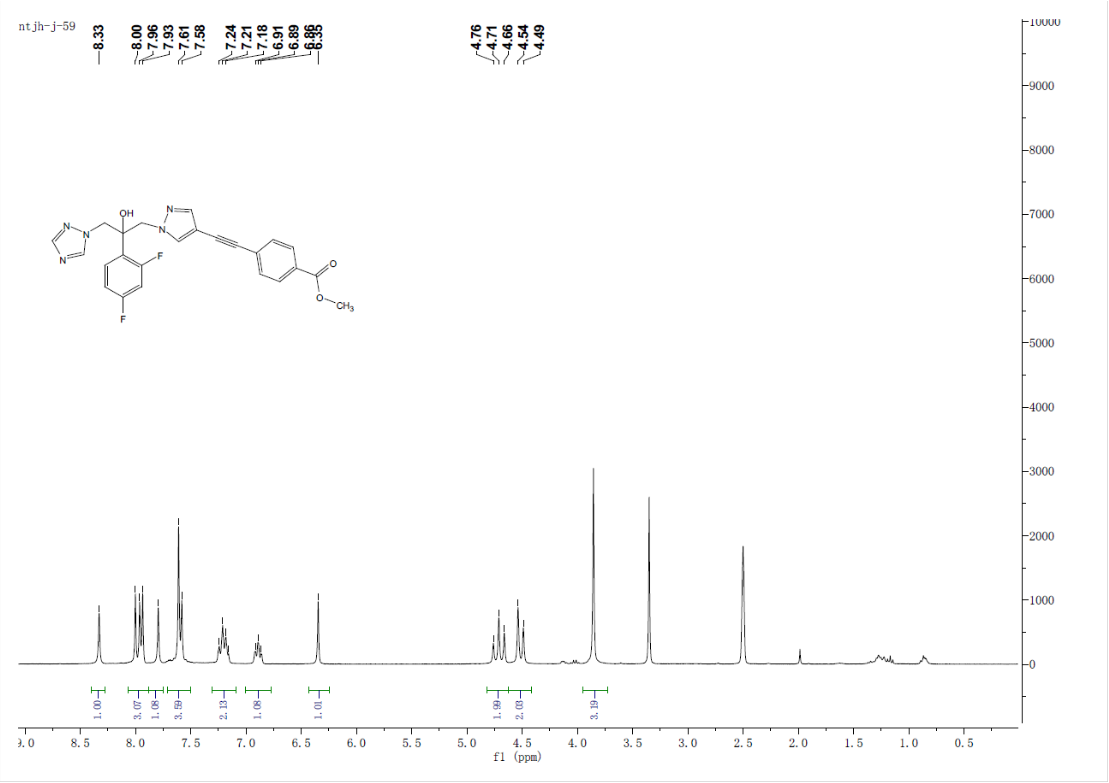

^{1}H NMR Spectrum of Compound **3** (300 MHz, DMSO-d_6)

Qualitative Analysis Report

**Data Filename**	J-59.d	**Sample Name**	
**Sample Type**	Sample	**Position**	P1-D4
**Instrument Name**	Instrument 1	**User Name**	
**Acq Method**	TEST-POS-WL.m	**Acquired Time**	9/29/2019 9:51:11 AM
**IRM Calibration Status**	Success	**DA Method**	SERUM-POS-19MIN.m
**Comment**			

Sample Group **Info.**

User Spectra

Fragmentor Voltage	Collision Energy	Ionization Mode
120	0	ESI

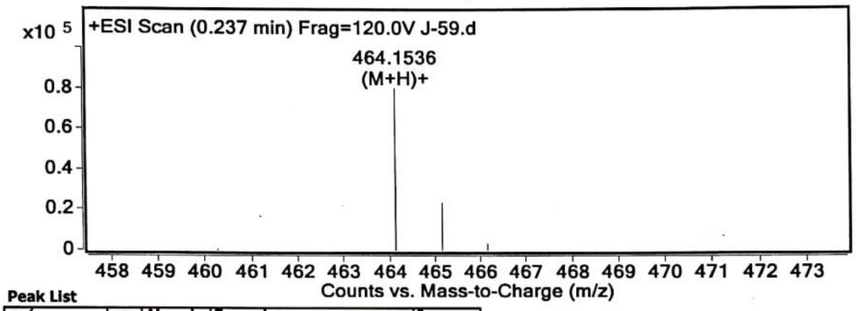

Peak List

m/z	z	Abund	Formula	Ion
121.0509		7735.4		
130.1584		8879.8		
158.1533		8234.6		
338.3418		10475.9		
437.194		4786.7		
464.1536	1	80263.8	C24 H20 F2 N5 O3	(M+H)+
465.1556	1	23644.4	C24 H20 F2 N5 O3	(M+H)+
922.0098		7589.2		

Formula Calculator Element Limits

Element	Min	Max
C	0	100
H	0	150
O	3	3
N	5	5
F	2	2

Formula Calculator Results

Formula	Best	Mass	Tgt Mass	Diff (ppm)	Ion Species	Score
C24 H19 F2 N5 O3	TRUE	463.1463	463.1456	-1.59	C24 H20 F2 N5 O3	96.86

--- End Of Report ---

HRMS Spectrum of Compound **3**

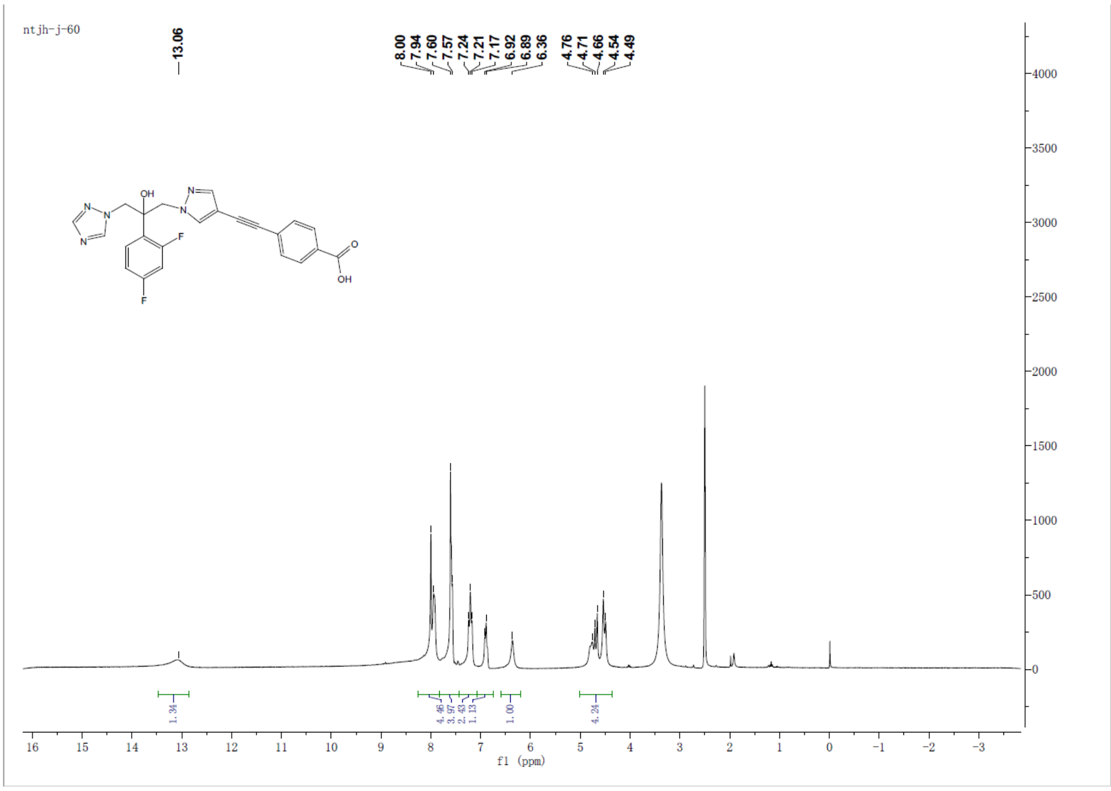

^{1}H NMR Spectrum of Compound **4** (300 MHz, DMSO-d_6)

Qualitative Analysis Report

**Data Filename**	J-60.d	**Sample Name**	
**Sample Type**	Sample	**Position**	P1-D5
**Instrument Name**	Instrument 1	**User Name**	
**Acq Method**	TEST-POS-WL.m	**Acquired Time**	9/29/2019 9:52:47 AM
**IRM Calibration Status**	Success	**DA Method**	SERUM-POS-19MIN.m
**Comment**			

Sample Group **Info.**

User Spectra

Fragmentor Voltage	Collision Energy	Ionization Mode
120	0	ESI

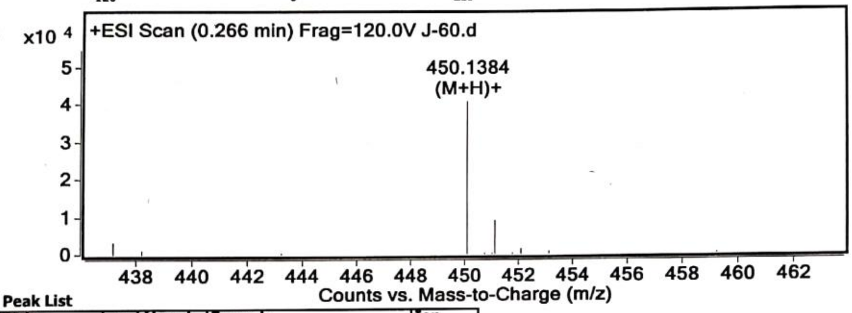

Peak List

m/z	z	Abund	Formula	Ion
121.0509		7002.9		
130.1595		6474.3		
158.1546		9415.9		
271.1874		2810.1		
338.3441		4658.9		
353.2661		3420.9		
437.1928		3237.1		
450.1384	1	41217.1	C23 H18 F2 N5 O3	(M+H)+
451.1429	1	9169.3	C23 H18 F2 N5 O3	(M+H)+
922.0098	1	8153.2		

Formula Calculator Element Limits

Element	Min	Max
C	0	100
H	0	150
O	3	3
N	5	5
F	2	2

Formula Calculator Results

Formula	Best	Mass	Tgt Mass	Diff (ppm)	Ion Species	Score
C23 H17 F2 N5 O3	TRUE	449.1311	449.1299	-2.61	C23 H18 F2 N5 O3	90.81

--- End Of Report ---

HRMS Spectrum of Compound **4**

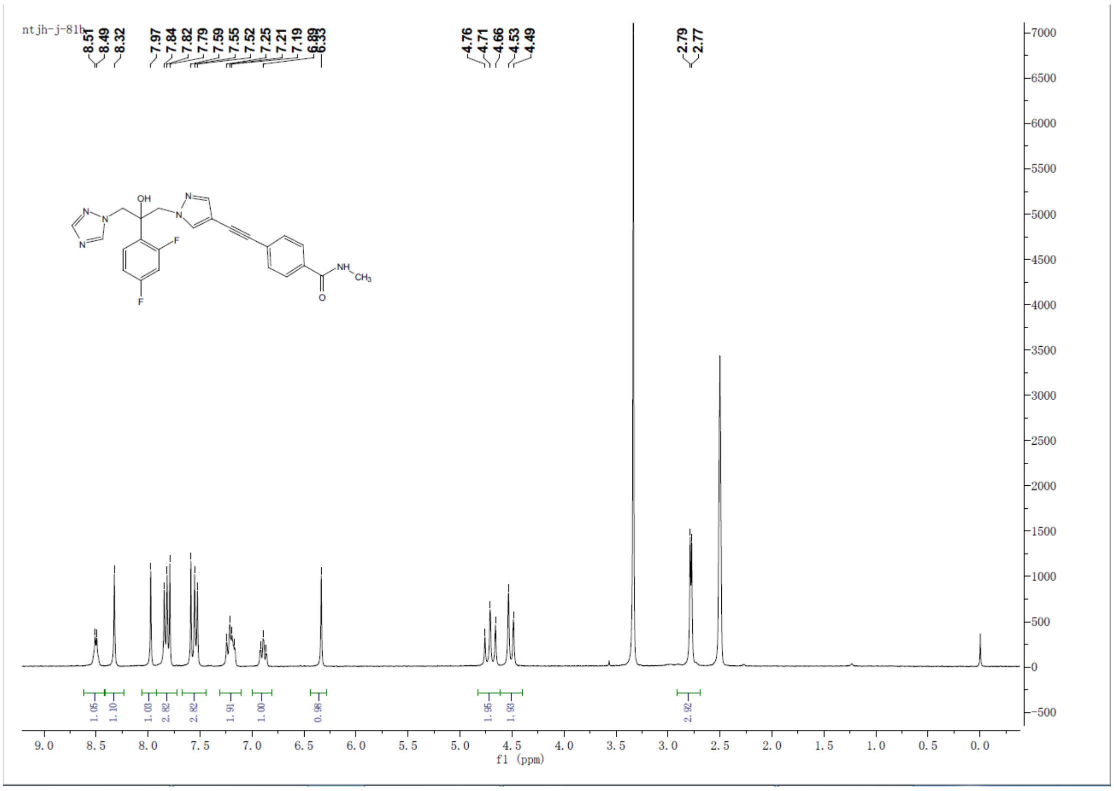

^{1}H NMR Spectrum of Compound **5a** (300 MHz, DMSO-d_6)

Qualitative Analysis Report

**Data Filename**	J-81B.d	**Sample Name**	
**Sample Type**	Sample	**Position**	P1-B6
**Instrument Name**	Instrument 1	**User Name**	
**Acq Method**	TEST-POS-WL.m	**Acquired Time**	9/29/2019 9:25:33 AM
**IRM Calibration Status**	Success	**DA Method**	SERUM-POS-19MIN.m
**Comment**			

Sample Group **Info.**

User Spectra

Fragmentor Voltage	Collision Energy	Ionization Mode
120	0	ESI

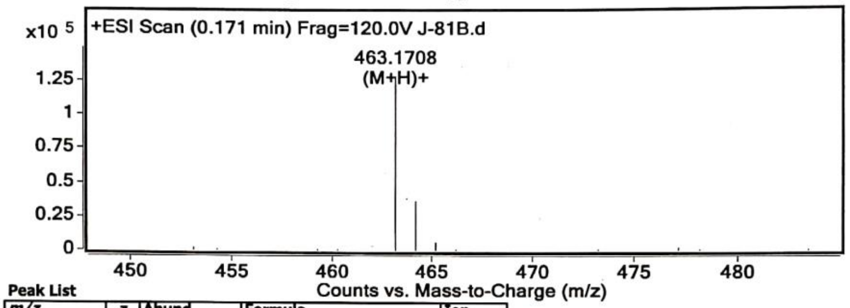

Peak List

m/z	z	Abund	Formula	Ion
121.0509		7480.8		
130.1597		12039.4		
158.1539		7665		
232.0892		13632.7		
437.1939		20767.2		
463.1708	1	129116.4	C24 H21 F2 N6 O2	(M+H)+
464.1728	1	37169.7	C24 H21 F2 N6 O2	(M+H)+
619.5299		8455.1		
647.558		7521.7		
922.0098		9212.8		

Formula Calculator Element Limits

Element	Min	Max
C	0	100
H	0	150
O	2	2
N	6	6
F	2	2

Formula Calculator Results

Formula	Best	Mass	Tgt Mass	Diff (ppm)	Ion Species	Score
C24 H20 F2 N6 O2	TRUE	462.1635	462.1616	-4.14	C24 H21 F2 N6 O2	91.61

--- End Of Report ---

HRMS Spectrum of Compound **5a**

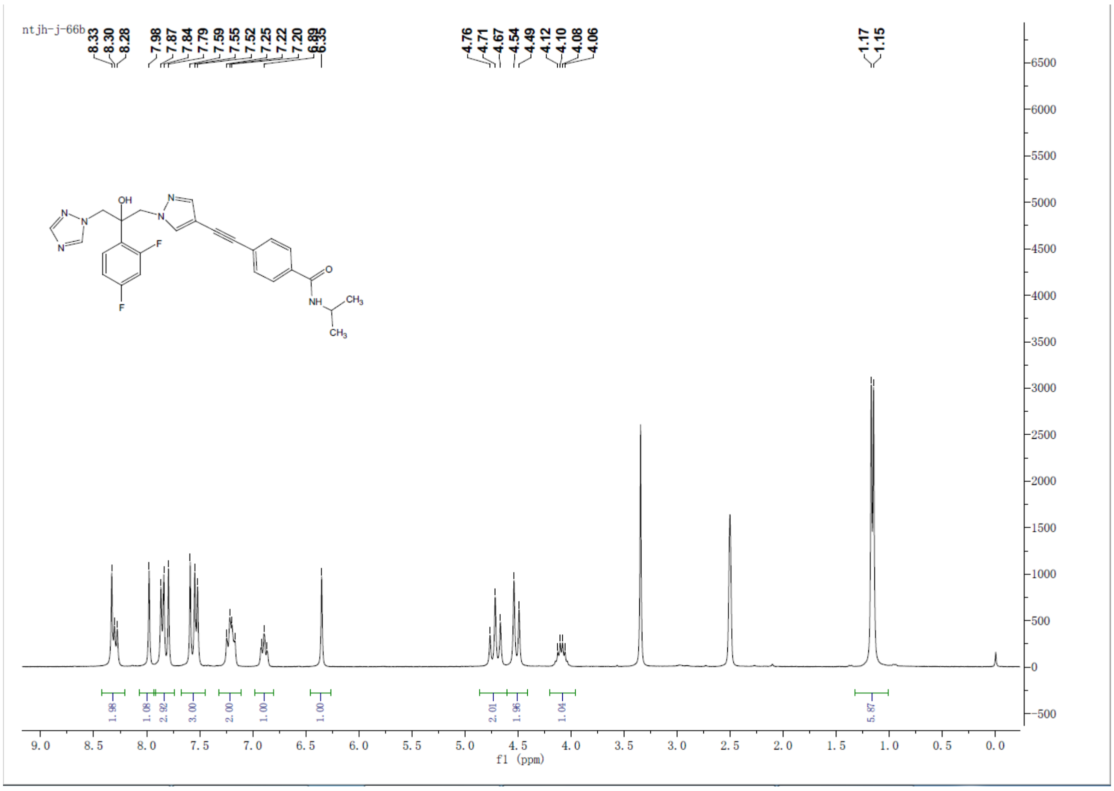

^{1}H NMR Spectrum of Compound **5b** (300 MHz, DMSO-*d6*)

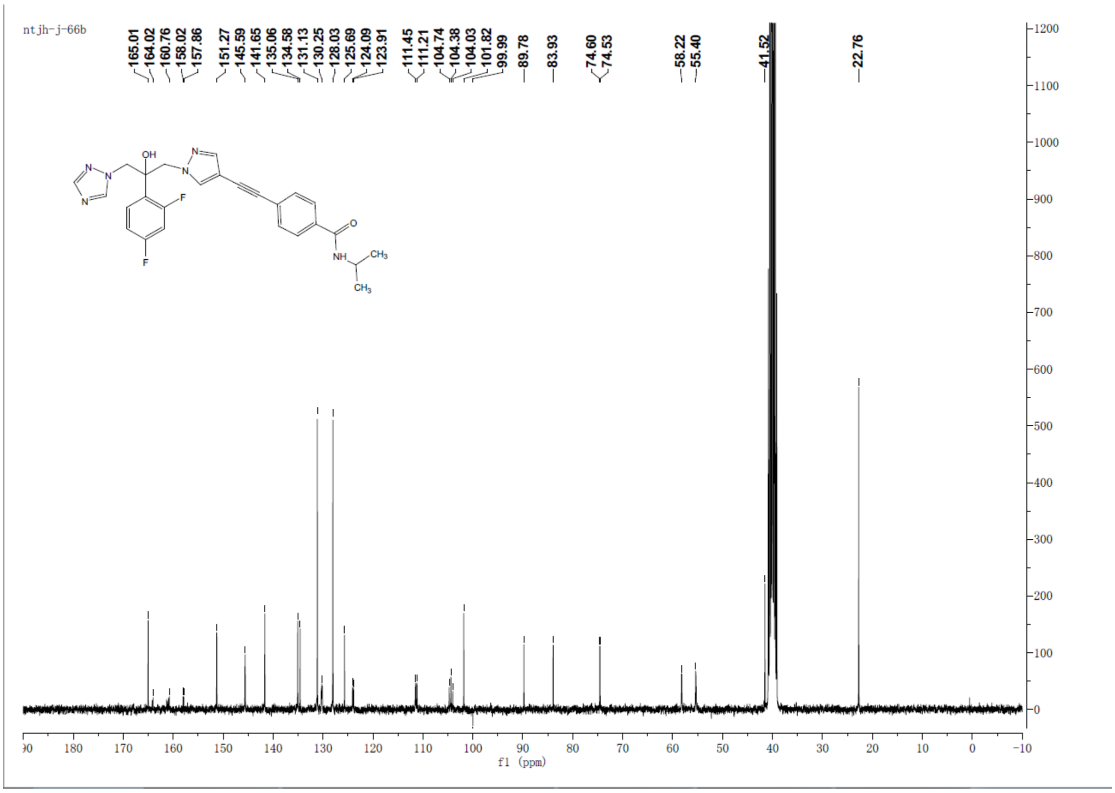

^{13}C NMR Spectrum of Compound **5b** (75 MHz, DMSO-*d6*)

Qualitative Analysis Report

**Data Filename**	J-66B.d	**Sample Name**	
**Sample Type**	Sample	**Position**	P1-A7
**Instrument Name**	Instrument 1	**User Name**	
**Acq Method**	TEST-POS-WL.m	**Acquired Time**	9/29/2019 9:12:44 AM
**IRM Calibration Status**	Success	**DA Method**	SERUM-POS-19MIN.m
**Comment**			

Sample Group **Info.**

**Data Filename**	NJ-66B.d	**Sample Name**	
**Sample Type**	Sample	**Position**	P1-A7
**Instrument Name**	Instrument 1	**User Name**	
**Acq Method**	TEST-NEG-WL.m	**Acquired Time**	9/29/2019 10:32:20 AM
**IRM Calibration Status**	Some Ions Missed	**DA Method**	SERUM-POS-19MIN.m
**Comment**			

Sample Group **Info.**

User Spectra

Fragmentor Voltage	Collision Energy	Ionization Mode
120	0	ESI

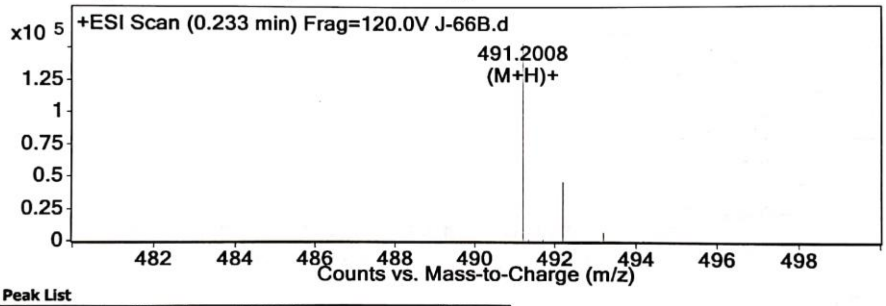

Peak List

m/z	z	Abund	Formula	Ion
130.1585		21965.3		
246.104		34370.4		
491.2008	1	154329.8	C26 H25 F2 N6 O2	(M+H)+
492.2029	1	45453.3	C26 H25 F2 N6 O2	(M+H)+

Formula Calculator Element Limits

Element	Min	Max
C	0	100
H	0	150
N	6	10
O	1	10
F	2	2

Formula Calculator Results

Formula	Best	Mass	Tgt Mass	Diff (ppm)	Ion Species	Score
C26 H24 F2 N6 O2	TRUE	490.1935	490.1929	-1.27	C26 H25 F2 N6 O2	98.22
C24 H22 F2 N9 O		490.1935	490.1915	-4.02	C24 H23 F2 N9 O	92.07

Fragmentor Voltage	Collision Energy	Ionization Mode
120	0	ESI

HRMS Spectrum of Compound **5b**

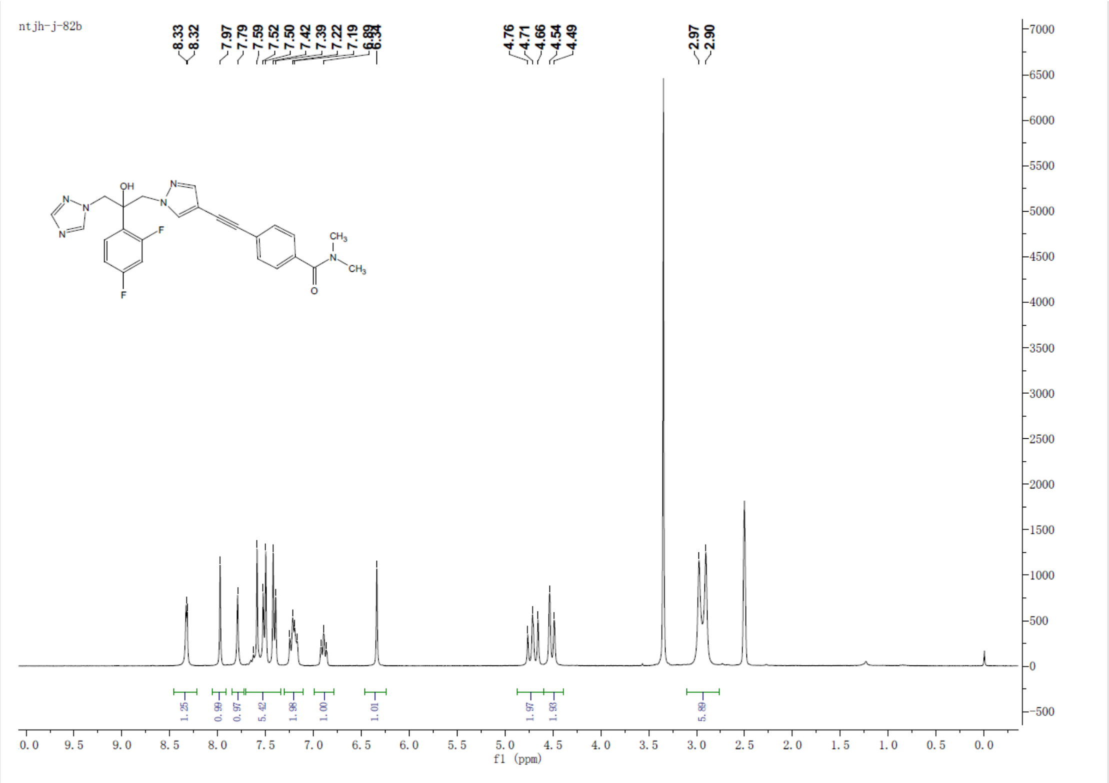

^{1}H NMR Spectrum of Compound **5c** (300 MHz, DMSO-d_6)

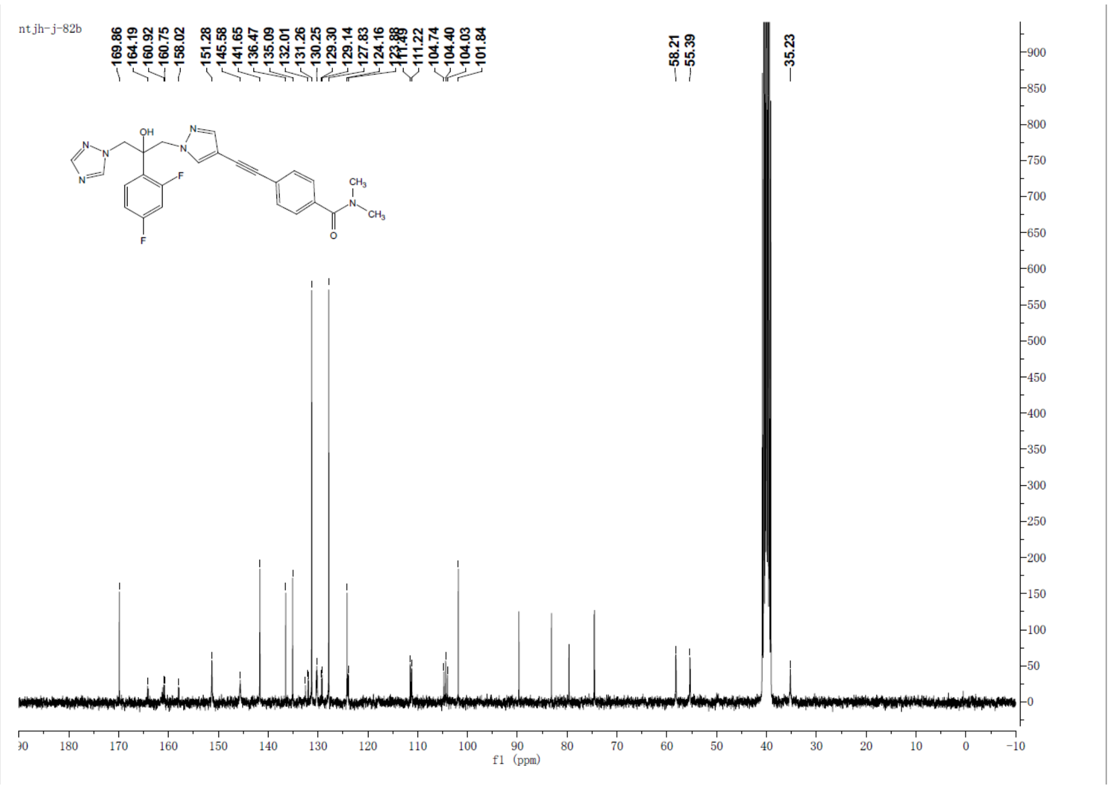

^{13}C NMR Spectrum of Compound **5c** (75 MHz, DMSO-d_6)

Qualitative Analysis Report

**Data Filename**	J-82B.d	**Sample Name**	
**Sample Type**	Sample	**Position**	P1-C3
**Instrument Name**	Instrument 1	**User Name**	
**Acq Method**	TEST-POS-WL.m	**Acquired Time**	9/29/2019 9:35:07 AM
**IRM Calibration Status**	Success	**DA Method**	SERUM-POS-19MIN.m
**Comment**			

Sample Group **Info.**

User Spectra

Fragmentor Voltage	Collision Energy	Ionization Mode
120	0	ESI

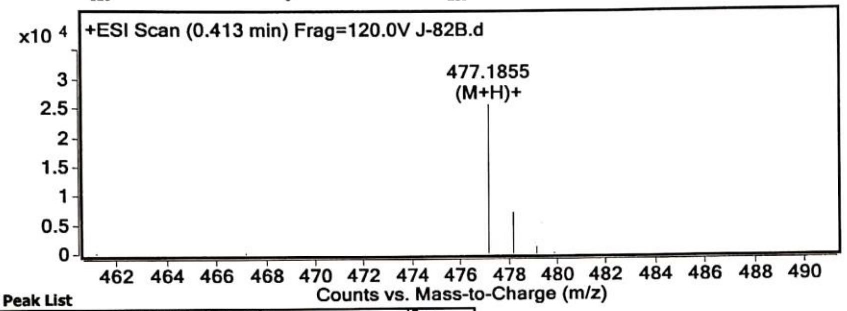

Peak List

m/z	z	Abund	Formula	Ion
121.0509		5302.3		
122.0584		2977.5		
125.9853		2904.7		
130.1588		9587.9		
158.1538	1	8614.9		
239.0961		4523		
271.187		3117.5		
477.1855	1	25667.8	C25 H23 F2 N6 O2	(M+H)+
478.1876	1	7188.1	C25 H23 F2 N6 O2	(M+H)+
922.0098		7971.4		

Formula Calculator Element Limits

Element	Min	Max
C	0	100
H	0	150
O	2	2
N	6	6
F	2	2

Formula Calculator Results

Formula	Best	Mass	Tgt Mass	Diff (ppm)	Ion Species	Score
C25 H22 F2 N6 O2	TRUE	476.1782	476.1772	-2.11	C25 H23 F2 N6 O2	96.6

--- End Of Report ---

HRMS Spectrum of Compound **5c**

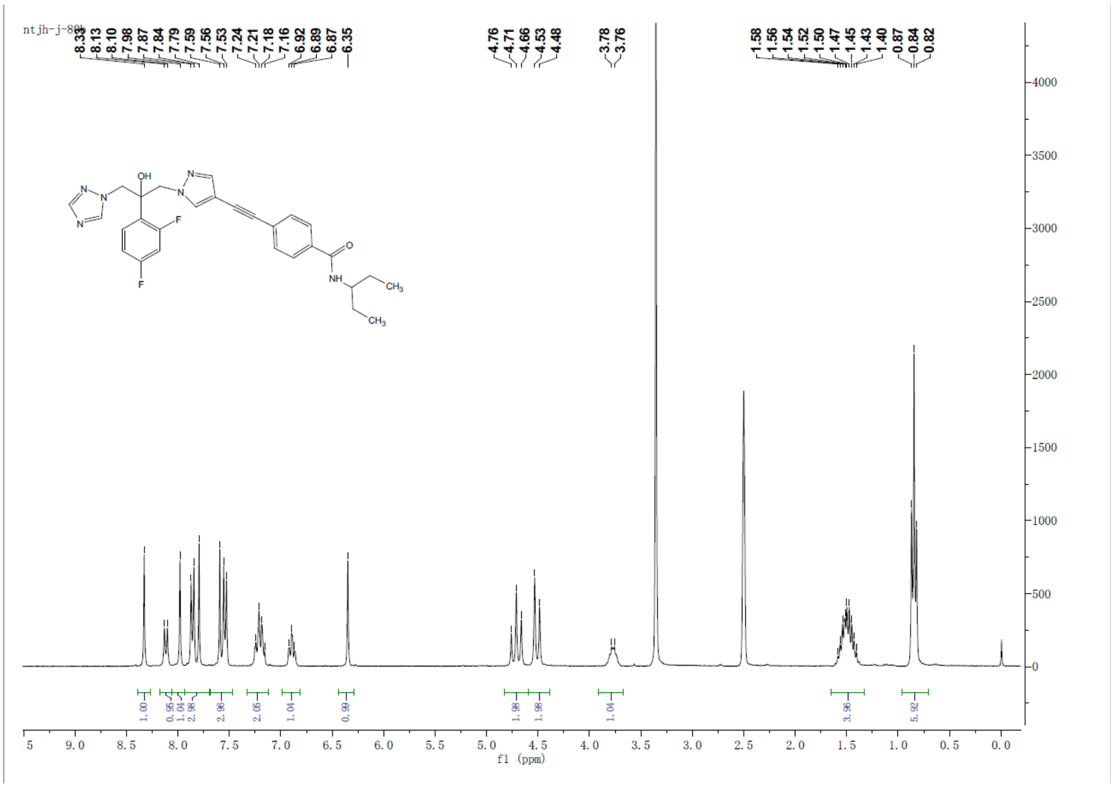

^{1}H NMR Spectrum of Compound **5d** (300 MHz, DMSO-d_6)

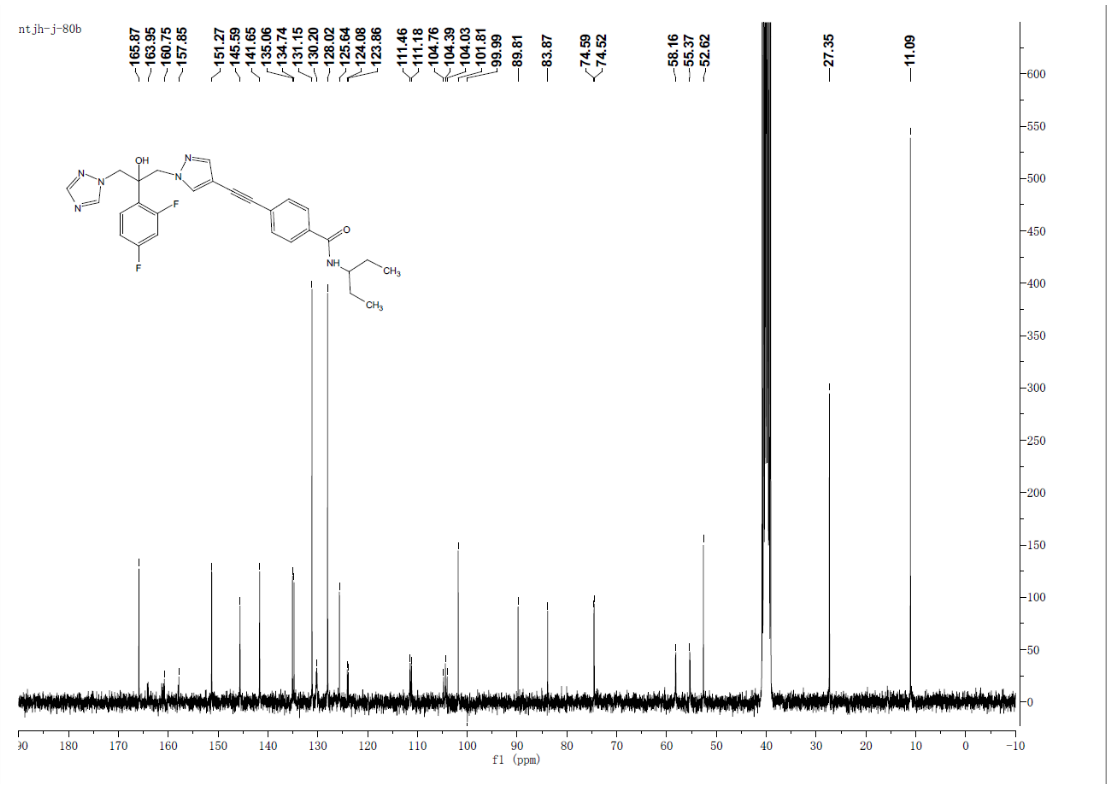

^{13}C NMR Spectrum of Compound **5d** (75 MHz, DMSO-d_6)

Qualitative Analysis Report

**Data Filename**	J-80B.d	**Sample Name**	
**Sample Type**	Sample	**Position**	P1-C8
**Instrument Name**	Instrument 1	**User Name**	
**Acq Method**	TEST-POS-WL.m	**Acquired Time**	9/29/2019 9:43:13 AM
**IRM Calibration Status**	Success	**DA Method**	SERUM-POS-19MIN.m
**Comment**			

Sample Group **Info.**

User Spectra

Fragmentor Voltage	Collision Energy	Ionization Mode
120	0	ESI

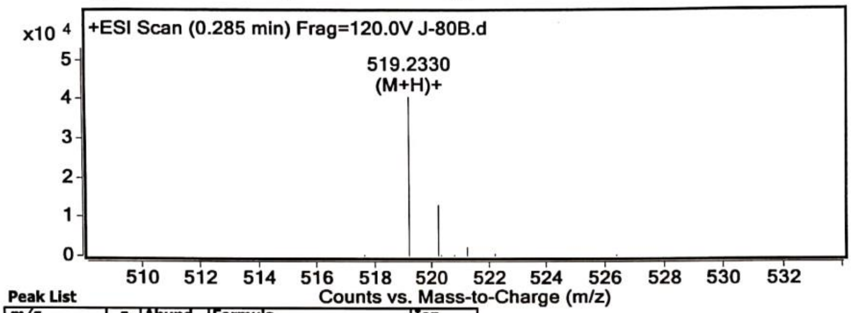

Peak List

m/z	z	Abund	Formula	Ion
121.0509		5653.9		
122.9643		3525.6		
130.1585		7884.2		
158.1543		10584.7		
260.1202	2	15800		
260.6222	2	4538.4		
302.2465		3504.4		
519.233	1	40628	C28 H29 F2 N6 O2	(M+H)+
520.2356	1	13351.9	C28 H29 F2 N6 O2	(M+H)+
922.0098	1	8767.5		

Formula Calculator Element Limits

Element	Min	Max
C	0	100
H	0	150
O	2	2
N	6	6
F	2	2

Formula Calculator Results

Formula	Best	Mass	Tgt Mass	Diff (ppm)	Ion Species	Score
C28 H28 F2 N6 O2	TRUE	518.2258	518.2242	-3.03	C28 H29 F2 N6 O2	94.8

--- End Of Report ---

HRMS Spectrum of Compound **5d**

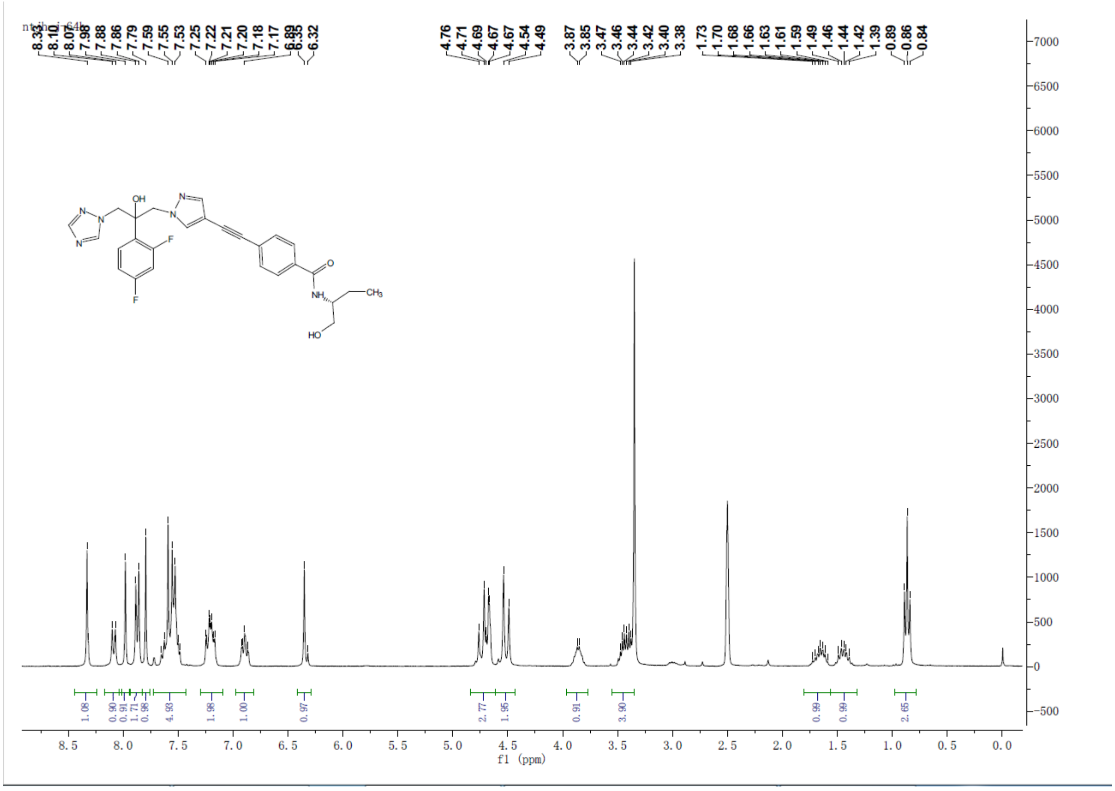

^{1}H NMR Spectrum of Compound **5e** (300 MHz, DMSO-d_6)

Qualitative Analysis Report

**Data Filename**	J-64B.d	**Sample Name**	
**Sample Type**	Sample	**Position**	P1-C5
**Instrument Name**	Instrument 1	**User Name**	
**Acq Method**	TEST-POS-WL.m	**Acquired Time**	9/29/2019 9:38:21 AM
**IRM Calibration Status**	Success	**DA Method**	SERUM-POS-19MIN.m
**Comment**			

Sample Group **Info.**

User Spectra

Fragmentor Voltage	Collision Energy	Ionization Mode
120	0	ESI

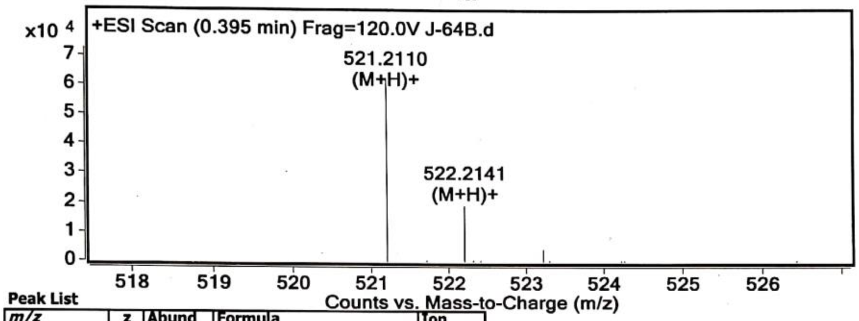

Peak List

m/z	z	Abund	Formula	Ion
130.1591		7915.7		
158.1543		7949.8		
261.111	2	29360.8		
261.6132	2	9428.7		
279.0938		8359.7		
506.156		8840.4		
521.211	1	62691.3	C27 H27 F2 N6 O3	(M+H)+
522.2141	1	19159.5	C27 H27 F2 N6 O3	(M+H)+
821.2771		5394.7		
922.0098		6387.5		

Formula Calculator Element Limits

Element	Min	Max
C	0	100
H	0	150
O	3	3
N	6	6
F	2	2

Formula Calculator Results

Formula	Best	Mass	Tgt Mass	Diff (ppm)	Ion Species	Score
C27 H26 F2 N6 O3	TRUE	520.2038	520.2034	-0.62	C27 H27 F2 N6 O3	98.42

--- End Of Report ---

HRMS Spectrum of Compound **5e**

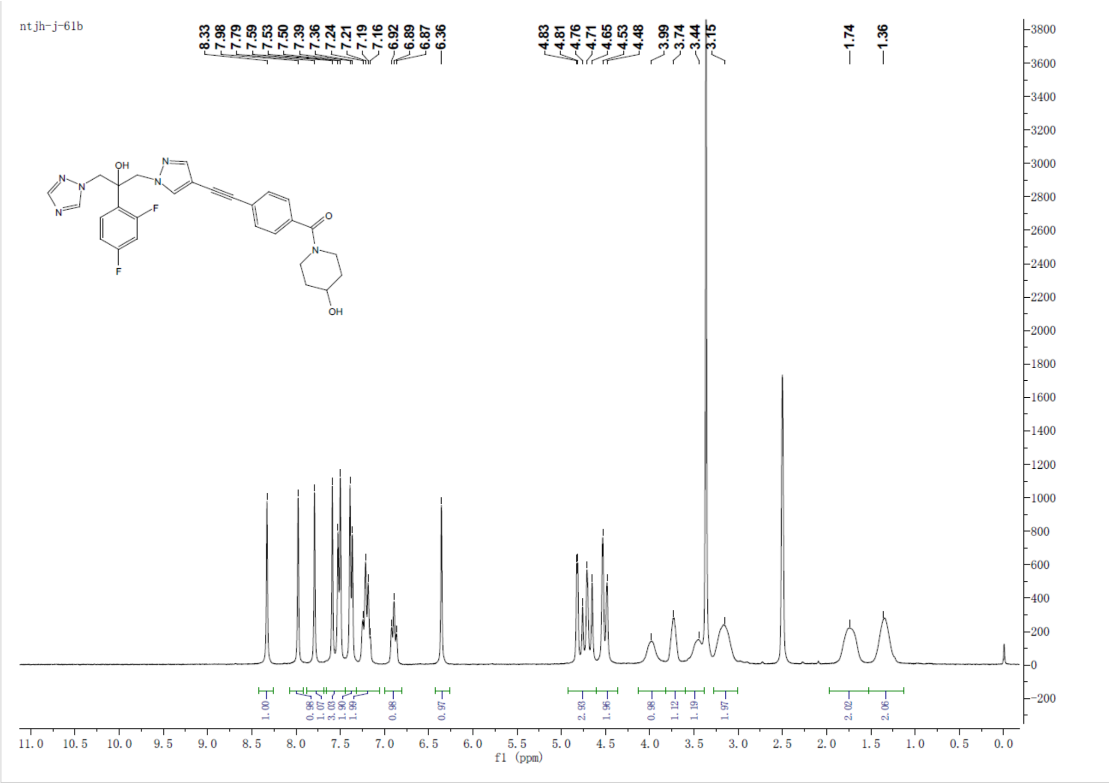

^{1}H NMR Spectrum of Compound **5f** (300 MHz, DMSO-d_6)

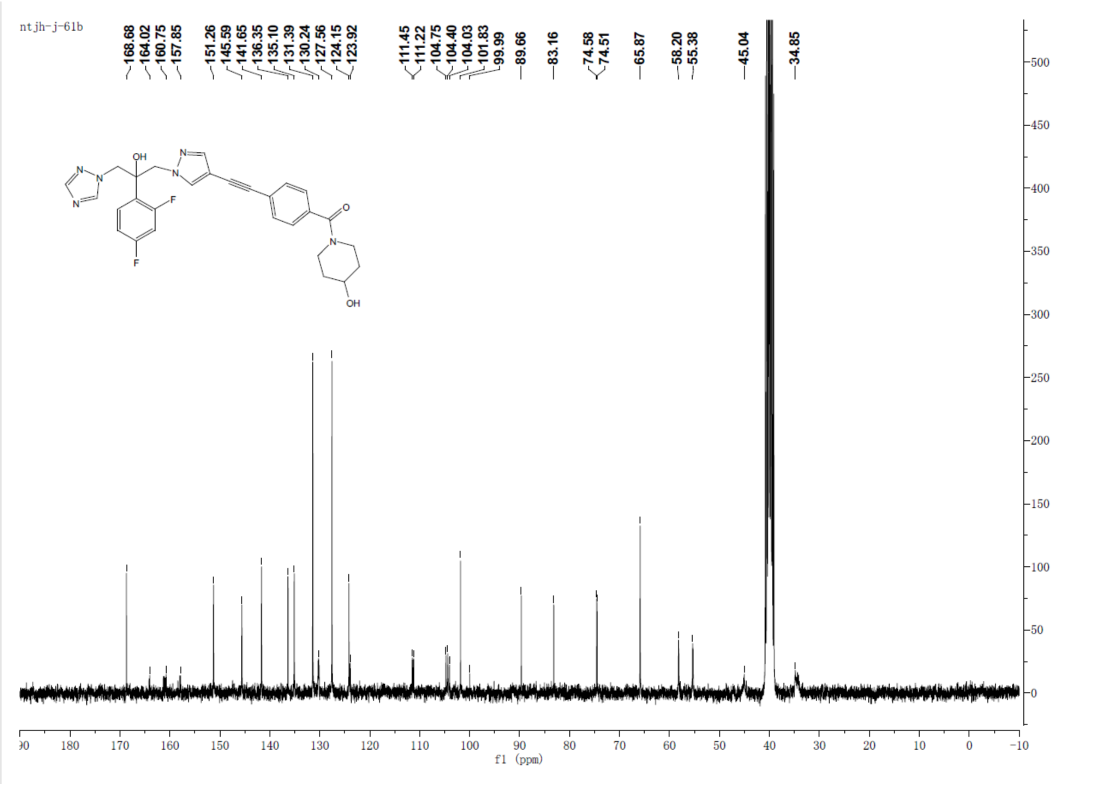

^{13}C NMR Spectrum of Compound **5f** (75 MHz, DMSO-d_6)

Qualitative Analysis Report

**Data Filename**	J-61B.d	**Sample Name**	
**Sample Type**	Sample	**Position**	P1-C7
**Instrument Name**	Instrument 1	**User Name**	
**Acq Method**	TEST-POS-WL.m	**Acquired Time**	9/29/2019 9:41:36 AM
**IRM Calibration Status**	Success	**DA Method**	SERUM-POS-19MIN.m
**Comment**			

Sample Group **Info.**

User Spectra

Fragmentor Voltage	Collision Energy	Ionization Mode
120	0	ESI

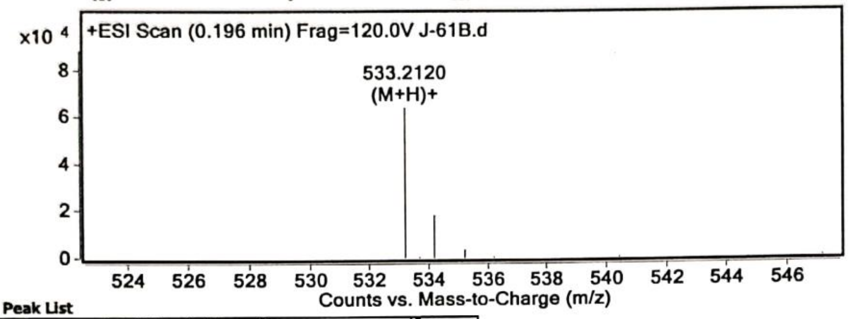

Peak List

m/z	z	Abund	Formula	Ion
121.0509		6240.5		
122.9641		5515		
158.1542		8796.8		
267.1094	2	22644.5		
267.611	2	7463.8		
338.3425		7127.2		
437.1941	1	13400.3		
533.212	1	64637	C28 H27 F2 N6 O3	(M+H)+
534.2149	1	18443	C28 H27 F2 N6 O3	(M+H)+
922.0098		7973.8		

Formula Calculator Element Limits

Element	Min	Max
C	0	100
H	0	150
O	3	3
N	6	6
F	2	2

Formula Calculator Results

Formula	Best	Mass	Tgt Mass	Diff (ppm)	Ion Species	Score
C28 H26 F2 N6 O3	TRUE	532.2047	532.2034	-2.37	C28 H27 F2 N6 O3	94.1

--- End Of Report ---

HRMS Spectrum of Compound **5f**

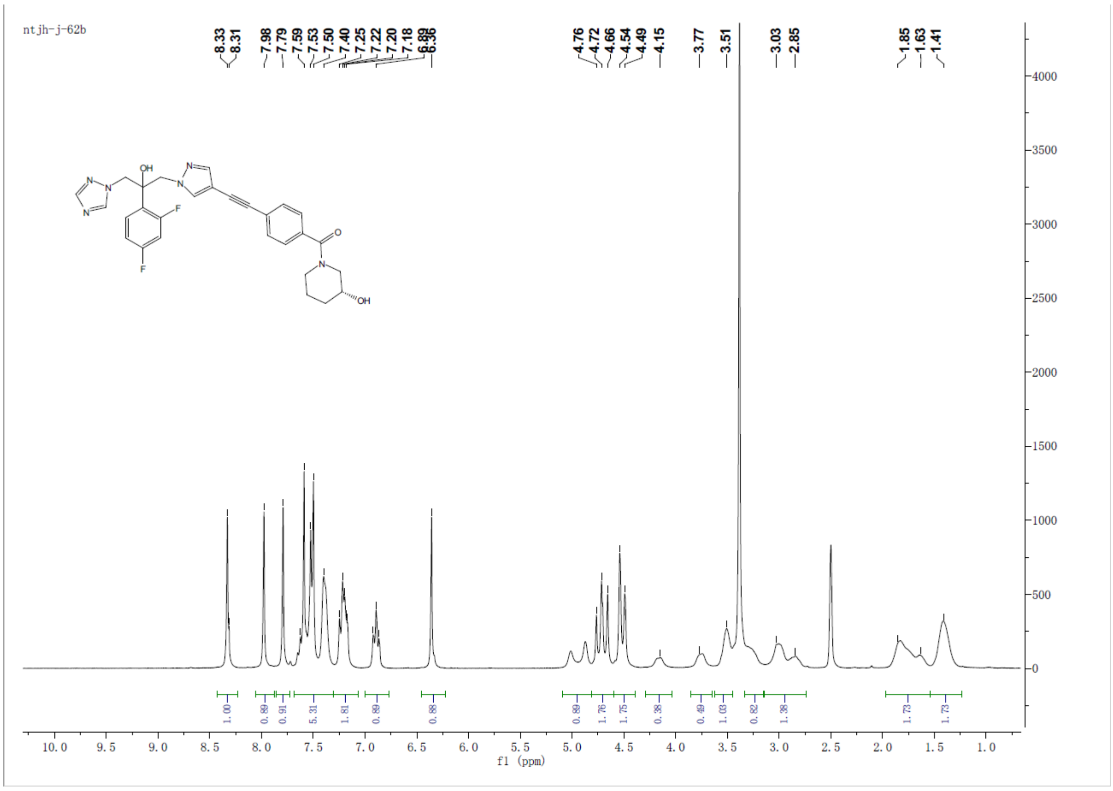

^{1}H NMR Spectrum of Compound **5g** (300 MHz, DMSO-d_6)

Qualitative Analysis Report

**Data Filename**	J-62B.d	**Sample Name**	
**Sample Type**	Sample	**Position**	P1-B9
**Instrument Name**	Instrument 1	**User Name**	
**Acq Method**	TEST-POS-WL.m	**Acquired Time**	9/29/2019 9:30:16 AM
**IRM Calibration Status**	Success	**DA Method**	SERUM-POS-19MIN.m
**Comment**			

Sample Group **Info.**

User Spectra

Fragmentor Voltage	Collision Energy	Ionization Mode
120	0	ESI

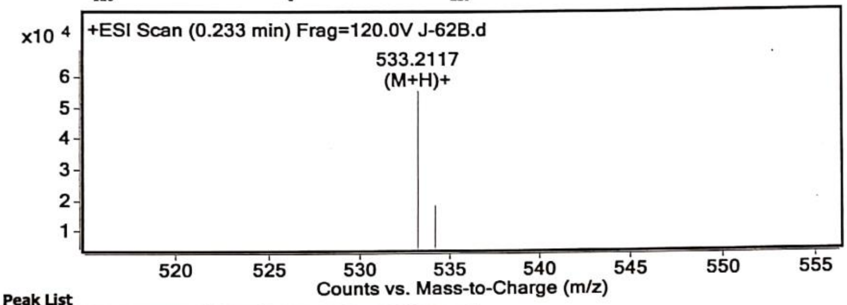

Peak List

m/z	z	Abund	Formula	Ion
121.0509		5704.9		
122.9637		5512.3		
130.1586		9390.3		
158.1555		9155.3		
267.1108	2	21362.7		
267.6098	2	6457		
279.0954		6730.3		
533.2117	1	55532.2	C28 H27 F2 N6 O3	(M+H)+
534.215	1	18532.9	C28 H27 F2 N6 O3	(M+H)+
922.0098		8725.2		

Formula Calculator Element Limits

Element	Min	Max
C	0	100
H	0	150
O	3	3
N	6	6
F	2	2

Formula Calculator Results

Formula	Best	Mass	Tgt Mass	Diff (ppm)	Ion Species	Score
C28 H26 F2 N6 O3	TRUE	532.2044	532.2034	-1.78	C28 H27 F2 N6 O3	97.87

--- End Of Report ---

HRMS Spectrum of Compound **5g**

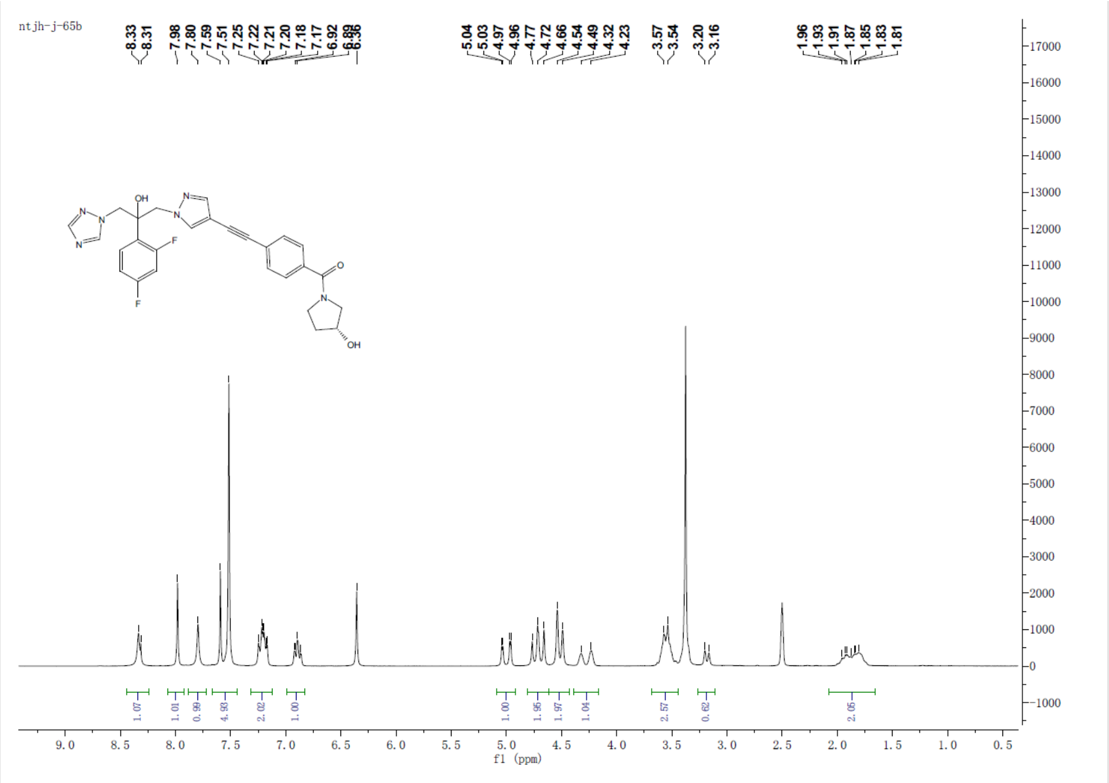

^{1}H NMR Spectrum of Compound **5h** (300 MHz, DMSO-d_6)

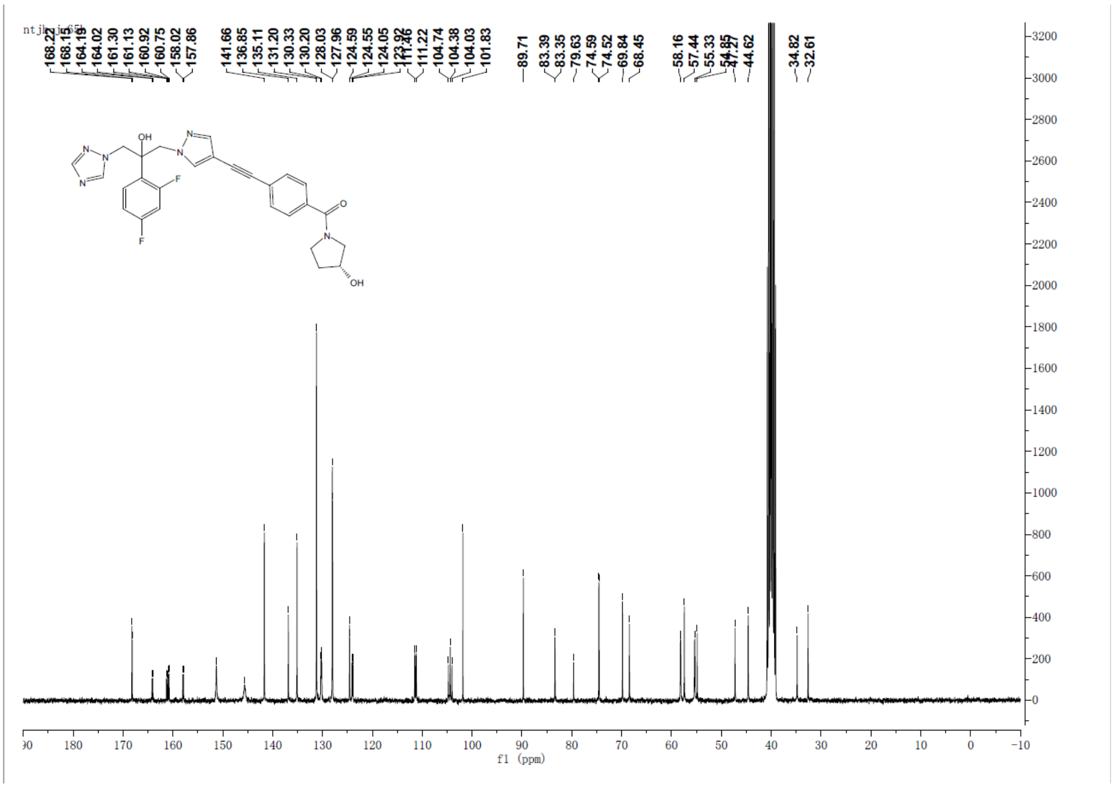

^{13}C NMR Spectrum of Compound **5h** (75 MHz, DMSO-d_6)

Qualitative Analysis Report

**Data Filename**	J-65B.d	**Sample Name**	
**Sample Type**	Sample	**Position**	P1-B2
**Instrument Name**	Instrument 1	**User Name**	
**Acq Method**	TEST-POS-WL.m	**Acquired Time**	9/29/2019 9:19:10 AM
**IRM Calibration Status**	Success	**DA Method**	SERUM-POS-19MIN.m
**Comment**			

Sample Group **Info.**

User Spectra

Fragmentor Voltage	Collision Energy	Ionization Mode
120	0	ESI

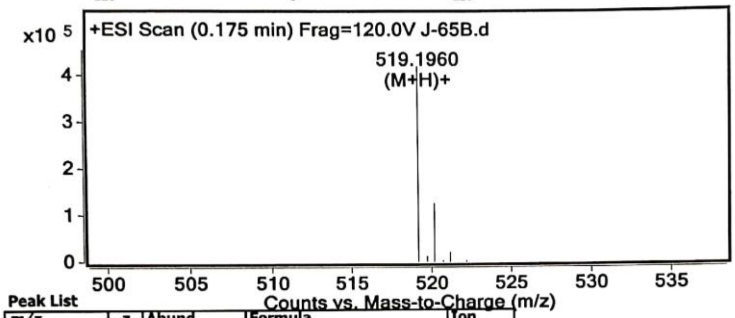

Peak List

m/z	z	Abund	Formula	Ion
260.1022	2	179764.1		
260.6029	2	57299.9		
519.196	1	421415.5	C27 H25 F2 N6 O3	(M+H)+
520.1983	1	125578.3	C27 H25 F2 N6 O3	(M+H)+
521.1998	1	21776.7	C27 H25 F2 N6 O3	(M+H)+
1059.3605		31745.6		

Formula Calculator Element Limits

Element	Min	Max
C	0	100
H	0	150
O	3	3
N	6	6
F	2	2

Formula Calculator Results

Formula	Best	Mass	Tgt Mass	Diff (ppm)	Ion Species	Score
C27 H24 F2 N6 O3	TRUE	518.1887	518.1878	-1.79	C27 H25 F2 N6 O3	97.06

--- End Of Report ---

HRMS Spectrum of Compound **5h**

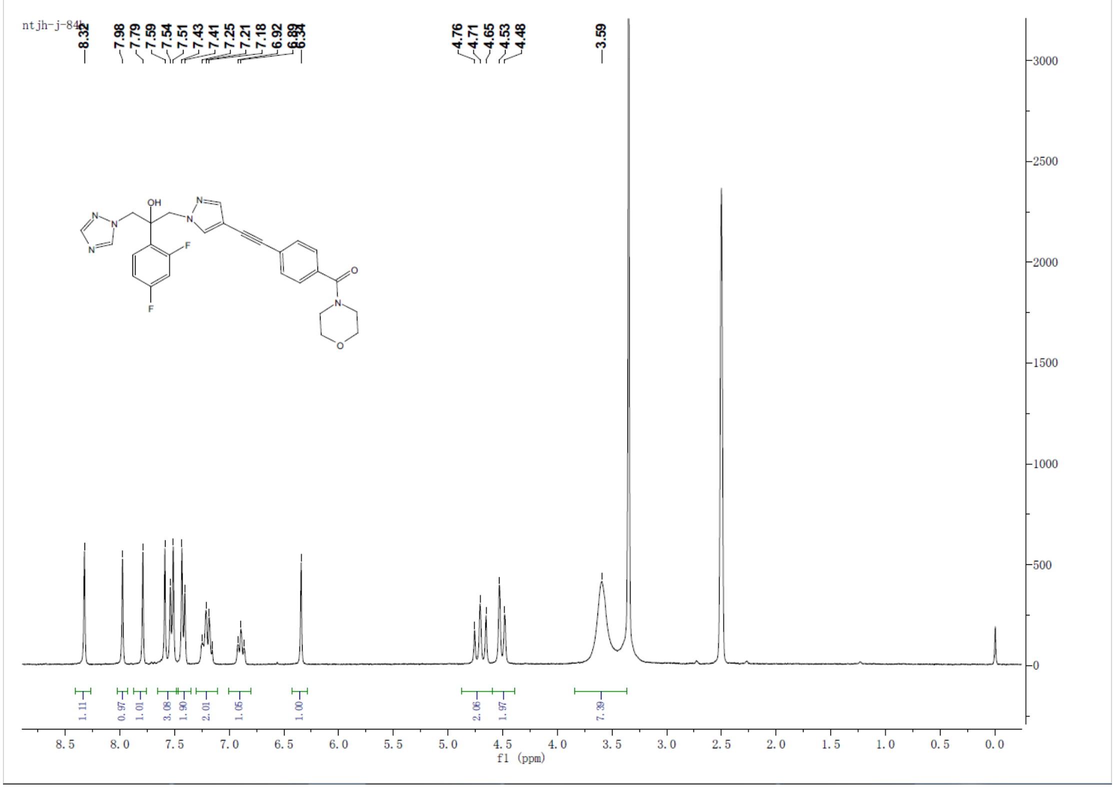

^{1}H NMR Spectrum of Compound **5i** (300 MHz, DMSO-*d*$_6$)

Qualitative Analysis Report

**Data Filename**	J-84B.d	**Sample Name**	
**Sample Type**	Sample	**Position**	P1-D2
**Instrument Name**	Instrument 1	**User Name**	
**Acq Method**	TEST-POS-WL.m	**Acquired Time**	9/29/2019 9:48:04 AM
**IRM Calibration Status**	Success	**DA Method**	SERUM-POS-19MIN.m
**Comment**			

Sample Group **Info.**

User Spectra

Fragmentor Voltage	Collision Energy	Ionization Mode
120	0	ESI

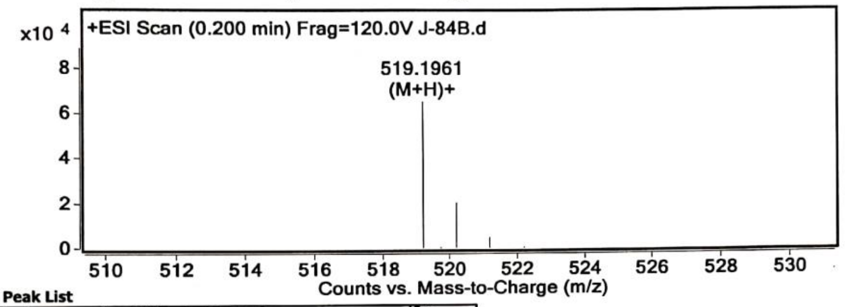

Peak List

m/z	z	Abund	Formula	Ion
121.0509		5883.7		
122.9638		5991.4		
130.159		7717.1		
141.958		6068.2		
158.1539		11107.9		
437.193		11247.5		
519.1961	1	65428.3	C27 H25 F2 N6 O3	(M+H)+
520.1983	1	20469.4	C27 H25 F2 N6 O3	(M+H)+
521.2023	1	5004.7	C27 H25 F2 N6 O3	(M+H)+
922.0098		9265.4		

Formula Calculator Element Limits

Element	Min	Max
C	0	100
H	0	150
O	3	3
N	6	6
F	2	2

Formula Calculator Results

Formula	Best	Mass	Tgt Mass	Diff (ppm)	Ion Species	Score
C27 H24 F2 N6 O3	TRUE	518.1888	518.1878	-1.95	C27 H25 F2 N6 O3	96.13

--- End Of Report ---

HRMS Spectrum of Compound **5i**

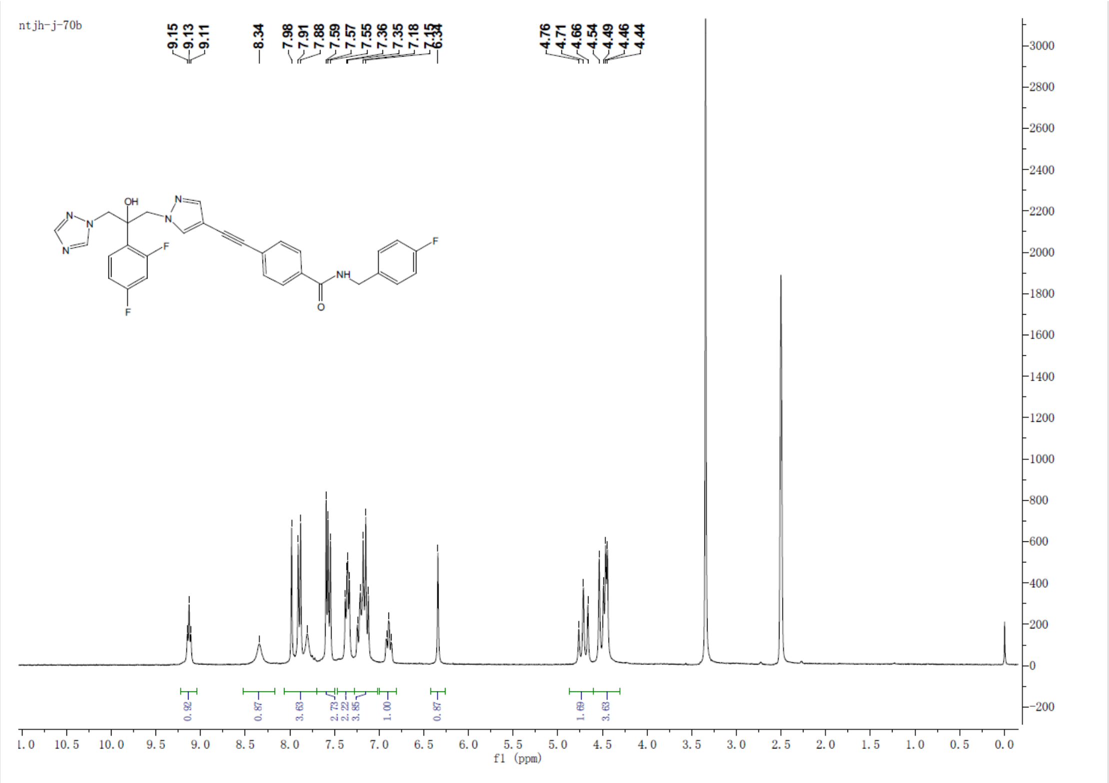

^{1}H NMR Spectrum of Compound **5j** (300 MHz, DMSO-d_6)

Qualitative Analysis Report

**Data Filename**	J-70B.d	**Sample Name**	
**Sample Type**	Sample	**Position**	P1-C1
**Instrument Name**	Instrument 1	**User Name**	
**Acq Method**	TEST-POS-WL.m	**Acquired Time**	9/29/2019 9:31:53 AM
**IRM Calibration Status**	Success	**DA Method**	SERUM-POS-19MIN.m
**Comment**			

Sample Group **Info.**

User Spectra

Fragmentor Voltage	Collision Energy	Ionization Mode
120	0	ESI

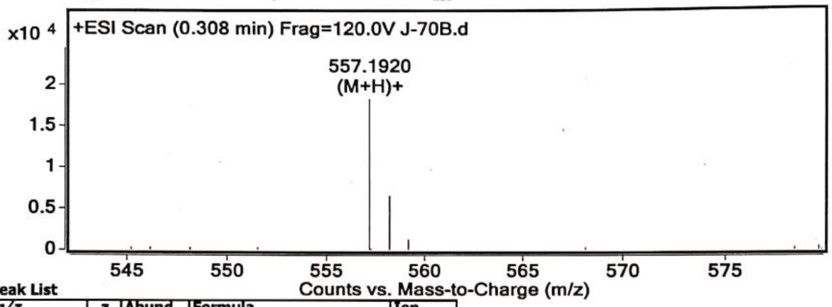

Peak List

m/z	z	Abund	Formula	Ion
121.0509		7045.9		
122.0561		6143.5		
125.9868		3432.6		
130.1589		8559		
158.0025		2998.8		
158.1543	1	10970.5		
271.189		4006.5		
557.192	1	18505.1	C30 H24 F3 N6 O2	(M+H)+
558.1917	1	6658.4	C30 H24 F3 N6 O2	(M+H)+
922.0098	1	9304.9		

Formula Calculator Element Limits

Element	Min	Max
C	0	100
H	0	150
O	2	2
N	6	6
F	3	3

Formula Calculator Results

Formula	Best	Mass	Tgt Mass	Diff (ppm)	Ion Species	Score
C30 H23 F3 N6 O2	TRUE	556.1846	556.1835	-2.06	C30 H24 F3 N6 O2	91.82

--- End Of Report ---

HRMS Spectrum of Compound **5j**

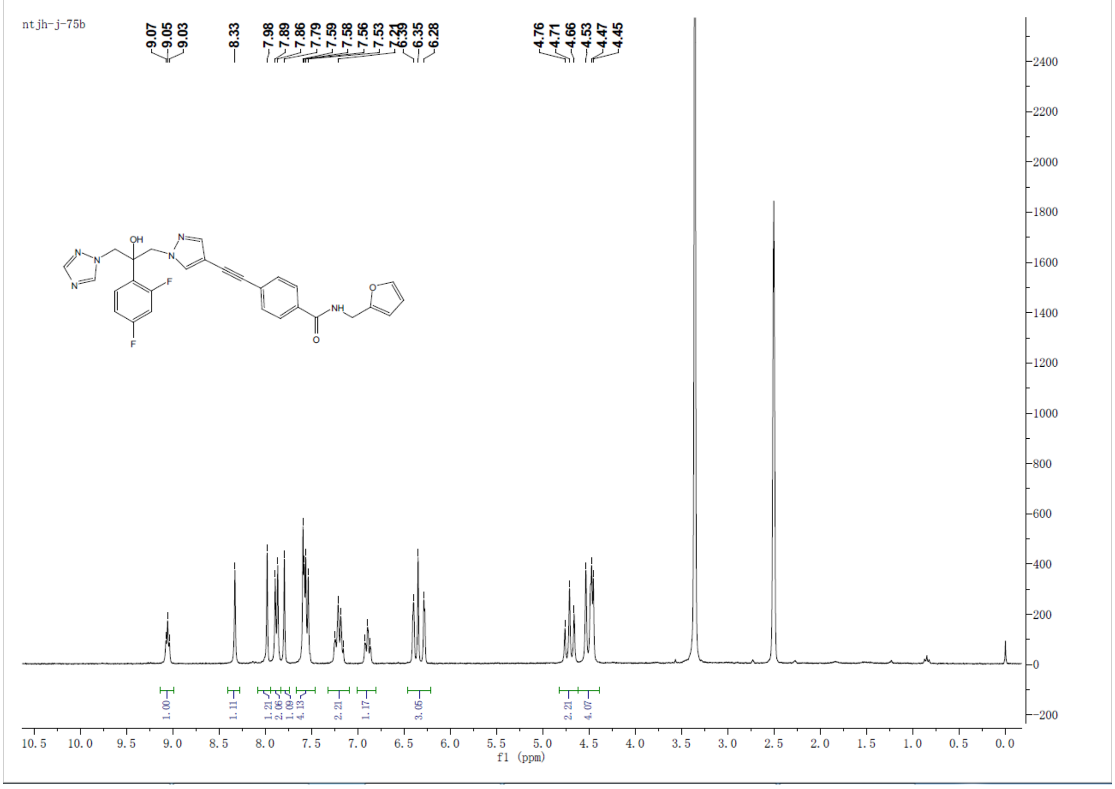

^{1}H NMR Spectrum of Compound **5k** (300 MHz, DMSO-d_6)

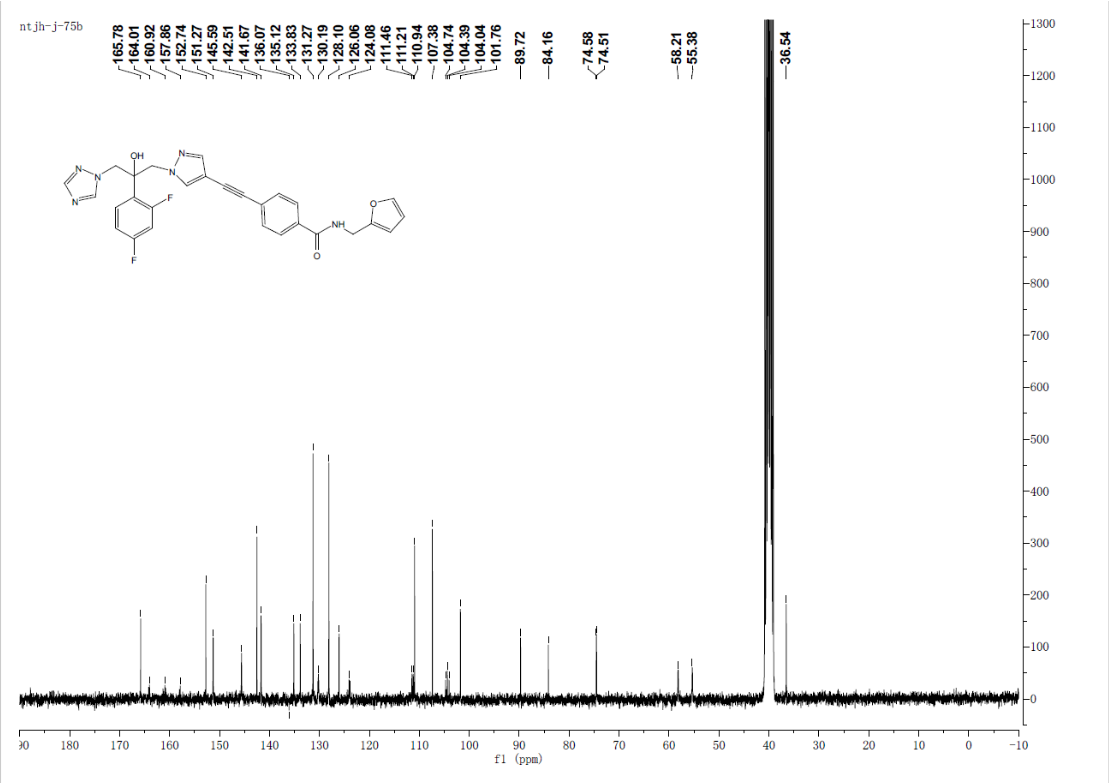

^{13}C NMR Spectrum of Compound **5k** (75 MHz, DMSO-d_6)

Qualitative Analysis Report

**Data Filename**	J-75B.d	**Sample Name**	
**Sample Type**	Sample	**Position**	P1-A6
**Instrument Name**	Instrument 1	**User Name**	
**Acq Method**	TEST-POS-WL.m	**Acquired Time**	9/29/2019 9:11:06 AM
**IRM Calibration Status**	Success	**DA Method**	SERUM-POS-19MIN.m
**Comment**			

Sample Group **Info.**

User Spectra

Fragmentor Voltage	Collision Energy	Ionization Mode
120	0	ESI

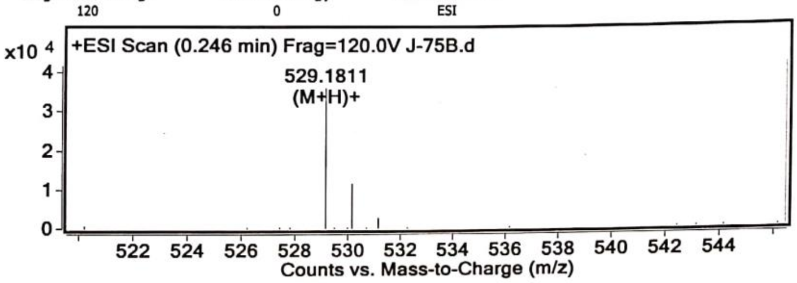

Peak List

m/z	z	Abund	Formula	Ion
121.0509		15386.7		
122.0568		7188.9		
125.9867		5835		
130.159	1	23782		
158.1544		18008.9		
302.2485	1	11403.6		
437.1932	1	7501.3		
529.1811	1	36040.5	C28 H23 F2 N6 O3	(M+H)+
530.184	1	11840.2	C28 H23 F2 N6 O3	(M+H)+
922.0098	1	19000.4		

Formula Calculator Element Limits

Element	Min	Max
C	0	100
H	0	150
O	3	3
N	6	6
F	2	2

Formula Calculator Results

Formula	Best	Mass	Tgt Mass	Diff (ppm)	Ion Species	Score
C28 H22 F2 N6 O3	TRUE	528.1738	528.1721	-3.18	C28 H23 F2 N6 O3	93.44

--- End Of Report ---

HRMS Spectrum of Compound **5k**

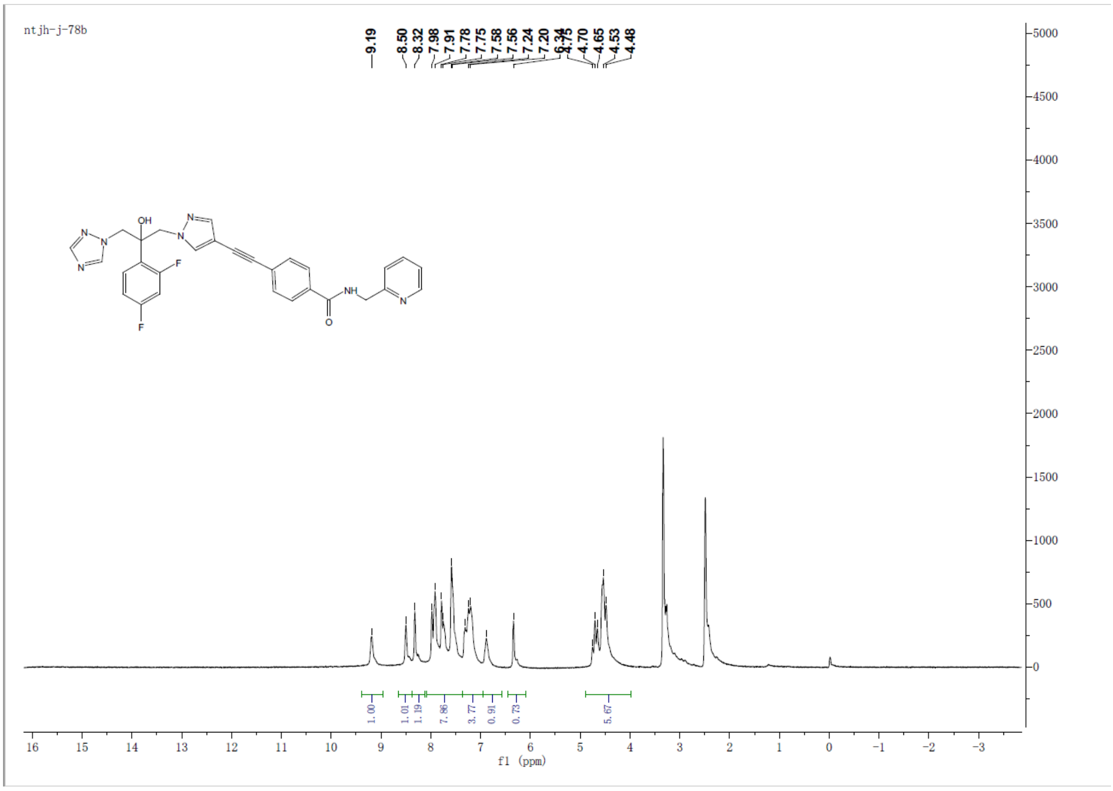

^{1}H NMR Spectrum of Compound **5l** (300 MHz, DMSO-d_6)

Qualitative Analysis Report

**Data Filename**	J-78B.d	**Sample Name**	
**Sample Type**	Sample	**Position**	P1-B4
**Instrument Name**	Instrument 1	**User Name**	
**Acq Method**	TEST-POS-WL.m	**Acquired Time**	9/29/2019 9:22:22 AM
**IRM Calibration Status**	Success	**DA Method**	SERUM-POS-19MIN.m
**Comment**			

Sample Group **Info.**

User Spectra

Fragmentor Voltage	Collision Energy	Ionization Mode
120	0	ESI

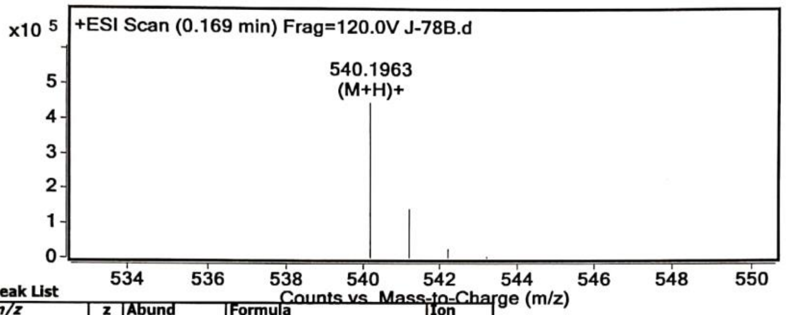

Peak List

m/z	z	Abund	Formula	Ion
236.0861		102576.9		
270.6035	2	1428221.9		
271.1046	2	442537.7		
271.6053	2	75065.6		
540.1963	1	451869.5	C29 H24 F2 N7 O2	(M+H)+
541.1989	1	145460.5	C29 H24 F2 N7 O2	(M+H)+
1101.3626	1	130216.3		
1102.3641	1	86133.2		

Formula Calculator Element Limits

Element	Min	Max
C	0	100
H	0	150
O	2	2
N	7	7
F	2	2

Formula Calculator Results

Formula	Best	Mass	Tgt Mass	Diff (ppm)	Ion Species	Score
C29 H23 F2 N7 O2	TRUE	539.189	539.1881	-1.61	C29 H24 F2 N7 O2	97.21

--- End Of Report ---

HRMS Spectrum of Compound **5l**

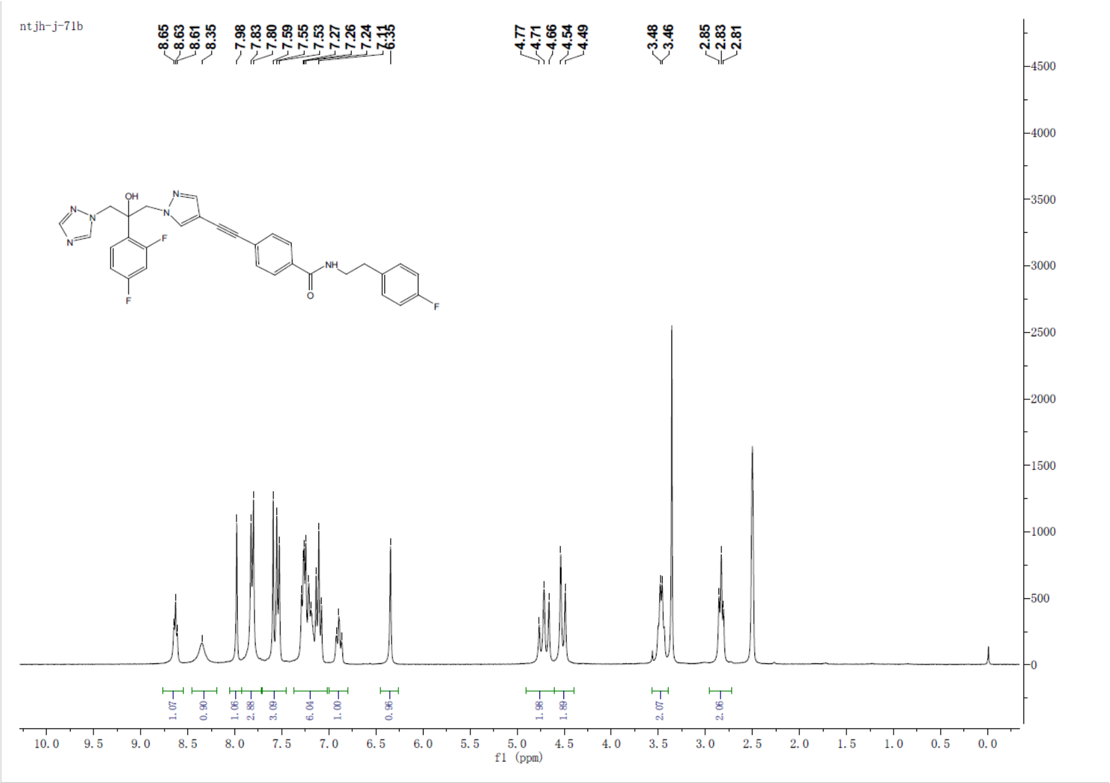

^{1}H NMR Spectrum of Compound **5m** (300 MHz, DMSO-d_6)

Qualitative Analysis Report

**Data Filename**	J-71B.d	**Sample Name**	
**Sample Type**	Sample	**Position**	P1-A5
**Instrument Name**	Instrument 1	**User Name**	
**Acq Method**	TEST-POS-WL.m	**Acquired Time**	9/29/2019 9:09:29 AM
**IRM Calibration Status**	Success	**DA Method**	SERUM-POS-19MIN.m
**Comment**			

Sample Group **Info.**

User Spectra

Fragmentor Voltage	Collision Energy	Ionization Mode
120	0	ESI

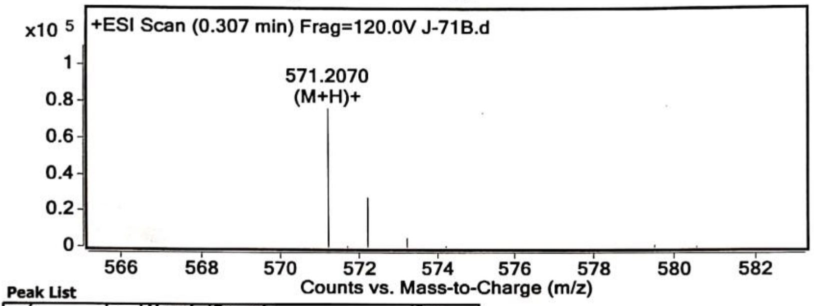

Peak List

m/z	z	Abund	Formula	Ion
121.0509		15115.3		
122.0564		5480.7		
130.1592		20854.3		
158.1533		15108.8		
286.1075	2	13789.6		
286.6088	2	6184.3		
302.2476		13078.7		
571.207	1	76809.6	C31 H26 F3 N6 O2	(M+H)+
572.2096	1	27514	C31 H26 F3 N6 O2	(M+H)+
922.0098	1	15495.3		

Formula Calculator Element Limits

Element	Min	Max
C	0	100
H	0	150
O	2	2
N	6	6
F	3	3

Formula Calculator Results

Formula	Best	Mass	Tgt Mass	Diff (ppm)	Ion Species	Score
C31 H25 F3 N6 O2	TRUE	570.1997	570.1991	-1.1	C31 H26 F3 N6 O2	99.13

--- End Of Report ---

HRMS Spectrum of Compound **5m**

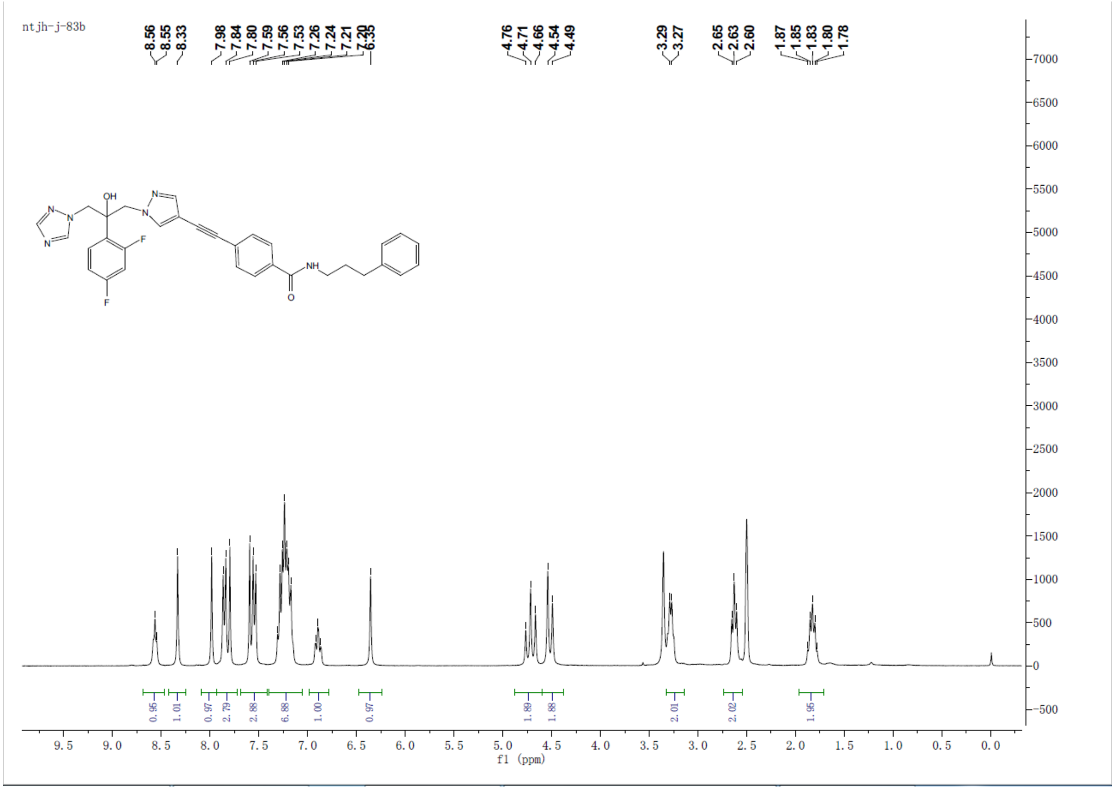

^{1}H NMR Spectrum of Compound **5n** (300 MHz, DMSO-*d6*)

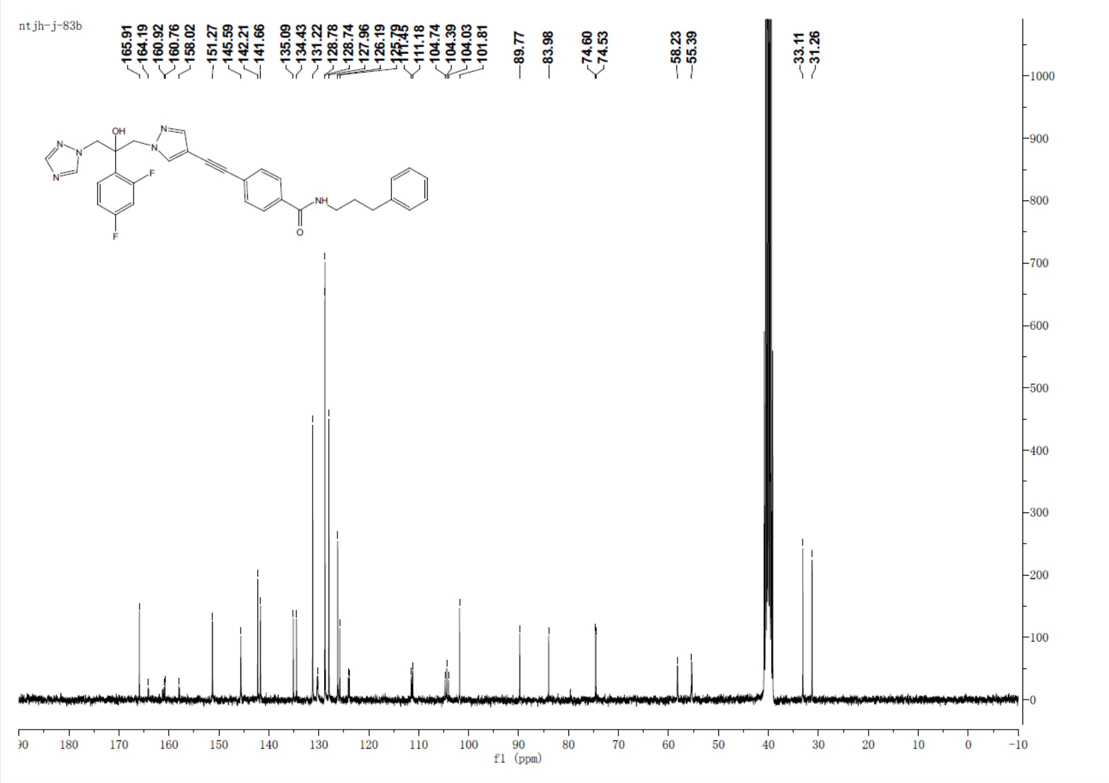

^{13}C NMR Spectrum of Compound **5n** (75 MHz, DMSO-*d6*)

Qualitative Analysis Report

**Data Filename**	J-83B.d	**Sample Name**	
**Sample Type**	Sample	**Position**	P1-A9
**Instrument Name**	Instrument 1	**User Name**	
**Acq Method**	TEST-POS-WL.m	**Acquired Time**	9/29/2019 9:15:57 AM
**IRM Calibration Status**	Success	**DA Method**	SERUM-POS-19MIN.m
**Comment**			

Sample Group **Info.**

User Spectra

Fragmentor Voltage	Collision Energy	Ionization Mode
120	0	ESI

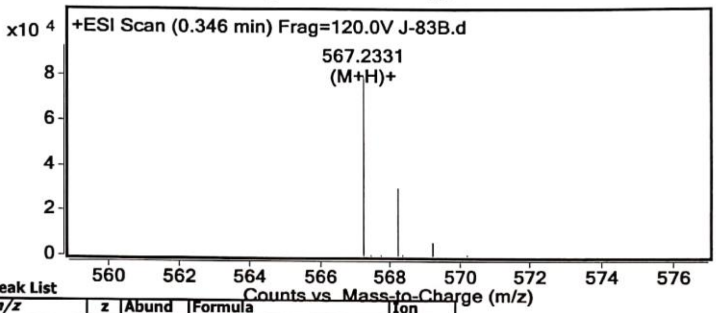

Peak List

m/z	z	Abund	Formula	Ion
121.0509		11484.6		
130.1584		16250.9		
158.1533		15002		
284.1195	2	25571.8		
284.6217	2	9150.5		
302.2472		8661.7		
567.2331	1	80243	C32 H29 F2 N6 O2	(M+H)+
568.2354	1	30483.7	C32 H29 F2 N6 O2	(M+H)+
589.2145		6316.2		
922.0098		14068.6		

Formula Calculator Element Limits

Element	Min	Max
C	0	100
H	0	150
O	2	2
N	6	6
F	2	2

Formula Calculator Results

Formula	Best	Mass	Tgt Mass	Diff (ppm)	Ion Species	Score
C32 H28 F2 N6 O2	TRUE	566.2258	566.2242	-2.8	C32 H29 F2 N6 O2	94.67

--- End Of Report ---

HRMS Spectrum of Compound **5n**

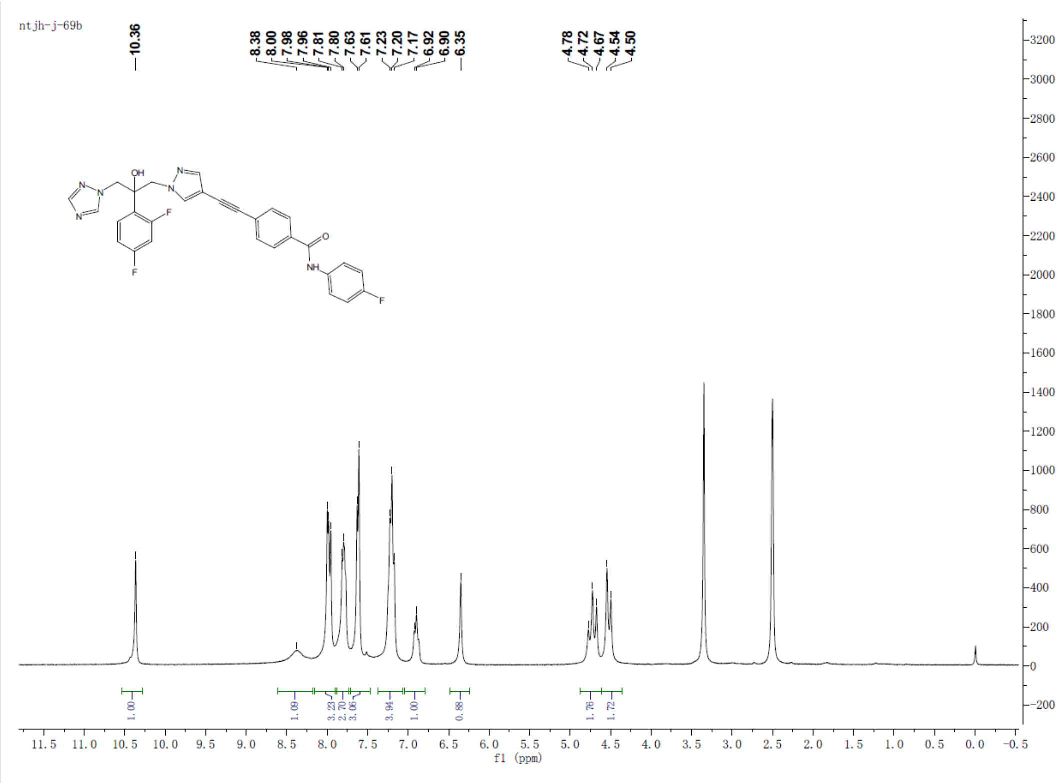

^{1}H NMR Spectrum of Compound **5o** (300 MHz, DMSO-d_6)

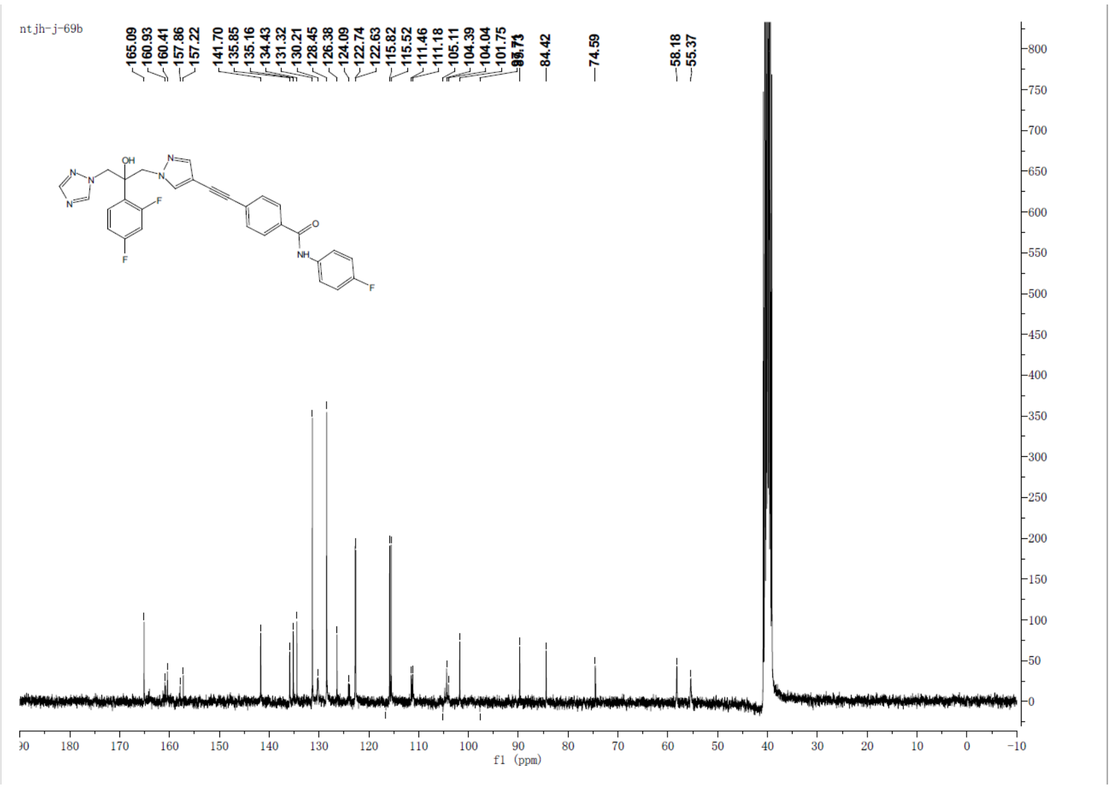

^{13}C NMR Spectrum of Compound **5o** (75 MHz, DMSO-d_6)

Qualitative Analysis Report

**Data Filename**	J-69B.d	**Sample Name**	
**Sample Type**	Sample	**Position**	P1-B1
**Instrument Name**	Instrument 1	**User Name**	
**Acq Method**	TEST-POS-WL.m	**Acquired Time**	9/29/2019 9:17:33 AM
**IRM Calibration Status**	Success	**DA Method**	SERUM-POS-19MIN.m
**Comment**			

Sample Group **Info.**

User Spectra

Fragmentor Voltage	Collision Energy	Ionization Mode
120	0	ESI

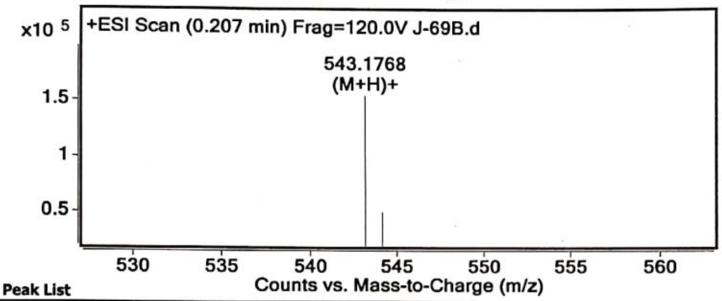

Peak List

m/z	z	Abund	Formula	Ion
121.0509		9809.5		
125.9857		8683.7		
130.1592		16461.4		
158.1541		13058.1		
437.1935		8895.4		
543.1768	1	154807	C29 H22 F3 N6 O2	(M+H)+
544.18	1	50318	C29 H22 F3 N6 O2	(M+H)+
545.1795	1	9041.7	C29 H22 F3 N6 O2	(M+H)+
619.5278		8157.5		
922.0098		12902		

Formula Calculator Element Limits

Element	Min	Max
C	0	100
H	0	150
O	2	2
N	6	6
F	3	3

Formula Calculator Results

Formula	Best	Mass	Tgt Mass	Diff (ppm)	Ion Species	Score
C29 H21 F3 N6 O2	TRUE	542.1695	542.1678	-3.19	C29 H22 F3 N6 O2	93.31

--- End Of Report ---

HRMS Spectrum of Compound **5o**

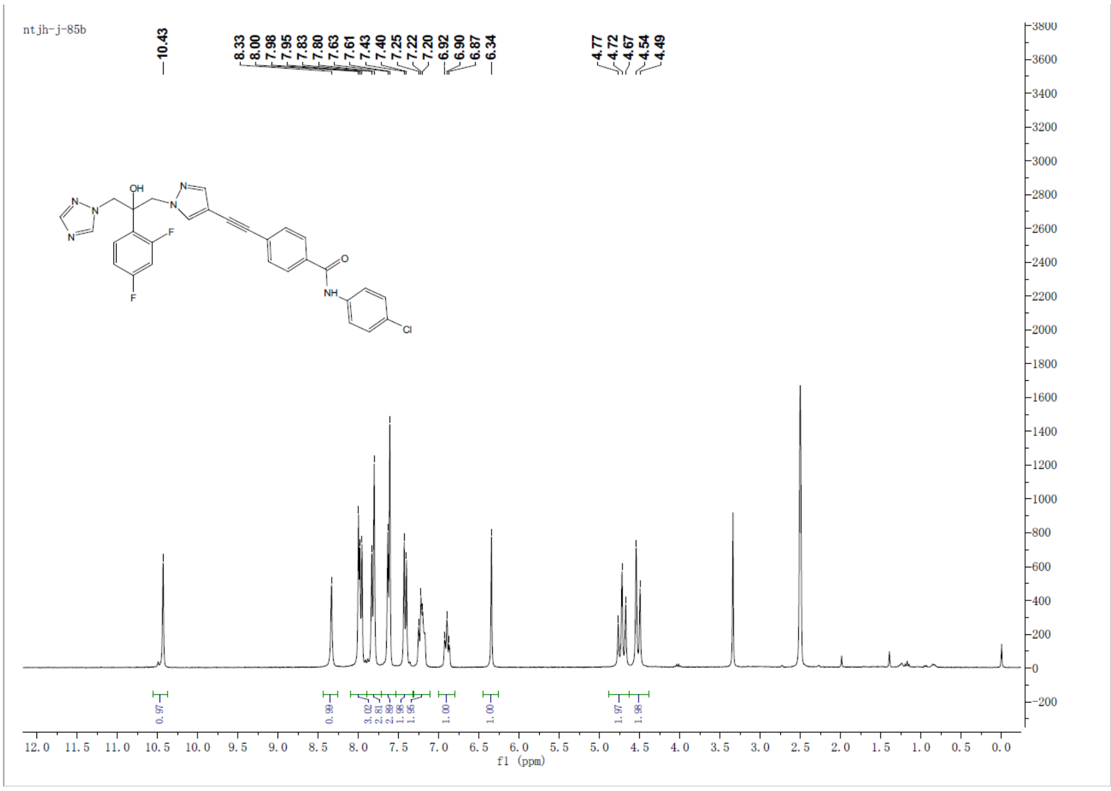

^{1}H NMR Spectrum of Compound **5p** (300 MHz, DMSO-d_6)

Qualitative Analysis Report

**Data Filename**	J-85B.d	**Sample Name**	
**Sample Type**	Sample	**Position**	P1-D1
**Instrument Name**	Instrument 1	**User Name**	
**Acq Method**	TEST-POS-WL.m	**Acquired Time**	9/29/2019 9:46:27 AM
**IRM Calibration Status**	Success	**DA Method**	SERUM-POS-19MIN.m
**Comment**			

Sample Group **Info.**

User Spectra

Fragmentor Voltage	Collision Energy	Ionization Mode
120	0	ESI

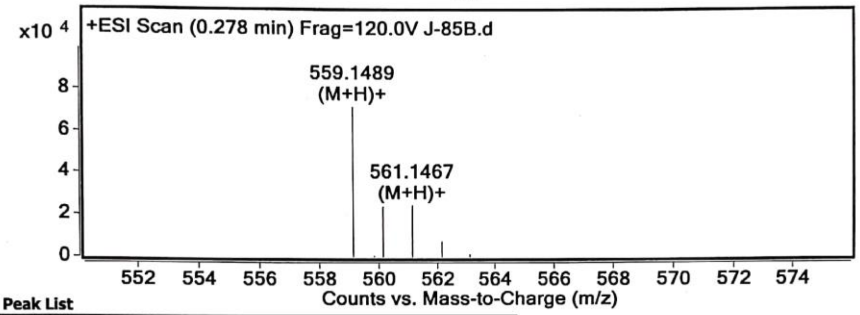

Peak List

m/z	z	Abund	Formula	Ion
158.154		8723.5		
559.147	1	172141.3	C29 H22 Cl F2 N6 O2	(M+H)+
560.1491	1	53746.9	C29 H22 Cl F2 N6 O2	(M+H)+
561.144	1	60668	C29 H22 Cl F2 N6 O2	(M+H)+
562.1453	1	16834	C29 H22 Cl F2 N6 O2	(M+H)+
609.1647		10709		

Formula Calculator Element Limits

Element	Min	Max
C	0	100
H	0	150
O	2	2
N	6	6
F	2	2
Cl	1	1

Formula Calculator Results

Formula	Best	Mass	Tgt Mass	Diff (ppm)	Ion Species	Score
C29 H21 Cl F2 N6 O2	TRUE	558.1397	558.1383	-2.5	C29 H22 Cl F2 N6 O2	94.58

--- End Of Report ---

HRMS Spectrum of Compound **5p**

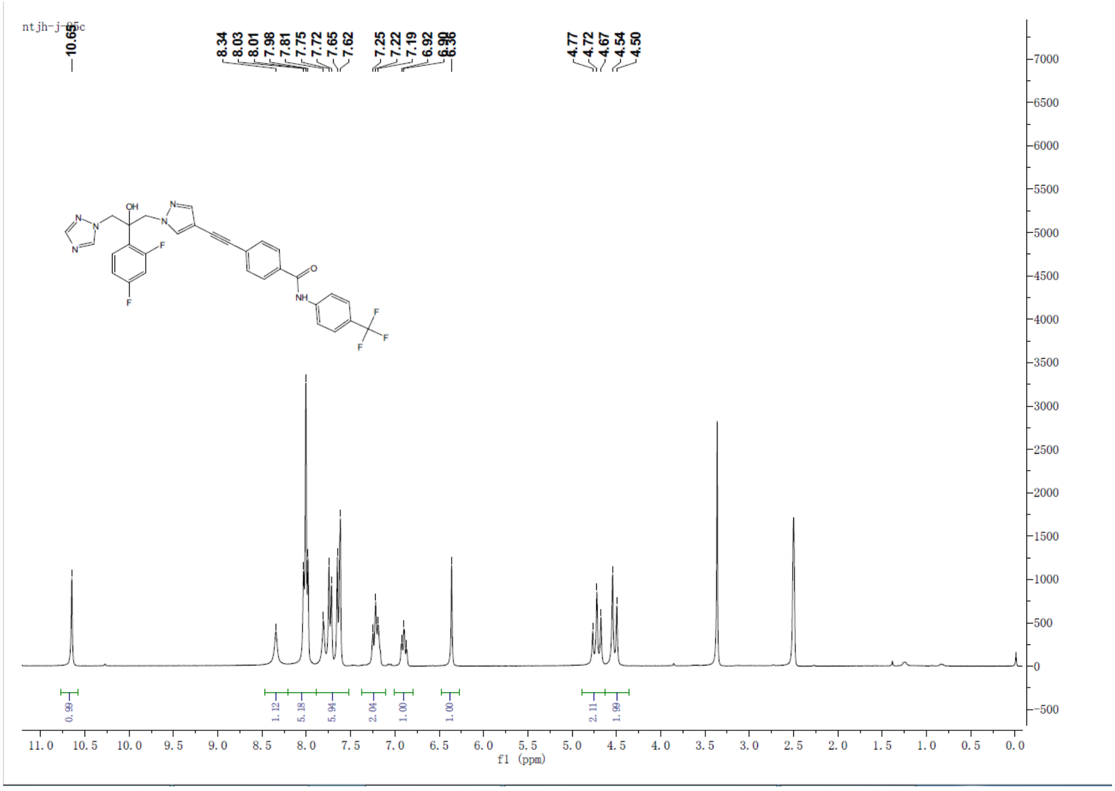

^{1}H NMR Spectrum of Compound **5q** (300 MHz, DMSO-d_6)

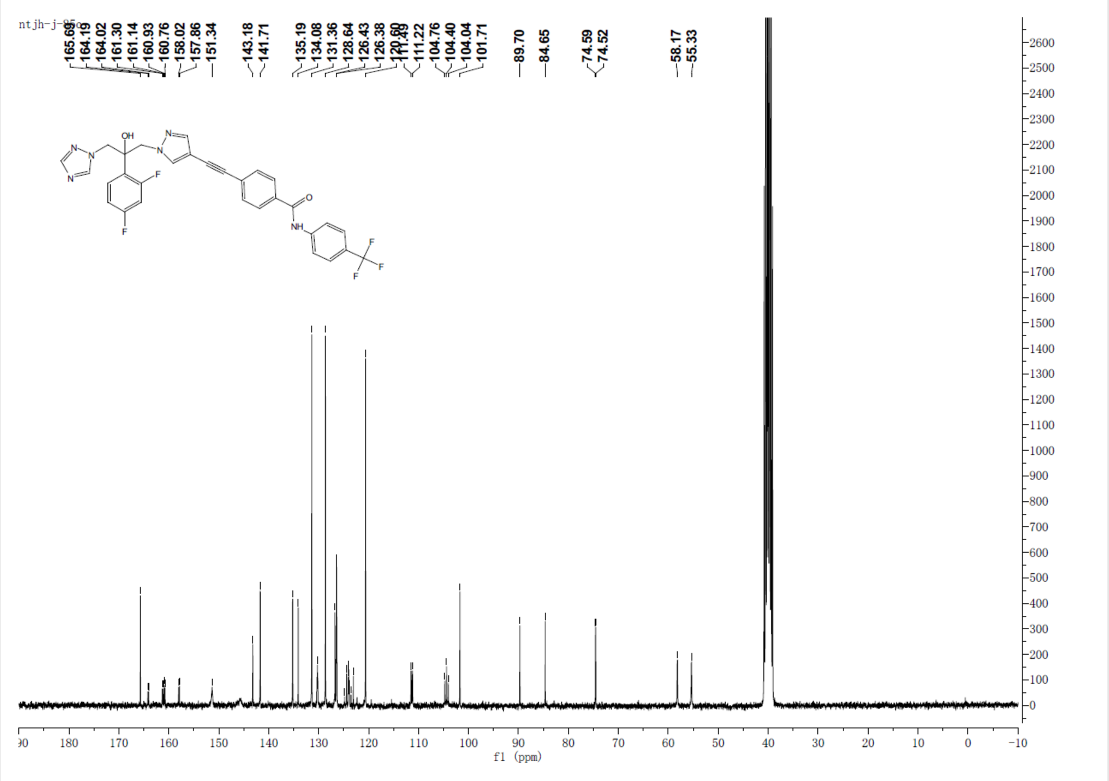

^{13}C NMR Spectrum of Compound **5q** (75 MHz, DMSO-d_6)

Qualitative Analysis Report

**Data Filename**	J-85C.d	**Sample Name**	
**Sample Type**	Sample	**Position**	P1-C9
**Instrument Name**	Instrument 1	**User Name**	
**Acq Method**	TEST-POS-WL.m	**Acquired Time**	9/29/2019 9:44:51 AM
**IRM Calibration Status**	Success	**DA Method**	SERUM-POS-19MIN.m
**Comment**			

Sample Group **Info.**

User Spectra

Fragmentor Voltage	Collision Energy	Ionization Mode
120	0	ESI

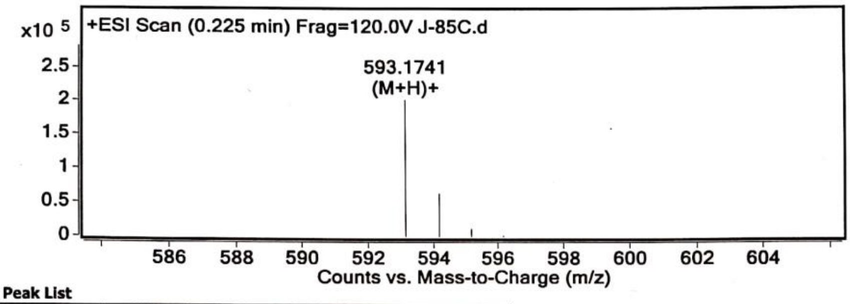

Peak List

m/z	z	Abund	Formula	Ion
130.16		18153.1		
158.1539		11409.6		
593.1741	1	202779.4	C30 H22 F5 N6 O2	(M+H)+
594.1763	1	63415.6	C30 H22 F5 N6 O2	(M+H)+
595.1787	1	11561.8	C30 H22 F5 N6 O2	(M+H)+

Formula Calculator Element Limits

Element	Min	Max
C	0	100
H	0	150
O	2	2
N	6	6
F	5	5

Formula Calculator Results

Formula	Best	Mass	Tgt Mass	Diff (ppm)	Ion Species	Score
C30 H21 F5 N6 O2	TRUE	592.1668	592.1646	-3.67	C30 H22 F5 N6 O2	90.35

--- End Of Report ---

HRMS Spectrum of Compound **5q**

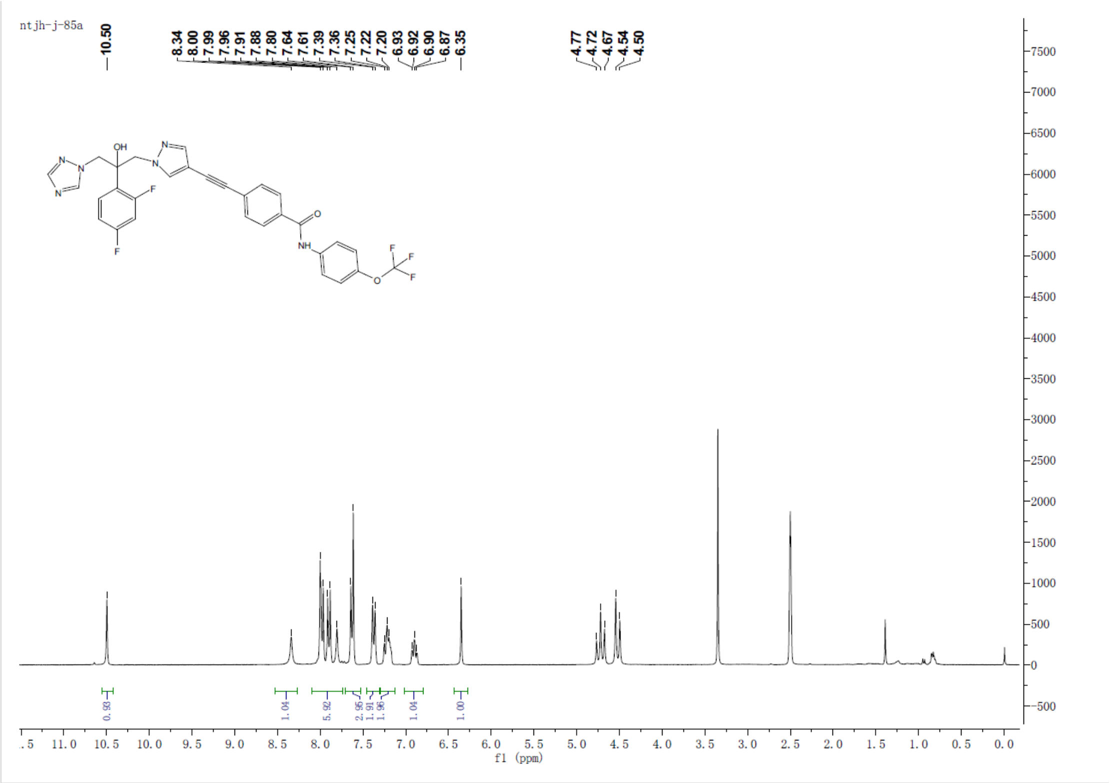

^{1}H NMR Spectrum of Compound **5r** (300 MHz, DMSO-d_6)

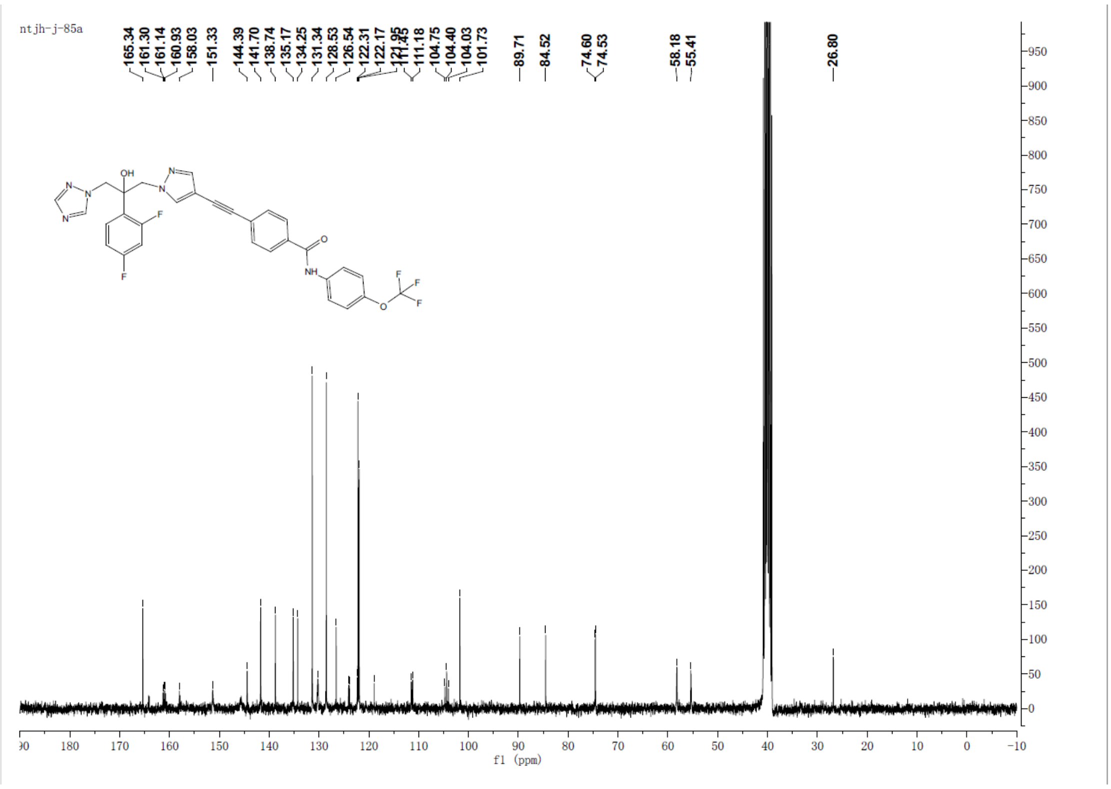

^{13}C NMR Spectrum of Compound **5r** (75 MHz, DMSO-d_6)

Qualitative Analysis Report

**Data Filename**	J-85A.d	**Sample Name**	
**Sample Type**	Sample	**Position**	P1-C4
**Instrument Name**	Instrument 1	**User Name**	
**Acq Method**	TEST-POS-WL.m	**Acquired Time**	9/29/2019 9:36:44 AM
**IRM Calibration Status**	Some Ions Missed	**DA Method**	SERUM-POS-19MIN.m
**Comment**			

Sample Group **Info.**

User Spectra

Fragmentor Voltage	Collision Energy	Ionization Mode
120	0	ESI

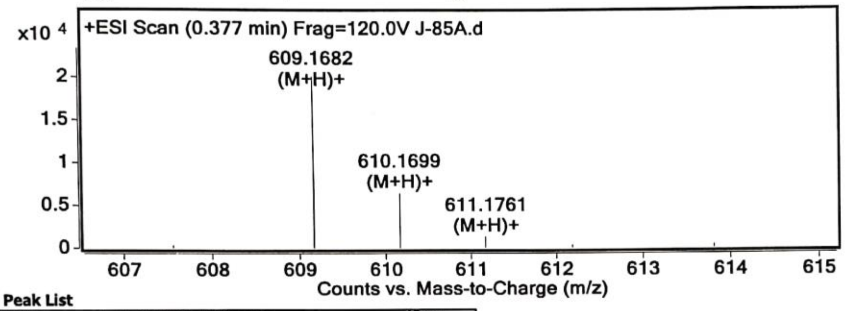

Peak List

m/z	z	Abund	Formula	Ion
121.0509		5262.8		
122.0561		4599		
125.9859		2943		
130.1586		8134.8		
158.1534	1	11050.5		
302.2503		2826.5		
609.1682	1	20157.3	C30 H22 F5 N6 O3	(M+H)+
610.1699	1	6392.6	C30 H22 F5 N6 O3	(M+H)+
922.0098	1	9013.1		
943.9922		3402.9		

Formula Calculator Element Limits

Element	Min	Max
C	0	100
H	0	150
O	3	3
N	6	6
F	5	5

Formula Calculator Results

Formula	Best	Mass	Tgt Mass	Diff (ppm)	Ion Species	Score
C30 H21 F5 N6 O3	TRUE	608.1609	608.1595	-2.3	C30 H22 F5 N6 O3	93.69

--- End Of Report ---

HRMS Spectrum of Compound **5r**

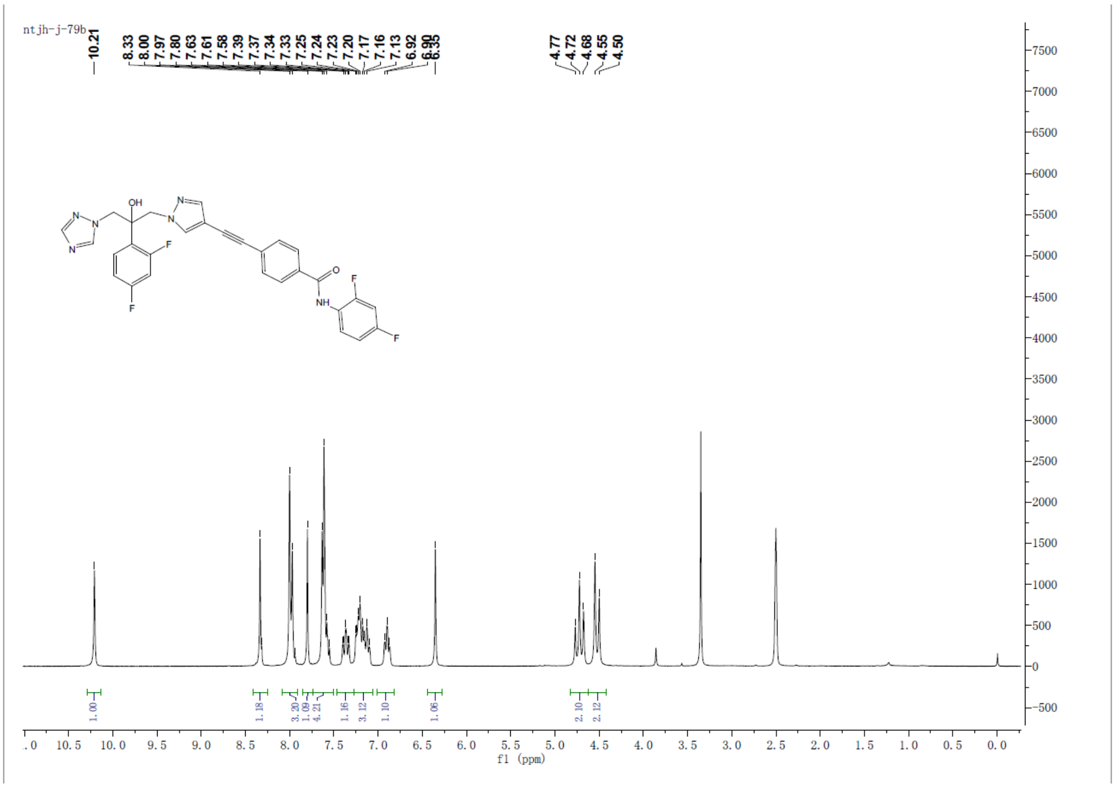

^{1}H NMR Spectrum of Compound **5s** (300 MHz, DMSO-d_6)

Qualitative Analysis Report

**Data Filename**	J-79B.d	**Sample Name**	
**Sample Type**	Sample	**Position**	P1-C6
**Instrument Name**	Instrument 1	**User Name**	
**Acq Method**	TEST-POS-WL.m	**Acquired Time**	9/29/2019 9:39:58 AM
**IRM Calibration Status**	Success	**DA Method**	SERUM-POS-19MIN.m
**Comment**			

Sample Group **Info.**

User Spectra

Fragmentor Voltage	Collision Energy	Ionization Mode
120	0	ESI

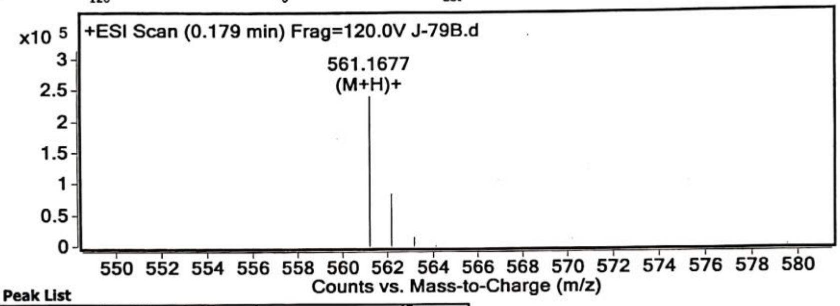

Peak List

m/z	z	Abund	Formula	Ion
338.3419		37765.5		
464.1522		22921.3		
561.1677	1	241584	C29 H21 F4 N6 O2	(M+H)+
562.1696	1	85476.2	C29 H21 F4 N6 O2	(M+H)+
563.1715	1	15632.7	C29 H21 F4 N6 O2	(M+H)+

Formula Calculator Element Limits

Element	Min	Max
C	0	100
H	0	150
O	2	2
N	6	6
F	4	4

Formula Calculator Results

Formula	Best	Mass	Tgt Mass	Diff (ppm)	Ion Species	Score
C29 H20 F4 N6 O2	TRUE	560.1604	560.1584	-3.62	C29 H21 F4 N6 O2	91.15

--- End Of Report ---

HRMS Spectrum of Compound **5s**

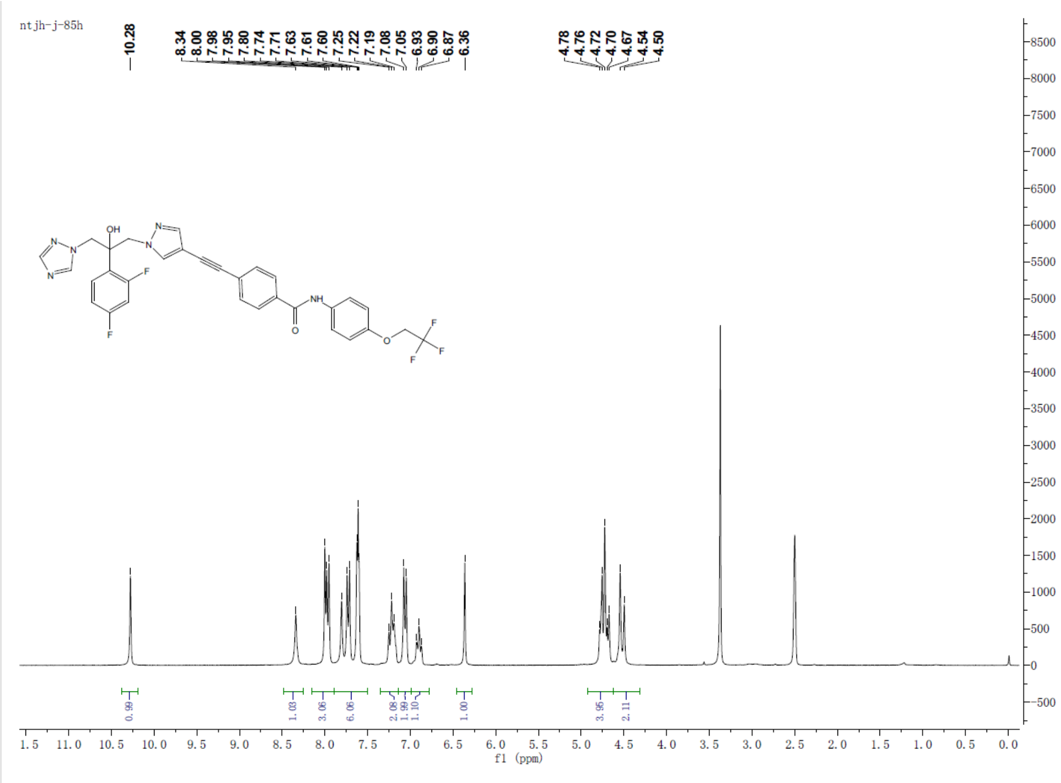

^{1}H NMR Spectrum of Compound **5t** (300 MHz, DMSO-*d6*)

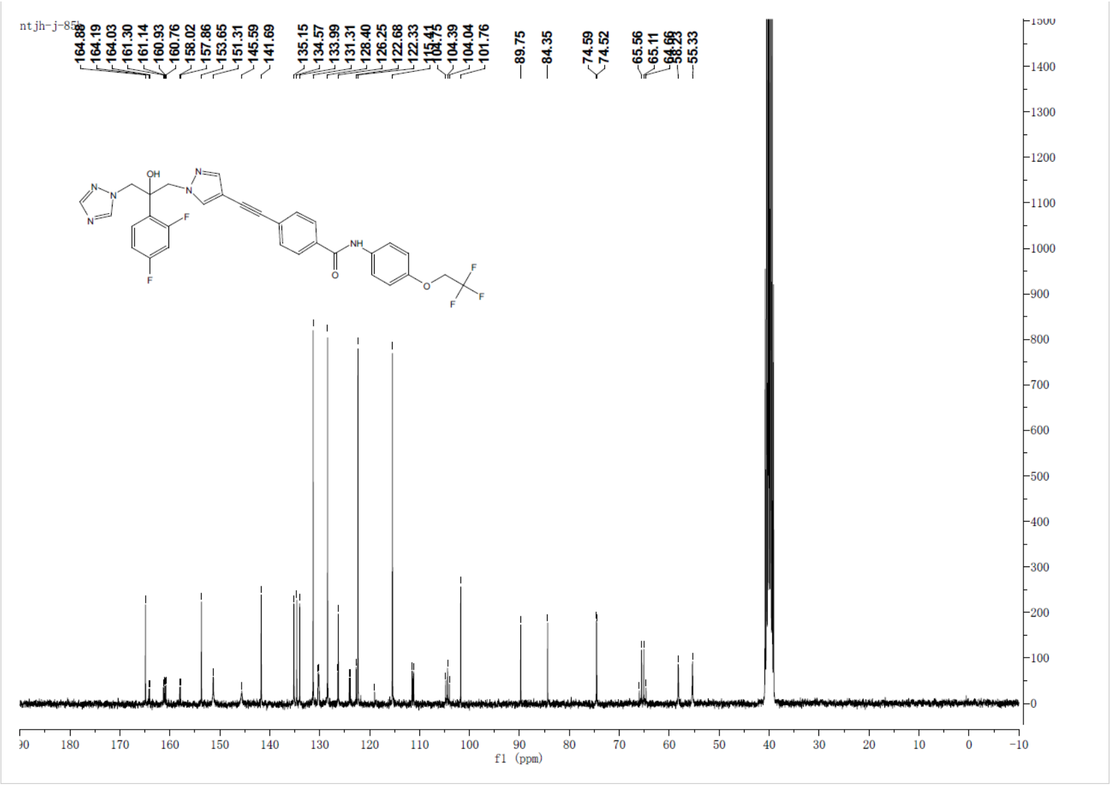

^{13}C NMR Spectrum of Compound **5t** (75 MHz, DMSO-*d6*)

Qualitative Analysis Report

**Data Filename**	J-85H.d	**Sample Name**	
**Sample Type**	Sample	**Position**	P1-B7
**Instrument Name**	Instrument 1	**User Name**	
**Acq Method**	TEST-POS-WL.m	**Acquired Time**	9/29/2019 9:27:10 AM
**IRM Calibration Status**	Success	**DA Method**	SERUM-POS-19MIN.m
**Comment**			

Sample Group **Info.**

User Spectra

Fragmentor Voltage	Collision Energy	Ionization Mode
120	0	ESI

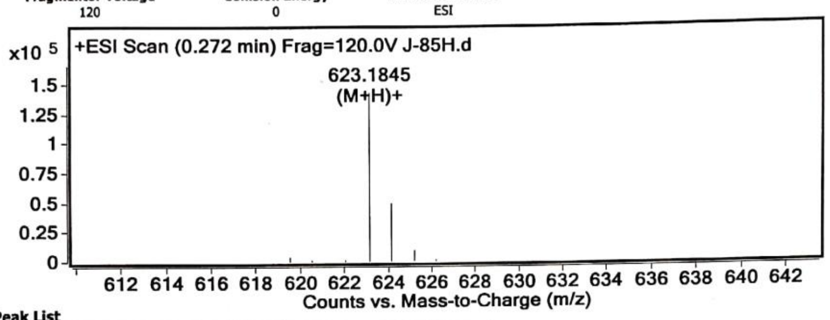

Peak List

m/z	z	Abund	Formula	Ion
130.158		8237.6		
158.1531		11915.2		
312.0943		7324.1		
623.1845	1	143341.9	C31 H24 F5 N6 O3	(M+H)+
624.1874	1	49167	C31 H24 F5 N6 O3	(M+H)+
625.1904	1	9093.7	C31 H24 F5 N6 O3	(M+H)+
922.0098		9133.1		

Formula Calculator Element Limits

Element	Min	Max
C	0	100
H	0	150
O	3	3
N	6	6
F	5	5

Formula Calculator Results

Formula	Best	Mass	Tgt Mass	Diff (ppm)	Ion Species	Score
C31 H23 F5 N6 O3	TRUE	622.1772	622.1752	-3.24	C31 H24 F5 N6 O3	93.49

--- End Of Report ---

HRMS Spectrum of Compound **5t**

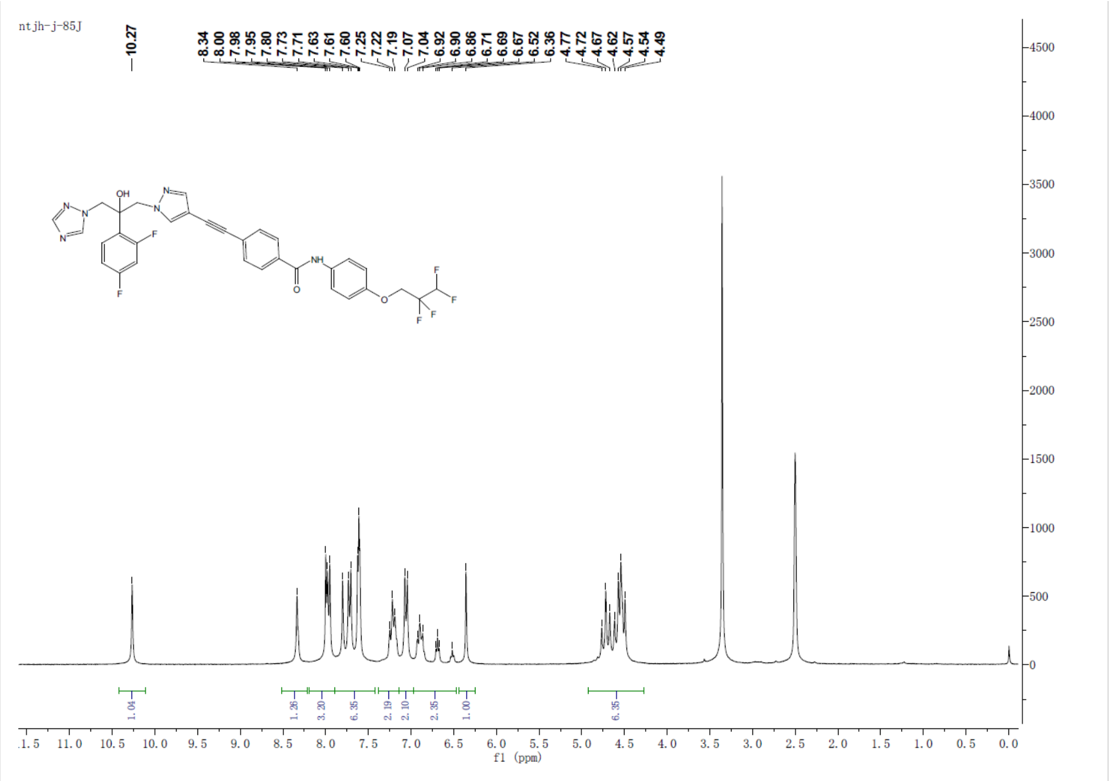

^{1}H NMR Spectrum of Compound **5u** (300 MHz, DMSO-d_6)

Qualitative Analysis Report

**Data Filename**	J-85J.d	**Sample Name**	
**Sample Type**	Sample	**Position**	P1-A4
**Instrument Name**	Instrument 1	**User Name**	
**Acq Method**	TEST-POS-WL.m	**Acquired Time**	9/29/2019 9:07:55 AM
**IRM Calibration Status**	Success	**DA Method**	SERUM-POS-19MIN.m
**Comment**			

Sample Group **Info.**

User Spectra

Fragmentor Voltage	Collision Energy	Ionization Mode
120	0	ESI

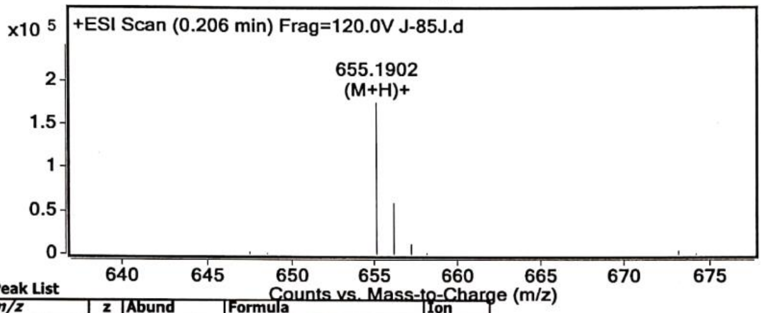

Peak List

m/z	z	Abund	Formula	Ion
121.0509		18437.1		
130.1588		31672.9		
158.1539		18724.4		
282.2788		32189.9		
302.2481		20028.9		
437.193		18935.9		
655.1902	1	176791.1	C32 H25 F6 N6 O3	(M+H)+
656.1924	1	60099.5	C32 H25 F6 N6 O3	(M+H)+
657.1943	1	12148.7	C32 H25 F6 N6 O3	(M+H)+
922.0098		20682.6		

Formula Calculator Element Limits

Element	Min	Max
C	0	100
H	0	150
O	3	3
N	6	6
S	0	0
F	6	6

Formula Calculator Results

Formula	Best	Mass	Tgt Mass	Diff (ppm)	Ion Species	Score
C32 H24 F6 N6 O3	TRUE	654.1829	654.1814	-2.31	C32 H25 F6 N6 O3	94.67

--- End Of Report ---

HRMS Spectrum of Compound **5u**

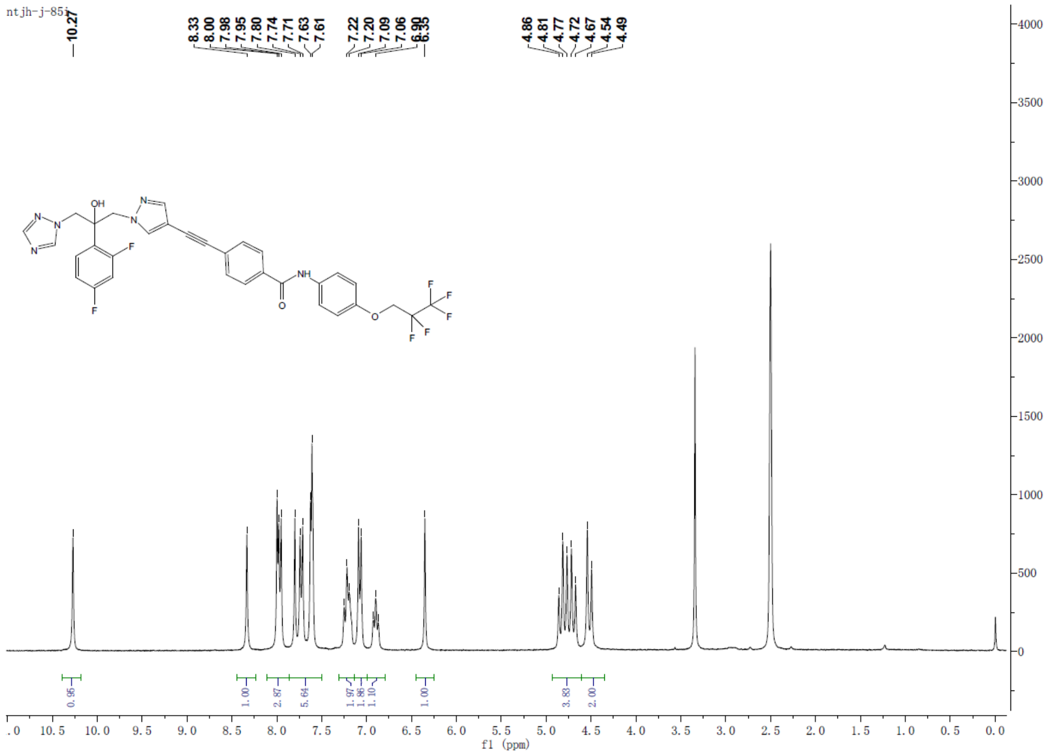

^{1}H NMR Spectrum of Compound **5v** (300 MHz, DMSO-d_6)

Qualitative Analysis Report

**Data Filename**	J-85I.d	**Sample Name**	
**Sample Type**	Sample	**Position**	P1-A8
**Instrument Name**	Instrument 1	**User Name**	
**Acq Method**	TEST-POS-WL.m	**Acquired Time**	9/29/2019 9:14:21 AM
**IRM Calibration Status**	Success	**DA Method**	SERUM-POS-19MIN.m
**Comment**			

Sample Group **Info.**

User Spectra

Fragmentor Voltage	Collision Energy	Ionization Mode
120	0	ESI

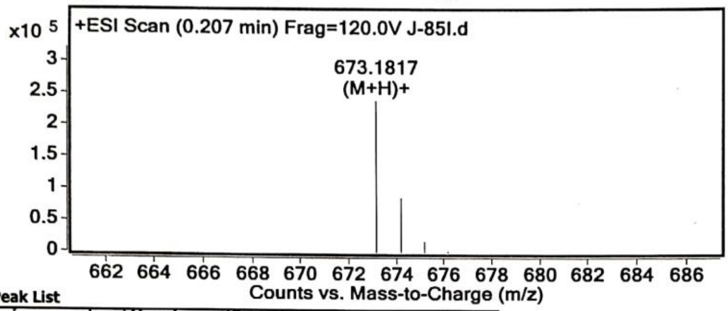

Peak List

m/z	z	Abund	Formula	Ion
130.1587		17463.8		
158.1534		12310.7		
337.0934		13016.9		
338.3414		29794.6		
437.194		17558.8		
673.1817	1	239441.7	C32 H24 F7 N6 O3	(M+H)+
674.1838	1	86295.9	C32 H24 F7 N6 O3	(M+H)+
675.1862	1	16762	C32 H24 F7 N6 O3	(M+H)+
922.0098		13750.7		

Formula Calculator Element Limits

Element	Min	Max
C	0	100
H	0	150
O	3	3
N	6	6
F	7	7

Formula Calculator Results

Formula	Best	Mass	Tgt Mass	Diff (ppm)	Ion Species	Score
C32 H23 F7 N6 O3	TRUE	672.1744	672.172	-3.6	C32 H24 F7 N6 O3	91.48

--- End Of Report ---

HRMS Spectrum of Compound **5v**

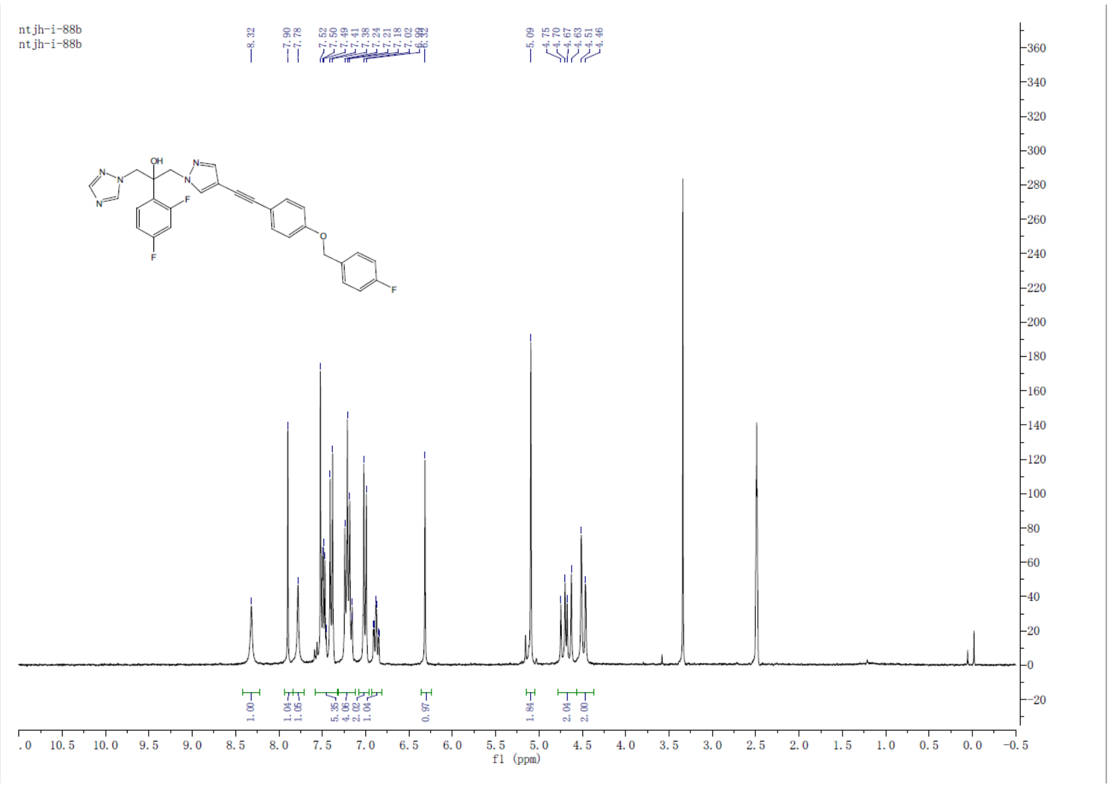

^{1}H NMR Spectrum of Compound **6a** (300 MHz, DMSO-d_6)

Qualitative Analysis Report

**Data Filename**	J-88B.d	**Sample Name**	
**Sample Type**	Sample	**Position**	P1-C2
**Instrument Name**	Instrument 1	**User Name**	
**Acq Method**	TEST-POS-WL.m	**Acquired Time**	9/29/2019 9:33:30 AM
**IRM Calibration Status**	Success	**DA Method**	SERUM-POS-19MIN.m
**Comment**			

Sample Group **Info.**

User Spectra

Fragmentor Voltage	Collision Energy	Ionization Mode
120	0	ESI

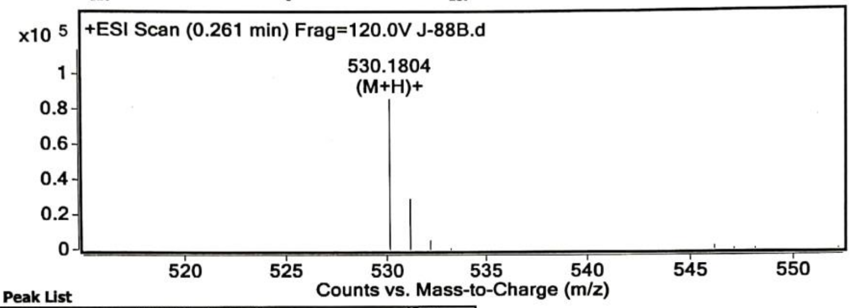

Peak List

m/z	z	Abund	Formula	Ion
121.0509		8319.1		
122.9632		4318.4		
130.1595		8709		
158.1527		13328.7		
530.1804	1	86201.9	C29 H23 F3 N5 O2	(M+H)+
531.1835	1	29084.1	C29 H23 F3 N5 O2	(M+H)+
532.1875	1	5199.6	C29 H23 F3 N5 O2	(M+H)+
596.1727		8166.2		
922.0098		10655.8		

Formula Calculator Element Limits

Element	Min	Max
C	0	100
H	0	150
O	2	2
N	5	5
F	3	3

Formula Calculator Results

Formula	Best	Mass	Tgt Mass	Diff (ppm)	Ion Species	Score
C29 H22 F3 N5 O2	TRUE	529.1732	529.1726	-1.13	C29 H23 F3 N5 O2	98.98

--- End Of Report ---

HRMS Spectrum of Compound **6a**

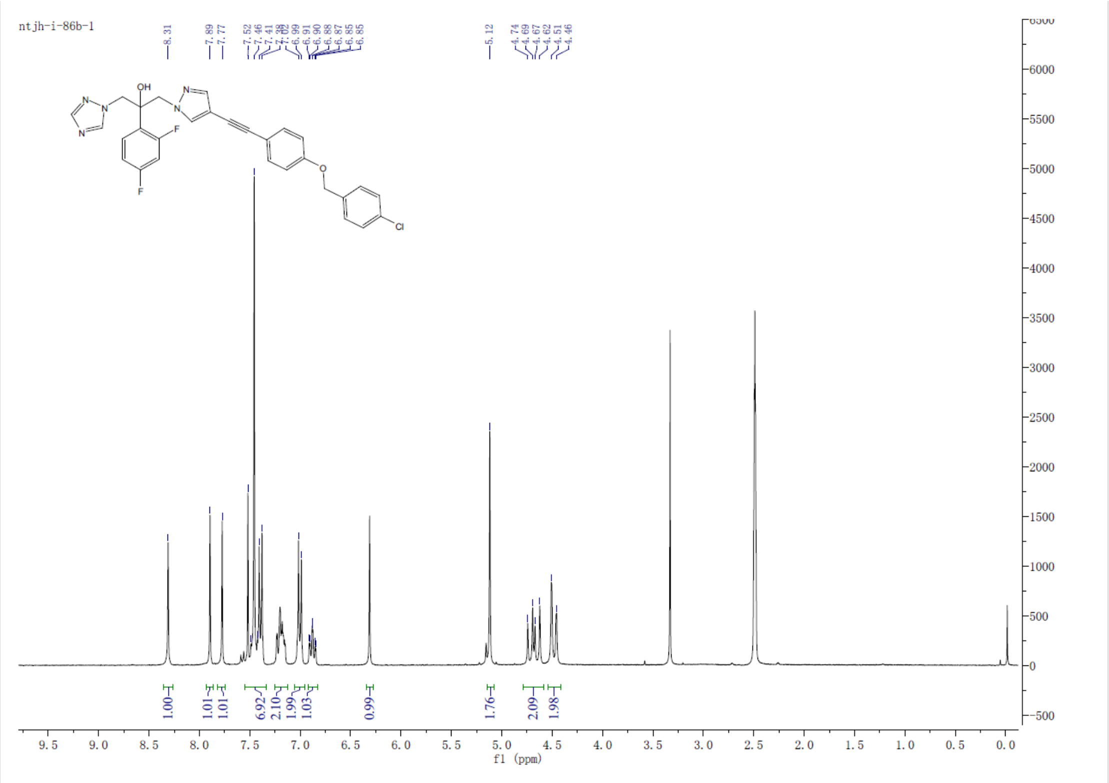

^{1}H NMR Spectrum of Compound **6b** (300 MHz, DMSO-d_6)

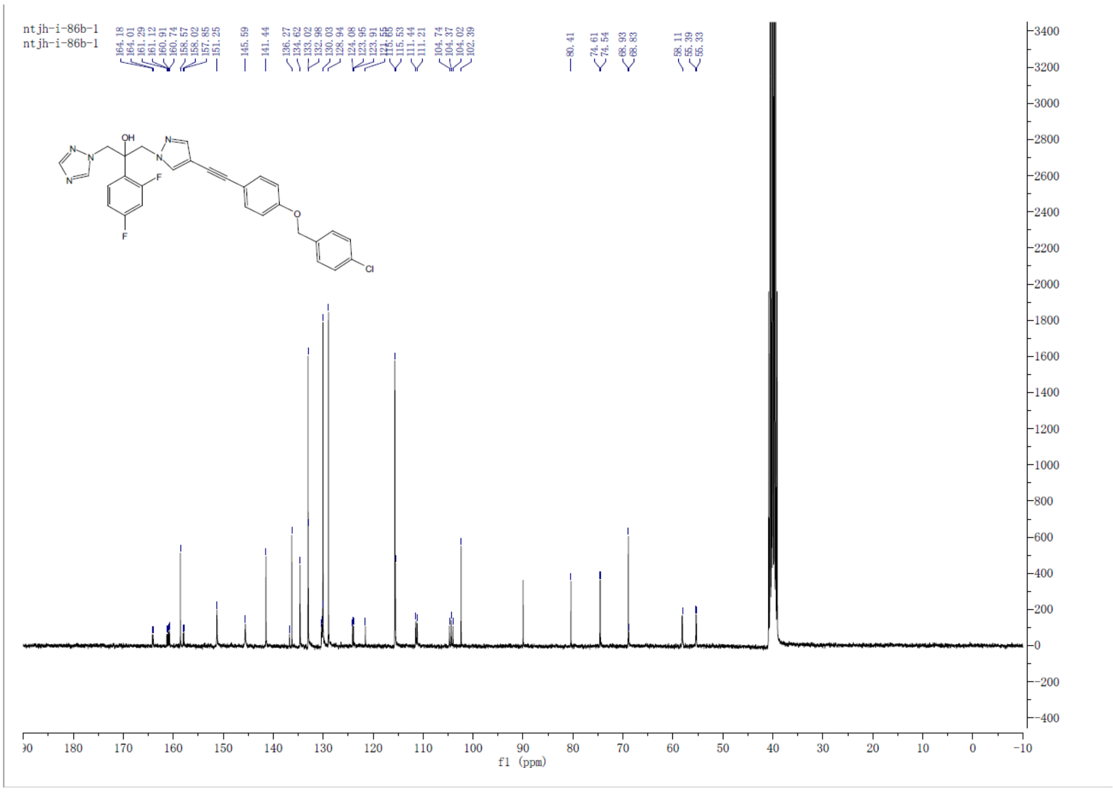

^{13}C NMR Spectrum of Compound **6b** (75 MHz, DMSO-d_6)

Qualitative Analysis Report

**Data Filename**	I-86B.d	**Sample Name**	
**Sample Type**	Sample	**Position**	P1-B8
**Instrument Name**	Instrument 1	**User Name**	
**Acq Method**	TEST-POS-WL.m	**Acquired Time**	9/29/2019 9:28:44 AM
**IRM Calibration Status**	Some Ions Missed	**DA Method**	SERUM-POS-19MIN.m
**Comment**			

Sample Group **Info.**

User Spectra

Fragmentor Voltage	Collision Energy	Ionization Mode
120	0	ESI

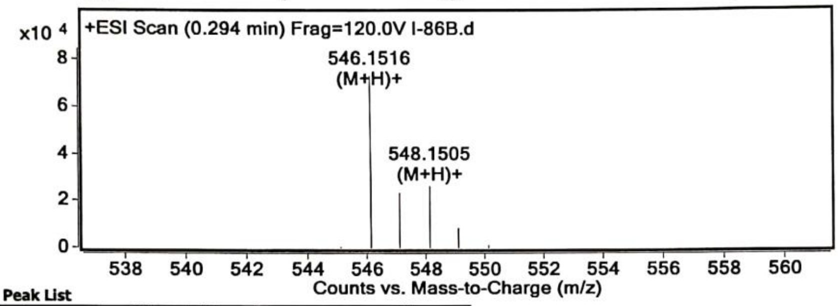

Peak List

m/z	z	Abund	Formula	Ion
121.0509		5375.6		
125.986		4109.9		
130.1589		10680.3		
158.154		13102.6		
546.1516	1	72939.8	C29 H23 Cl F2 N5 O2	(M+H)+
547.1538	1	23639	C29 H23 Cl F2 N5 O2	(M+H)+
548.1505	1	26416.6	C29 H23 Cl F2 N5 O2	(M+H)+
549.1518	1	8632.1	C29 H23 Cl F2 N5 O2	(M+H)+
596.1707		9822.6		
922.0098		9174.2		

Formula Calculator Element Limits

Element	Min	Max
C	0	100
H	0	150
O	2	2
N	5	5
F	2	2
Cl	1	1

Formula Calculator Results

Formula	Best	Mass	Tgt Mass	Diff (ppm)	Ion Species	Score
C29 H22 Cl F2 N5 O2	TRUE	545.1443	545.143	-2.37	C29 H23 Cl F2 N5 O2	96.12

--- End Of Report ---

HRMS Spectrum of Compound **6b**

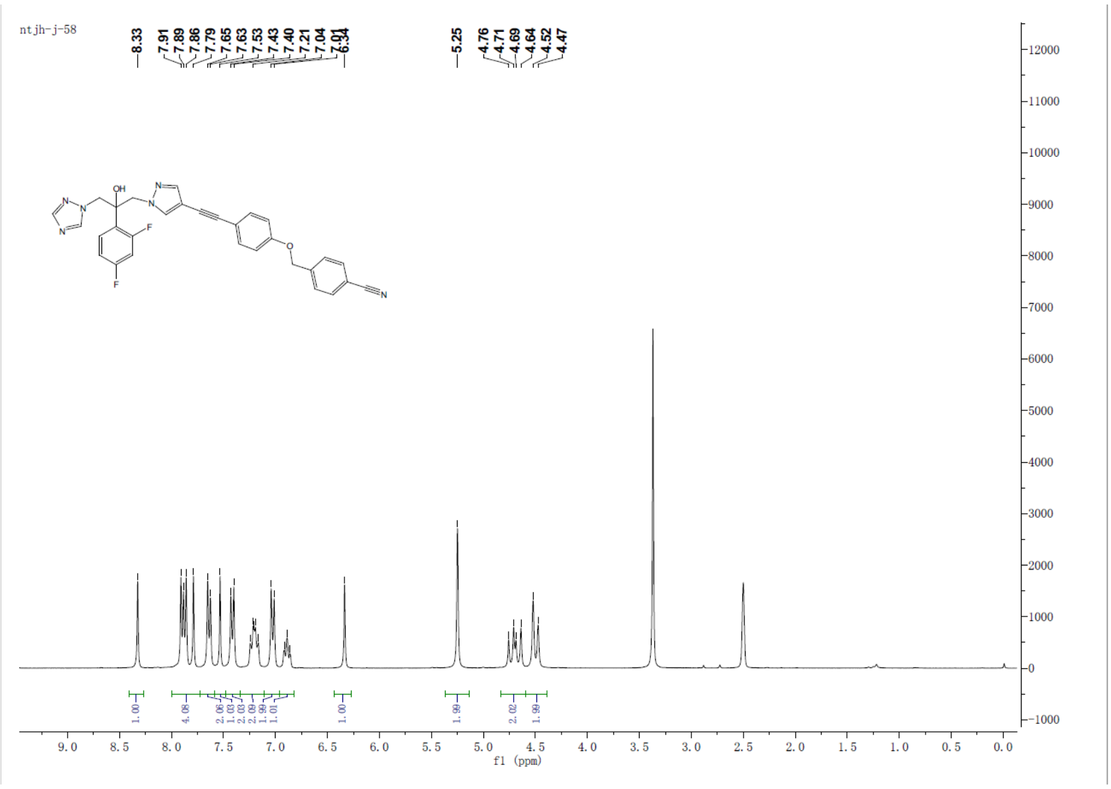

^{1}H NMR Spectrum of Compound **6c** (300 MHz, DMSO-d_6)

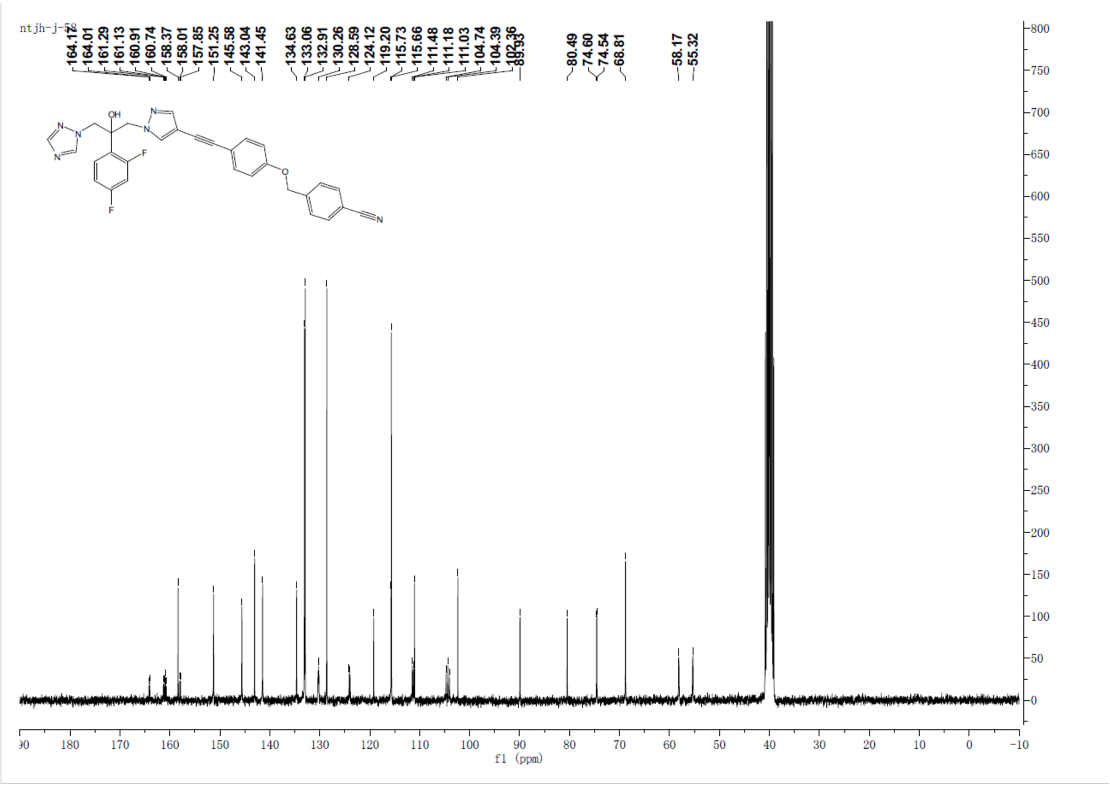

^{13}C NMR Spectrum of Compound **6c** (75 MHz, DMSO-d_6)

Qualitative Analysis Report

**Data Filename**	I-58.d	**Sample Name**	
**Sample Type**	Sample	**Position**	P1-B5
**Instrument Name**	Instrument 1	**User Name**	
**Acq Method**	TEST-POS-WL.m	**Acquired Time**	9/29/2019 9:23:56 AM
**IRM Calibration Status**	Success	**DA Method**	SERUM-POS-19MIN.m
**Comment**			

Sample Group **Info.**

User Spectra

Fragmentor Voltage	Collision Energy	Ionization Mode
120	0	ESI

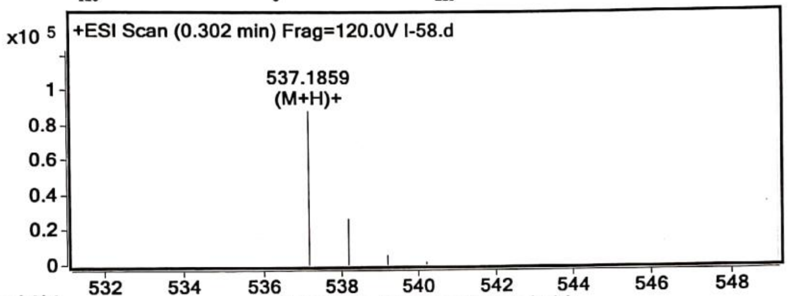

Peak List

m/z	z	Abund	Formula	Ion
121.0509		5801.8		
130.1585		9330.4		
158.1529		10588.4		
537.1859	1	88708.8	C30 H23 F2 N6 O2	(M+H)+
538.1889	1	26962.2	C30 H23 F2 N6 O2	(M+H)+
539.1905	1	5673.6	C30 H23 F2 N6 O2	(M+H)+
922.0098		9708.7		

Formula Calculator Element Limits

Element	Min	Max
C	0	100
H	0	150
O	2	2
N	6	6
F	2	2

Formula Calculator Results

Formula	Best	Mass	Tgt Mass	Diff (ppm)	Ion Species	Score
C30 H22 F2 N6 O2	TRUE	536.1786	536.1772	-2.65	C30 H23 F2 N6 O2	92.49

--- End Of Report ---

HRMS Spectrum of Compound **6c**

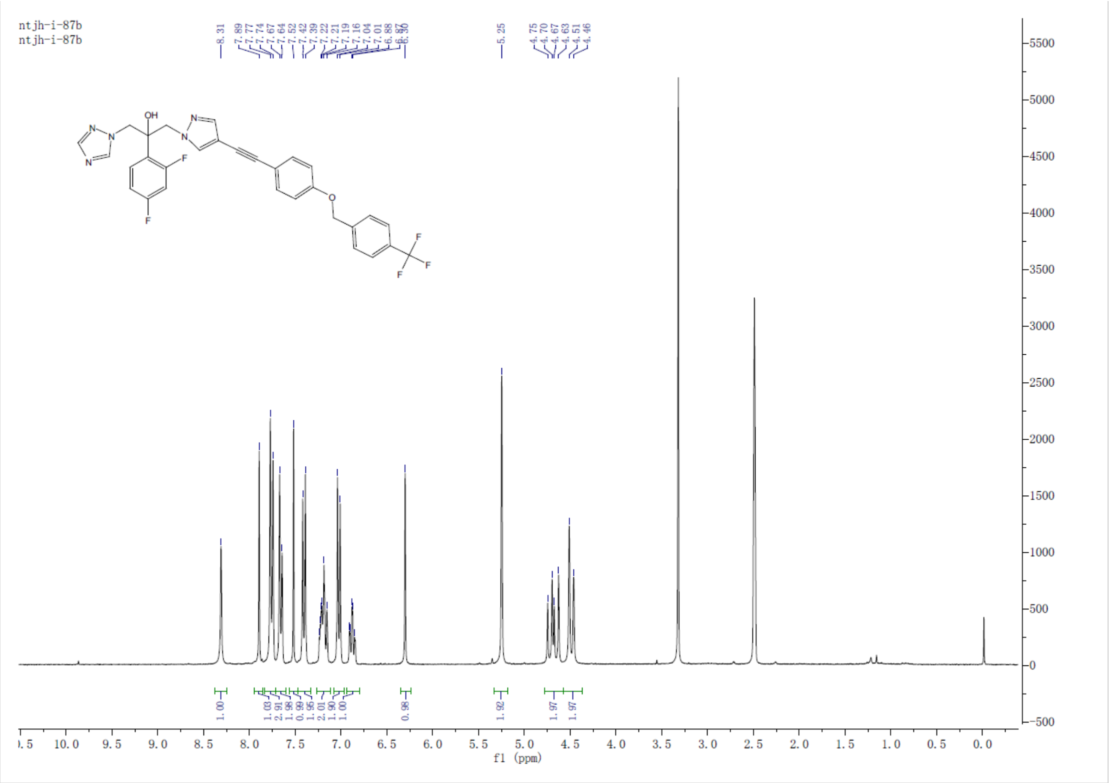

^{1}H NMR Spectrum of Compound **6d** (300 MHz, DMSO-*d*6)

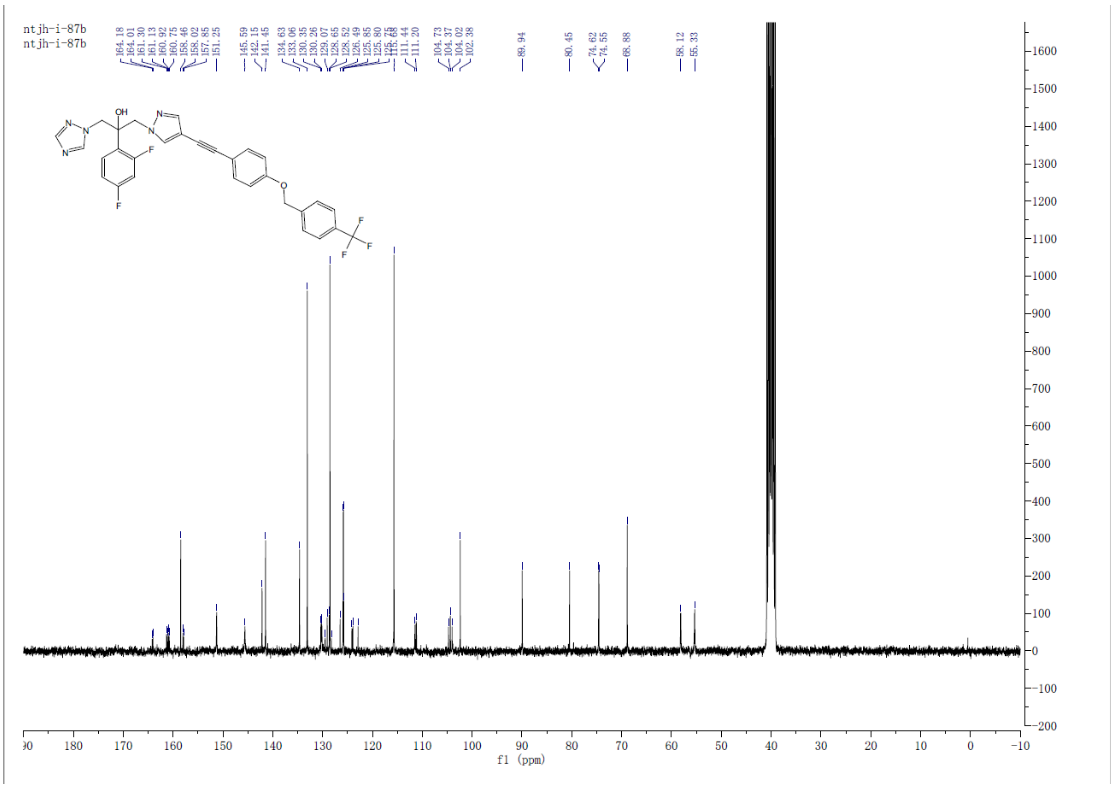

^{13}C NMR Spectrum of Compound **6d** (75 MHz, DMSO-*d*6)

Qualitative Analysis Report

**Data Filename**	I-87B.d	**Sample Name**	
**Sample Type**	Sample	**Position**	P1-B3
**Instrument Name**	Instrument 1	**User Name**	
**Acq Method**	TEST-POS-WL.m	**Acquired Time**	9/29/2019 9:20:46 AM
**IRM Calibration Status**	Some Ions Missed	**DA Method**	SERUM-POS-19MIN.m
**Comment**			

Sample Group **Info.**

User Spectra

Fragmentor Voltage	Collision Energy	Ionization Mode
120	0	ESI

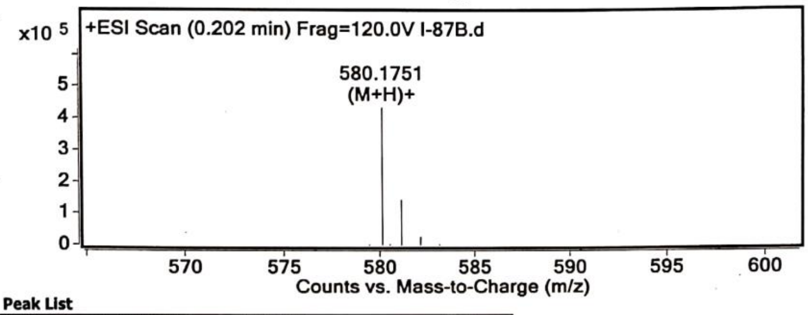

Peak List

m/z	z	Abund	Formula	Ion
338.34		32382.7		
580.1751	1	437299.2	C30 H23 F5 N5 O2	(M+H)+
581.1776	1	144717.4	C30 H23 F5 N5 O2	(M+H)+
582.1803	1	26533.5	C30 H23 F5 N5 O2	(M+H)+

Formula Calculator Element Limits

Element	Min	Max
C	0	100
H	0	150
O	2	2
N	5	5
F	5	5

Formula Calculator Results

Formula	Best	Mass	Tgt Mass	Diff (ppm)	Ion Species	Score
C30 H22 F5 N5 O2	TRUE	579.1679	579.1694	2.62	C30 H23 F5 N5 O2	95.51

--- End Of Report ---

HRMS Spectrum of Compound **6d**

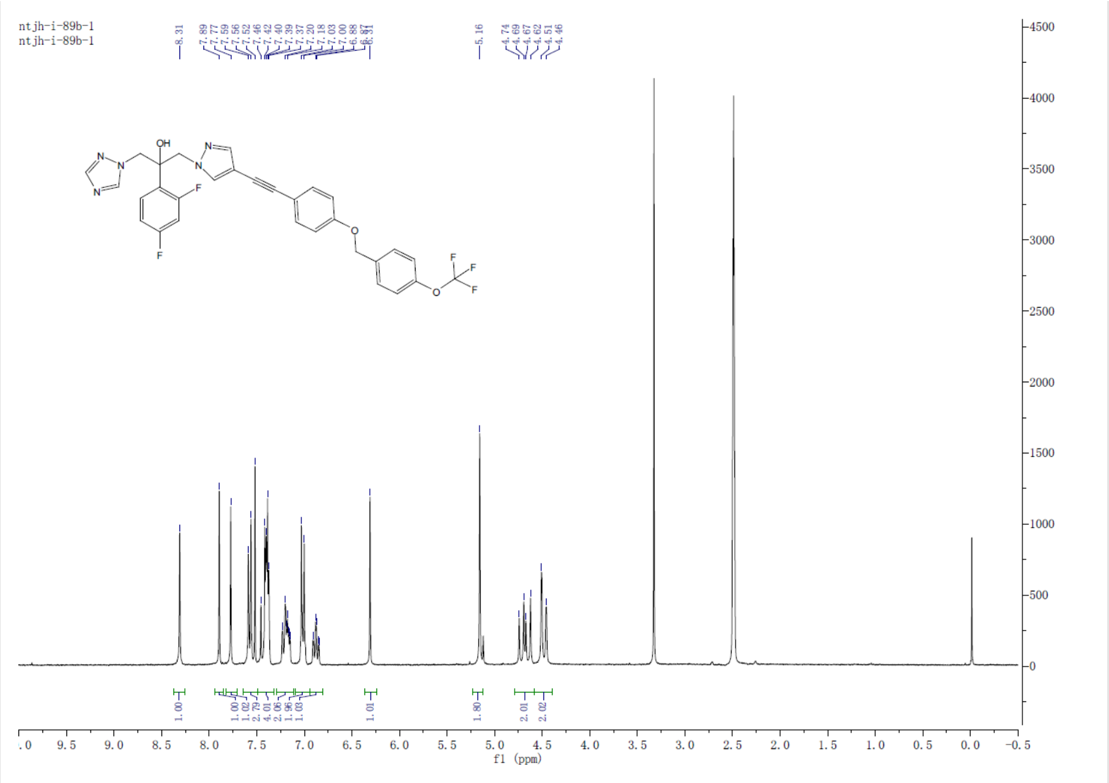

^{1}H NMR Spectrum of Compound **6e** (300 MHz, DMSO-d_6)

Qualitative Analysis Report

**Data Filename**	I-89B.d	**Sample Name**	
**Sample Type**	Sample	**Position**	P1-D3
**Instrument Name**	Instrument 1	**User Name**	
**Acq Method**	TEST-POS-WL.m	**Acquired Time**	9/29/2019 9:49:37 AM
**IRM Calibration Status**	Success	**DA Method**	SERUM-POS-19MIN.m
**Comment**			

Sample Group **Info.**

User Spectra

Fragmentor Voltage	Collision Energy	Ionization Mode
120	0	ESI

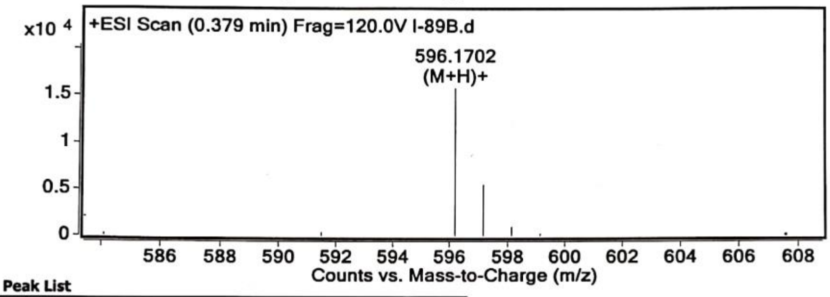

Peak List

m/z	z	Abund	Formula	Ion
122.0562		4208.5		
125.9853		2597.2		
130.0069		2265.4		
130.1581		6911.2		
158.1532	1	11084.4		
271.1871	1	2517.1		
302.2474		2245.6		
596.1702	1	15931.6	C30 H23 F5 N5 O3	(M+H)+
597.1742	1	5572.3	C30 H23 F5 N5 O3	(M+H)+
922.0098	1	3078.6		

Formula Calculator Element Limits

Element	Min	Max
C	0	100
H	0	150
O	3	3
N	5	5
F	5	5

Formula Calculator Results

Formula	Best	Mass	Tgt Mass	Diff (ppm)	Ion Species	Score
C30 H22 F5 N5 O3	TRUE	595.163	595.1643	2.18	C30 H23 F5 N5 O3	96.47

--- End Of Report ---

HRMS Spectrum of Compound **6e**
